# *Plasmodium falciparum* gene expression signatures and antibody profiling implicate the *fikk*, *phist*, and *surf* multigene families in severe malaria syndromes

**DOI:** 10.1016/j.jinf.2025.106655

**Published:** 2025-11-12

**Authors:** Bryan E. Cummings, Emily M. Stucke, Drissa Coulibaly, Jonathan G. Lawton, Rafal S. Sobota, Abdoulaye Kassoum Kone, Bourama Kane, Bouréima Guindo, Bourama Tangara, Mody Sissoko, Fayçal Maiga, Karim Traore, Aichatou Diawara, Amidou Traore, Ali Thera, Antoine Dara, Amadou Niangaly, Modibo Daou, Issa Diarra, Youssouf Tolo, Savy Sebastian, Aarti Jain, Rie Nakajima, Algis Jasinskas, Emily K. Silzel, Biraj Shrestha, Amed Ouattara, James B. Munro, Matthew B. Laurens, Kirsten E. Lyke, Kieran Tebben, Haikel Bogale, Christopher V. Plowe, Mahamadou S. Sissoko, Bourema Kouriba, Shannon Takala-Harrison, Philip L. Felgner, Joana C. Silva, Ogobara K. Doumbo, Mahamadou A. Thera, Mark A. Travassos

**Affiliations:** a Malaria Research Program, Center for Vaccine Development and Global Health, University of Maryland School of Medicine, 685 W Baltimore Street, Baltimore, MD, USA; b Malaria Research and Training Center, University of Sciences, Techniques and Technologies, BP 1805, Point G, Bamako, Mali; c Ken and Ruth Davee Department of Neurology, Northwestern University, 303 E. Chicago Avenue, Chicago, IL, USA; d Vaccine Research & Development Center, University of California, 825 Health Sciences Road, Irvine, CA, USA; e Institute for Genome Sciences, University of Maryland School of Medicine, 670 West Baltimore Street, Baltimore, MD, USA; f Department of Microbiology and Immunology, University of Maryland School of Medicine, 685 West Baltimore Street, Baltimore, MD, USA

**Keywords:** Cerebral malaria, Severe malarial anemia, *Plasmodium falciparum*, Differential expression, Vaccine candidates, Children, Mali, RNA sequencing, Protein microarray, Gene ontology

## Abstract

*Plasmodium falciparum* is the most virulent *Plasmodium* species, responsible for the life-threatening severe malaria syndromes of cerebral malaria and severe malarial anemia. In a Mali case-control study, we determined parasite gene expression in cerebral malaria, severe malarial anemia, and concurrent disease featuring both syndromes by comparing RNA-seq data from severe malaria cases to matched uncomplicated malaria controls with or without a history of cerebral malaria. While severe malarial anemia comparisons produced comparatively low levels of differential expression, the comparisons of cerebral malaria and concurrent disease yielded significant differential expression of multigene families (*fikk*, *phist*, and *surf*) implicated in processes that escalate disease severity, such as cytoadherence and erythrocytic remodeling. We probed custom microarrays featuring these antigens with acute and convalescent sera. Children with severe malaria developed antibody responses to subsets of these proteins following severe malaria infection, suggesting exposure to these antigens during illness and their potential as targets in the development of natural protective immunity against severe malaria. Our findings address knowledge gaps surrounding parasite gene expression in severe malarial anemia. Further, these results broaden the scope of multigene families implicated in severe malaria pathogenesis and underscore the utility of transcriptome-wide approaches for a comprehensive understanding of severe disease, particularly when developing therapeutics or vaccines.

## Introduction

Of the five parasite species known to cause human malaria disease, *Plasmodium falciparum* is the most virulent and is responsible for nearly all malaria-related deaths.^1^ In 2021, there were an estimated 247 million clinical malaria episodes and approximately 619,000 thousand deaths—mostly among children under the age of five in sub-Saharan Africa.^2^ Cerebral malaria (CM) is a severe malaria manifestation characterized by impaired consciousness that cannot be attributed to convulsions, sedative drugs, or hypoglycemia alone, nor to other non-malarial causes.^1^ Severe malarial anemia (SMA) is another form of severe malaria associated with an increased risk of death in children.^3^ SMA is the single greatest contributor to mortality for any malarial syndrome and is likely responsible for more than half of all malaria-associated deaths worldwide.^3^ CM and SMA disease can occur in isolation or concurrently.^4^ Mortality risk increases with concurrent severe malaria syndromes, resulting in neurological, metabolic, and respiratory dysfunction.^4–6^ The concurrent syndrome has been observed to have the highest mortality in Malian children,^6^ although it should be noted that children with anemia and impaired consciousness had a lower mortality rate when compared to impaired consciousness alone in a study in Kenya.^7^

Whereas CM has been the focus of animal models and pathophysiologic investigation, the exact pathophysiology of SMA is less understood. The causes of SMA appear to be multifactorial and may involve the destruction of circulating erythrocytes and reduced production of erythrocytes within the bone marrow.^5^ Consequently, children with SMA have evidence of dyserythropoiesis and inflammation and may require immediate transfusion.^5^ Host innate immune responses and inflammatory pathways are also thought to play a substantial role in SMA progression.^8^ *Plasmodium* infection stimulates the release of cytokines, chemokines, growth factors, and effector molecules that can induce damage to the host, resulting in impaired erythrocyte production.^8^ Other host factors including autoimmune antibody destruction of erythrocytes and congestive splenomegaly have also been implicated in the development of SMA.^9,10^ While host factors play a known role in SMA pathogenesis, the contribution of parasite processes remains unclear. A transcriptome-level analysis of parasite gene expression in SMA has not previously been done.

*P. falciparum* modifies the erythrocyte surface and induces cytoadherence to the host endothelium, enabling sequestration and avoidance of host immune responses.^11^ Cytoadherence and sequestration of infected erythrocytes in the host vasculature are important for CM pathogenesis and are considered central to *P. falciparum* virulence.^12^ Infected erythrocytes can adhere to receptors in end organs including the spleen, liver, intestines, placenta, and brain.^13^ Adherence to brain vasculature is believed to result in endothelial inflammation and disruption of the blood-brain barrier.^12^

Three multigene families that encode variant surface antigens–namely *var, rif,* and *stevor* – are believed to mediate infected erythrocyte adhesion to the vascular endothelium, to other healthy red blood cells (rosetting), and to leukocytes/serum proteins.^11^ Unfortunately, these established VSA gene families exhibit an extraordinary degree of sequence diversity, which complicates efforts to study their expression across different clinical isolates,^11,14^ although some recent work has attempted to do so.^15^ Furthermore, and due to these same limitations, transcription studies focusing on the 3D7 reference genome exclude non-3D7 VSAs, precluding a truly comprehensive analysis.^16^ Investigation of non-VSA genes may provide insight into the expression profiles of antigens with more conserved sequences and aid in the identification of additional parasite-encoded proteins that play a role in severe malaria.

This study took a transcriptome-wide approach to identify genes associated with severe malaria syndromes, including CM, SMA, and concurrent CM and SMA, compared to matched controls with uncomplicated malaria. Despite the exclusion of VSAs encoded by *var, rif,* and *stevor* genes, we included members of other multigene families, including genes encoded by *phist*, *fikk,* and *surf* genes, that have not been well-studied and may be comparatively more conserved. In CM cases, we hypothesized that other parasite genes encoding proteins that may interact with VSAs, assisting in processes like nutrient transport and cytoadherence, would exhibit differential expression over uncomplicated controls. Further, we anticipated that comparing CM cases to controls with a history of CM would yield a greater degree of differential expression because controlling for acquired immunity should result in better separation of cases from controls. For SMA, we hypothesized that few parasite genes are differentially expressed between cases and controls, as current evidence suggests that SMA is largely driven by host factors. We also anticipated that the comparison of parasites from children who presented with concurrent CM and SMA compared to uncomplicated malaria controls would yield the greatest number of differentially expressed genes, driven by specific parasite processes that heighten disease severity. A secondary aim of this study was to establish parasite expression profiles for malarial syndromes that have not been extensively characterized in previous literature—SMA and concurrent CM and SMA. Our overall goal across all of these comparisons was to identify parasite genes that may be dysregulated in specific severe malaria syndromes, noting that the directionality of expression may not be precise due to the reliance on the 3D7 reference genome, which may not include the exact members of a hypervariable gene family that are expressed in a clinical infection. Finally, we used a custom protein microarray to identify the development of immune responses to these antigens with severe illness, with a goal of validating expression findings and identifying potential foci of the development of protective natural immunity.

## Methods

### Study design and participants

Symptomatic Malian children from Bandiagara, Sikasso, and Bamako districts aged 6 months to 6 years old infected with *P. falciparum* were enrolled in this study, as recently described.^17^ Venous blood samples were obtained from children with consent from parents/guardians in compliance with the International Conference on Harmonization Good Clinical Practices, the Declaration of Helsinki, and Malian regulatory requirements. In the case of potentially illiterate participants, competent witnesses were used to ensure participants understood the contents of the informed consent document. Clinical trial protocols were approved by institutional review boards of the University of Sciences, Techniques and Technologies, Bamako, Mali (IRB00001983), and the University of Maryland, Baltimore (HP-00075140).

### Sample selection

The inclusion criteria to participate as a case in this study were: age between six months and ten years; confirmed infection with *P. falciparum* at the time of enrollment; residence in the three districts listed above; written informed consent from parents/guardians; and future availability for follow up. Cases of CM were defined using the standard WHO criteria—Blantyre Coma Score of less than or equal to two (scale ranges from zero to five with zero indicating unresponsiveness) with no other obvious signs of coma—and additional fundoscopic examination for the presence of malarial retinopathy.^17^ SMA cases had evidence of *P. falciparum* parasitemia on blood smear and a hemoglobin ≤5 g per deciliter.^17^ Two types of uncomplicated malaria controls were selected: those with and without a history of cerebral malaria, according to a validated survey.^18^ Uncomplicated malaria controls were symptomatic individuals who presented for treatment but did not require hospitalization. Controls were individually selected for each severe malaria subject. As a result, each severe malaria manifestation (CM, SMA, CM+ SMA) had individualized, distinct groups of controls selected specific to the severe malaria subtype being evaluated. All controls were enrolled and matched in real time to a severe malaria case based on similar age (within one year when possible), sex, ethnicity, and residence. Residence refers to the quartier, or neighborhood of residence, as already defined by existing regional boundaries.

Cases and controls were excluded from this study if they met the following criteria: simultaneous participation in an interventional clinical trial; chronic use of medication with known antimalarial activity in the previous seven days; history of chronic disease; or any condition that could jeopardize the safety or rights of a participant in the study that would render the participant unable to comply with the protocol. The sample size was determined by the availability of cases that met the study criteria and adequately matched controls. If a matched control was not able to be recruited for a severe malaria case, that case was omitted from the paired analysis.

### Sample collection

Venous blood samples from study participants were collected in PAXgene Blood RNA tubes to prevent destabilization of intracellular RNA during transport. Following blood collection, parasitemia was determined by slide reading. RNA was enriched using a whole transcriptome cDNA capture array.^19^ Capture probes were designed based on sequences of the 3D7 *P. falciparum* reference genome, as well as VSAs from a variety of genomes from strains collected worldwide.^19^ Only sequences captured by probes were sequenced, resulting in an enrichment of parasite over host cDNA in the final sequencing data.

Serum samples were also obtained from subjects at the time of presentation prior to initiation of treatment and in convalescence, defined as at least 21 days following presentation. Sera from 10 malaria-naïve North American blood donors served as negative controls.

### Data processing

Samples were sequenced using Illumina short-read sequencing technology after enrichment of parasite RNA. Using the Eukaryotic RNASeq pipeline at the Institute for Genome Sciences at the University of Maryland School of Medicine, sequence reads were mapped to the *P. falciparum* 3D7 reference genome (PlasmoDB v24) using HISAT2. Following alignment, reads were quantified using HTSeq. Given the polymorphic nature of genes within the *var, rif,* and *stevor* gene families,^11^ these VSA antigens were manually removed from count tables before subsequent analysis. For each sample, the proportion of each parasite life cycle stage was evaluated using deconvolution analysis within CIBERSORTx using B-mode for batch correction (under R Version 3.4.4), referring to a published RNA-seq Signature Matrix.^20^

### Gene expression analysis

For each of the three syndromes included in this study—CM, SMA, and concurrent CM and SMA – two sets of differential expression analysis were performed, comparing cases *versus* matched controls with and without a history of cerebral malaria. Differential gene expression analysis was performed using edgeR. Raw counts tables were filtered to remove lowly expressed genes (CPM ≤2 in at least half of the samples examined in each comparison). We applied the Trimmed Mean of M-Values (TMM) normalization within edgeR to account for systematic differences in library composition between samples.^21^ Two covariates were included in the differential expression model—the proportion of ring stage parasites (Tables S1–S5) and parasitemia (parasites/microliter) (Tables S6–S11). These covariates were selected based on their presumed influence on the outcome of differential expression analysis. Elevated parasitemia is correlated with increased severity of *P. falciparum* infection.^22^ Additionally, the proportion of ring stage parasites was selected because this is the parasite stage that predominates in the peripheral blood samples sequenced in this study.^23^ Differential expression analyses were performed using likelihood ratio tests (analogous to a paired *t*-test). An FDR threshold of ≤0.1 was applied to assign significance to differentially expressed genes.

Gene Ontology (GO) pathway analysis was conducted within the PANTHER interface using a Fischer’s exact test and FDR correction (GO Ontology database DOI: 10.5281/zenodo.7942786 Released 2023–05-10).^24^ GO pathways with an FDR ≤0.1 were considered significantly dysregulated. Differentially expressed genes were only compared to those genes that met the expression threshold for differential expression analysis (CPM ≥2 in at least half of the samples examined in each comparison).

### Protein microarrays

We amplified the open reading frames of 31 *Plasmodium falciparum* protein features from the 3D7 reference genome, followed by the cloning and printing of protein features on a chip, as described previously.^25^ The protein microarray primarily featured a set of differentially expressed genes identified in the transcriptome-wide analysis, including members of the FIKK, PHIST, and SURFIN families, as well as the mature parasite-infected erythrocyte surface antigen (MESA), ring-infected erythrocyte surface antigen (RESA), protein tyrosine phosphatase 5 (PTP5), ring-exported protein 3 (REX3), and HECT-like E3 ubiquitin ligase proteins. Microarrays were probed with sera to measure total IgG responses, and the arrays were scanned using a Perkin-Elmer (Waltham, MA) ScanArray Express HT microarray scanner followed by fluorescence quantification with the ScanArray Express Suite (PerkinElmer).

### Statistical analyses

Quantitative sample characteristic variables were presented as averages (minimum – maximum). Statistical differences for Parasitemia, Blantyre Coma Score, and Hemoglobin Level were assessed using the Wilcoxon signed-rank test at an alpha level of 0.05 (two-tailed). All statistical analyses were conducted independently for each group (*i.e.,* CM *versus* controls without a history of CM) in a paired fashion. Categorical sample characteristic variables are presented as categories with the corresponding frequency in parenthesis. P-values were calculated for all comparisons with a sample size greater than ten (Table S12). The Wilcoxon signed-rank test was used to evaluate the impact of ring *versus* trophozoite life-cycle stages on our results (alpha level 0.05) (Table S13, Figs. S1 and S2).

### Microarray analyses

Fluorescence intensity was defined as the raw signal intensity corrected by global median scaling for no-DNA negative control features. The resulting signal for each protein feature was called the median fluorescent intensity (MFI), which was used to assess seroreactivity, defined as the magnitude of microarray fluorescence intensity. Differences in reactivity between matched acute and convalescent sera as well as between severe malaria cases and matched controls were compared using Wilcoxon signed-rank tests. Presented p-values are two-sided without correction for multiple comparisons, using an alpha of 0.05 per previously described approaches for microarray analyses.^26,27^

### Role of the funding source

The funders of the study had no role in the study design, data collection, data analysis, data interpretation, or writing of this report.

## Findings

### Cerebral malaria sample characteristics

We sequenced parasite-enriched cDNA from whole blood samples collected from 36 Malian children infected with *P*. *falciparum*, including 14 CM cases and 22 matched, uncomplicated malaria controls (Tables 1, S6 and S7). The uncomplicated malaria group included matched controls with a history of CM (n = 8) and without a history of CM (n = 14) (Tables 1, S6 and S7). We conducted differential expression analyses comparing CM to each control group separately.

### Identification of differentially expressed *P. falciparum* genes in cerebral malaria

**Fig. 1.**
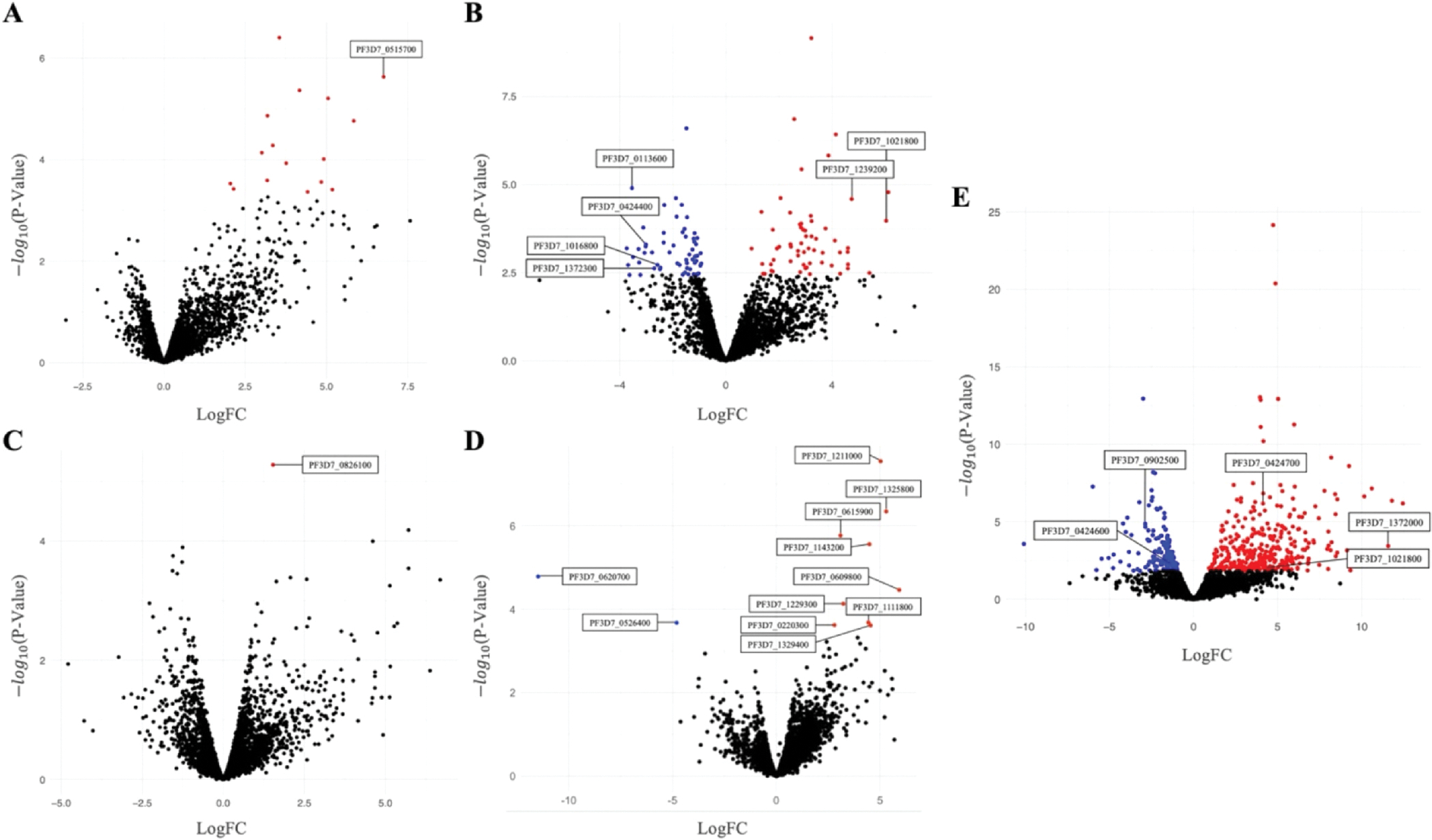
Volcano plot of differentially expressed *P. falciparum* genes in cases of cerebral malaria compared to controls without (A) and with (B) a history of cerebral malaria, cases of severe malarial anemia compared to controls without (C) and with (D) a history of cerebral malaria, and cases of concurrent cerebral malaria and severe malarial anemia compared to controls without (E) a history of cerebral malaria. Parasite genes are separated into those that showed significantly higher expression in cases of severe malaria (red), significantly higher expression in cases of uncomplicated malaria (blue), or no significant differences in expression across the two groups (black). Each point represents a single *P. falciparum* gene and is displayed according to the negative log_10_ of its corresponding p-value (y-axis) and log fold change (x-axis) following differential expression analysis.

There were 16 *P. falciparum* genes significantly differentially expressed between CM cases and matched controls without a history of CM (Table S14). Five of these 16 genes were proteins with unknown function. Differentially expressed genes with a known function included PF3D7_0515700 (FDR = 0.0038), encoding the glideosome-associated protein 40 (GAP40) (Fig. 1A; Table S14). The glideosome is a protein complex essential for parasite motility.^28^ GAP40 is an essential myosin light chain that interacts with the broader glideosome complex to assist in gliding motility and host cell invasion.^28^ Within this comparison, deconvolution analysis indicated that there was a significant difference between the proportion of ring stage and trophozoite parasites. However, analysis of expression levels of the differentially expressed genes in ring and trophozoite stages did not indicate differential life-cycle stage expression (Table S13).

When comparing CM cases to controls with a history of CM, 120 genes were differentially expressed—59 were upregulated in CM cases and 61 were downregulated (Fig. 1B; Table S15). Upregulated genes included the vaccine candidate schizont egress antigen-1 (SEA-1) (PF3D7_1021800; FDR = 0.018), a protein that plays an essential role in orchestrating the correct partitioning of DNA into merozoites (Fig. 1B; Table S15).^29^ Previous work demonstrated that antibodies to SEA-1 decrease parasite replication through a mechanism that arrests schizont rupture.^30^ In addition to SEA-1, an AP2-domain-containing transcription factor (PF3D7_1239200; FDR = 0.0077) was also upregulated in CM cases (Fig. 1B, Table S15). Transcription factors with AP2-domains play a variety of regulatory roles within the *P. falciparum* life cycle, and they may regulate gametocytogenesis initiation.^31^ Downregulated genes included multiple genes from the *phist* (PF3D7_1372300; FDR = 0.079 and PF3D7_1016800; FDR = 0.069) and *surf* (PF3D7_0113600; FDR = 0.0059 and PF3D7_0424400; FDR = 0.037) multigene families (Fig. 1B; Table S15). Members of the *phist* family play essential roles in host cell remodeling and assist in facilitating host-parasite cytoadherence through interactions with the acidic C-terminal segment of PfEMP1.^11,32^ *surf*s are expressed on the surfaces of merozoites and parasite-infected RBCs.^33^ SURFIN_4.2_ (PF3D7_0424400) has previously been shown to be co-transported to the erythrocyte surface with PfEMP1s and RIFINs, two of the principal VSA families implicated in CM pathogenesis.^33^

### Severe malarial anemia sample characteristics

We performed the same RNA sequencing and differential expression analysis on 10 samples from Malian children with SMA, nine uncomplicated malaria controls without a history of CM, and six uncomplicated malaria controls with a history of CM (Tables 2, S8 and S9).

### Identification of differentially expressed *P. falciparum* genes in severe malarial anemia cases

In the comparison of SMA cases to controls without a history of CM, there was a single differentially expressed gene—PF3D7_0826100, a putative gene encoding the HECT-like E3 ubiquitin ligase protein (FDR = 0.017) (Fig. 1C; Table S16).

Similarly, when comparing SMA cases to controls with a history of CM, we did not detect large-scale parasite gene expression differences. Eleven genes exhibited significant differential expression in this comparison—9 were upregulated in cases of severe malaria anemia and two were downregulated (Fig. 1D; Table S17). Three of these 11 genes had unknown functions.

### Concurrent cerebral malaria and severe malarial anemia sample characteristics

Differential expression analysis was performed on 20 samples from Malian children: eight children with concurrent cerebral malaria and severe malarial anemia, eight matched uncomplicated malaria controls without a history of CM, and four matched uncomplicated malaria controls with a history of CM (Tables 3, S10 and S11). Due to statistical power considerations and the small sample size in the group of controls with a history of CM, this latter control group was excluded from differential expression analysis.

### Identification of differentially expressed *P. falciparum* genes in cases with concurrent cerebral malaria and severe malarial anemia

Analysis of cases with concurrent CM and SMA *versus* controls yielded the most differentially expressed *P. falciparum* genes in this study. Comparing concurrent cases to controls without a history of cerebral malaria, we identified 524 significantly differentially expressed genes—342 of which were upregulated in CM+SMA cases, and 182 that were downregulated (Figs. 1E, S18). Similar to the comparison of CM cases *versus* controls with a history of CM, schizont egress antigen-1 (SEA-1; PF3D7_1021800) exhibited significant upregulation in concurrent cases (FDR = 0.077) (Fig. 1E; Table S18).

Several notable gene families that play a known role in severe malaria pathogenesis exhibited significant differential expression within this comparison. We identified seven AP2 domain transcription factors that displayed significant differential expression. Among *phist* family genes, 24 were differentially expressed (14 upregulated and 10 downregulated). This group includes several genes previously identified in Beninese cerebral malaria isolates that play a known role in PfEMP1 expression regulation—PF3D7_1372000 (PHISTa; FDR = 0.0078) and PF3D7_0424600 (PHISTb; FDR = 0.058) (Fig. 1E; Table S18).^16^ Further, five members of the *surf* gene family exhibited significant upregulation (SURFIN_1.3_, SURFIN_14.1_, SURFIN_8.1_, SURFIN_8.2_, and SURFIN_8.3_). Finally, we identified seven genes belonging to the serine/threonine protein kinase (*fikk*) family— five of which were upregulated, and two that were downregulated. Members of this gene family have previously been implicated in the modulation of erythrocytic remodeling during malaria disease through phosphorylation.^34^ Specifically, FIKK_4.2_ (PF3D7_0424700; FDR = 0.000061) plays a known role in the parasite-induced modification of erythrocytes (Fig. 1E; Table S18).^35^ Additionally, FIKK_9.6_ (PF3D7_0902500; FDR = 0.00066) was previously identified as differentially expressed in Beninese isolates and is believed to play a role in the regulation of PfEMP1 expression (Fig. 1E; Table S18).^16^

### Enrichment analysis of differentially expressed genes

Gene Ontology (biological processes, metabolic functions, and cellular components) enrichment analysis was performed on all differentially expressed gene subsets. Fisher’s exact tests and FDR correction were applied to determine statistical significance (FDR ≤0.1). No significant results were observed in comparisons of CM or SMA to controls with and without a history of CM. The lack of significance is likely driven by the small sample size of differentially expressed genes across all four of these sets of analysis.

**Fig. 2.**
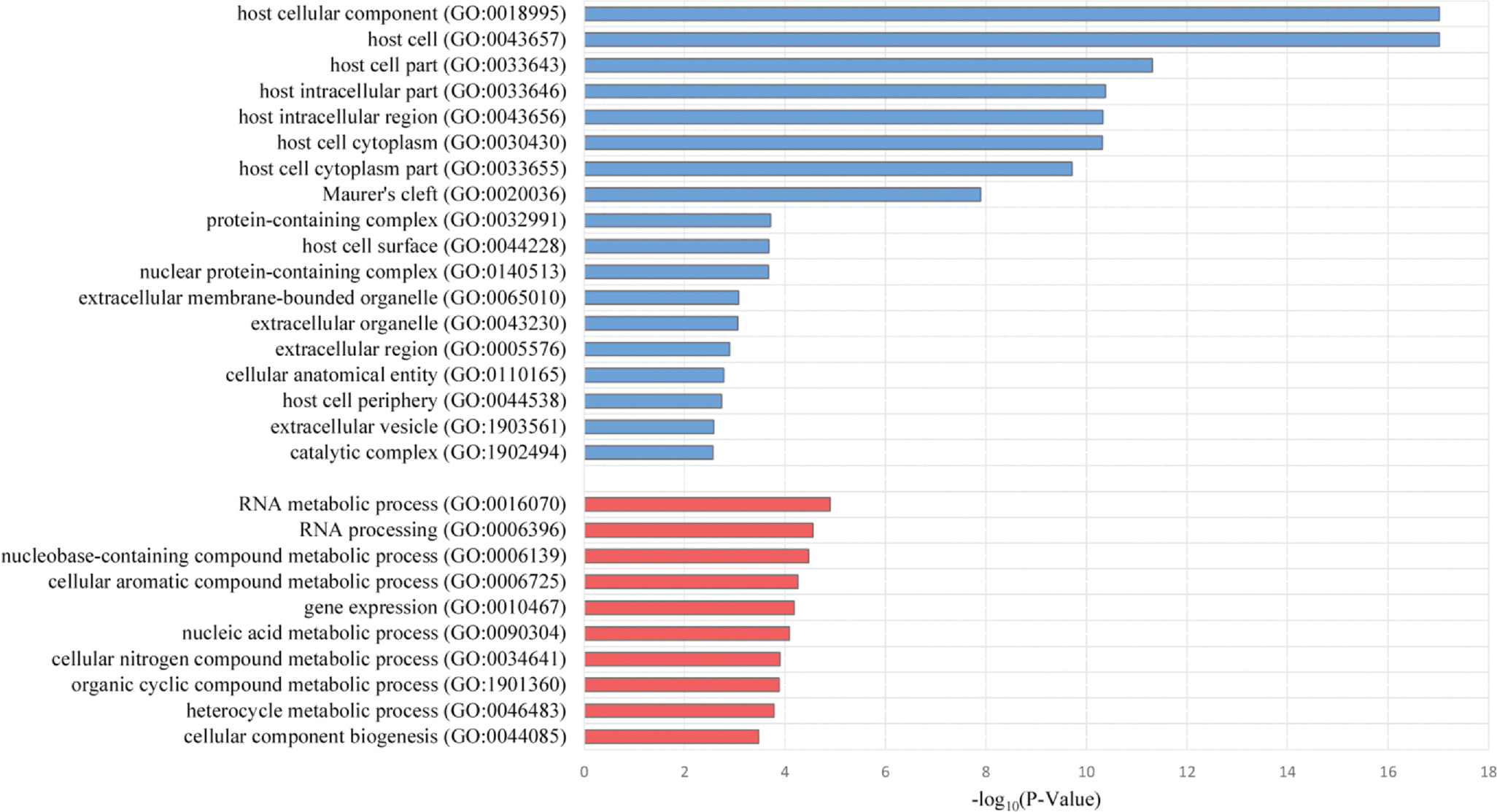
Gene Ontology (GO) cellular component (blue) and biological process (red) analysis of differentially expressed genes identified in the comparison of cases with concurrent CM and SMA to uncomplicated malaria controls. Each bar represents an individual *P. falciparum* GO Term and is displayed according to the negative log_10_ of its corresponding p-value (x-axis), reflecting differential expression between conditions of the component- or process-involved genes.

In contrast, 10 biological processes and 18 cellular components were significantly differentially regulated in the comparison of concurrent CM and SMA cases to controls without a history of CM (Fig. S4; Table S19). The majority of dysregulated biological processes were linked to RNA processing and related metabolic processes (Fig. 2; Table S19). The cellular components with the most significant enrichment were host cellular component (GO:0018995), host cell (GO:0043657), host cell part (GO:0033643), host cell cytoplasm (GO:0030430), host intracellular region (GO:0043656), host intracellular part (GO:0033646), and host cell cytoplasm part (GO:0033655) (Fig. 2; Table S19). Alteration in the expression of genes of such pathways associated with the host cell is, of course, plausible, given that *P. falciparum* invades and modifies host erythrocytes throughout the course of the disease. Additionally, genes associated with the secretory organelle Maurer’s cleft exhibited significant differences in gene expression (GO:0020036) (Fig. 2; Table S19). Maurer’s clefts are established by *P. falciparum* within host erythrocytes, where they play an essential role in nutrient transport, immune evasion processes, and host cell remodeling.^36^ Many proteins trafficked to the surface of parasitized erythrocytes (including PfEMP1) transiently localize to Maurer’s clefts.^36^

### Protein microarray analysis

**Fig. 3.**
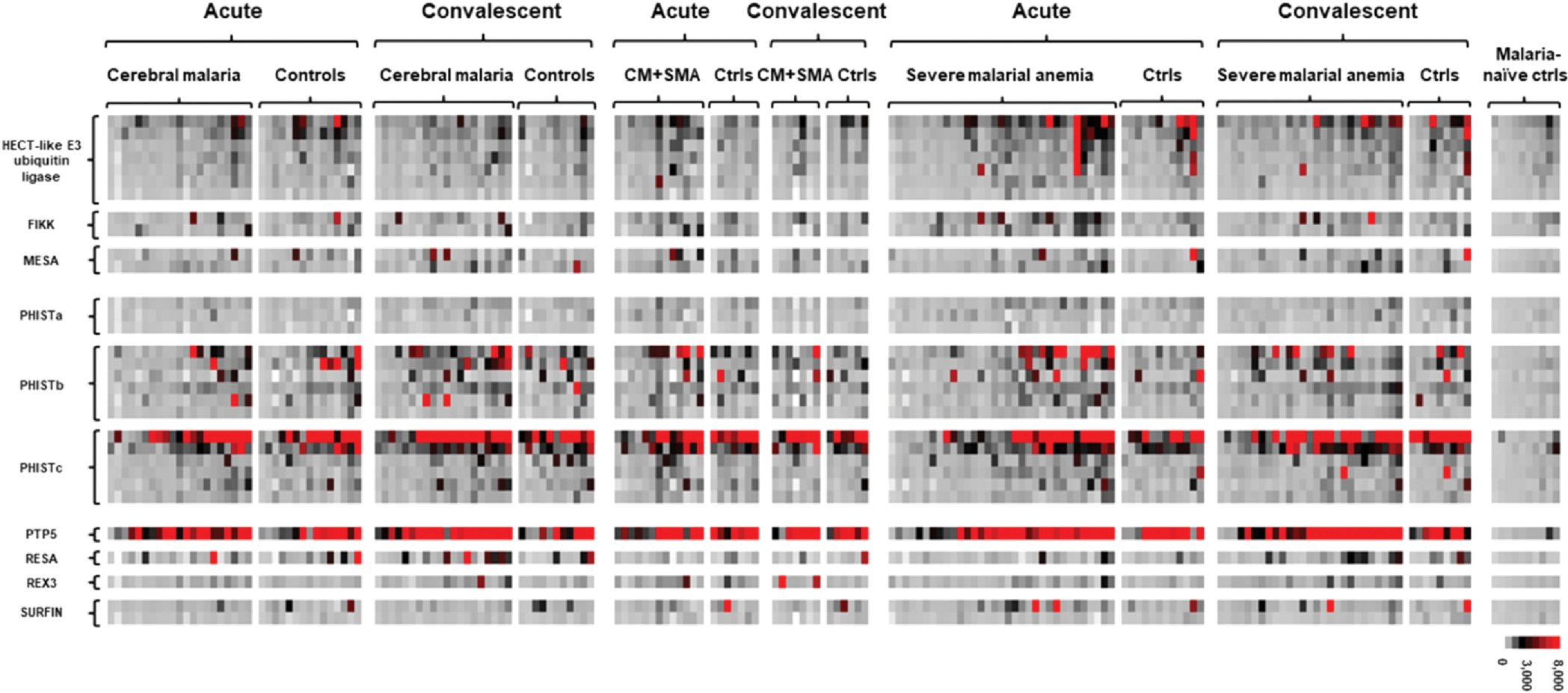
Heatmap of serologic responses to 31 protein fragments in children with severe malaria syndromes and matched controls with mild, uncomplicated malaria at the time of acute illness and in convalescence. The microarray included protein features from 2 FIKKs, 15 PHISTs, 2 SURFINs, MESA, RESA, PTP5, REX3, and HECT-like E3 ubiquitin ligase. The color indicates the magnitude of the response to each antigen ranging from gray (weak), to black (intermediate), and red (intense). Severe malaria syndromes included cerebral malaria, severe malarial anemia, and concurrent cerebral malaria and severe malarial anemia (“CM+SMA”). The serologic responses of 10 North American, malaria naïve controls were included as negative controls. We quantified the fluorescence intensity signifying the magnitude of antibody response of a serum sample to each microarray protein; the color represents the intensity as indicated in the key. Rows represent the signal intensities to each protein fragment, and columns represent the signal intensity for each serum sample. Individual serum samples are ordered by collection time point, with “convalescence” referring to collection at least 21 days from the time of acute illness, and by increasing mean fluorescence intensity within each subgroup.

We determined the serologic responses to 31 3D7 protein features at the time of acute illness as well as convalescence for children with severe malaria as well as matched controls with uncomplicated malaria (Fig. 3). We sought to identify immunologic lacunae, *i.e*., gaps in immune responses to malaria proteins between severe malaria cases and controls suggestive of vulnerability to disease. We did not find any significant differences in antibody responses between cerebral malaria cases and controls to malaria proteins at the time of acute illness (Fig. S8A). However, in convalescence, in comparison to acute illness presentation, children with cerebral malaria developed increased serologic responses to PHISTs (two PHISTbs and a PHISTa) and REX3 (P < 0.05; N=18 pairs; Fig. S8B). In contrast, the uncomplicated malaria controls for these cerebral malaria cases had an increased serologic response to only one protein, MESA, in convalescence (P < 0.05; N=10 pairs; Fig. S8C). Interestingly, at the time of acute illness, controls with a history of cerebral malaria had higher antibody responses to a SURFIN compared to cerebral malaria cases (P < 0.05; N=6 pairs; Fig. S8D). For these controls, antibody responses to the proteins featured on the microarray did not change significantly from the time of acute illness to convalescence (N=6 pairs).

We did not identify immunologic lacunae in comparing children with severe malarial anemia to matched uncomplicated controls at the time of illness (Fig. S9A). However, in convalescence, in comparison to presentation, children with severe malarial anemia had increased serologic responses to PHIST proteins (one PHISTb and two PHISTc proteins) and RESA (P < 0.05; N=26 pairs; Fig. S9B). In contrast, the uncomplicated malaria controls for these severe malarial anemia cases had an increased serologic response to only one protein, MESA, in convalescence (P < 0.05; N=9 pairs; Fig. S9C). Interestingly, at the time of acute illness, controls with a history of cerebral malaria had higher antibody responses to a PHISTb compared to severe malarial anemia cases (P < 0.05; N=6 pairs; Fig. S9D). There were no significant changes in antibody responses for these controls from the time of acute illness to convalescence (N=6 pairs).

Children with concurrent cerebral malaria and severe malarial anemia had several immunologic lacunae compared to matched controls, with lower antibody responses to a range of malaria proteins, including PHISTs, a SURFIN, MESA, and HECT-like E3 ubiquitin ligase (P < 0.05; N=7 pairs; (Fig. S10A). Unlike children with each individual severe malaria syndrome alone, children with concurrent cerebral malaria and severe malarial anemia did not develop increased serologic responses to any of the microarray proteins in convalescence (Fig. S10B). The uncomplicated malaria controls for cases with concurrent disease had an increased serologic response to one protein, HECT-like E3 ubiquitin ligase, in convalescence (P < 0.05; N=6 pairs; Fig. S10C).

## Discussion

In this study, we established *P. falciparum* gene expression and antibody profiles in Malian children with CM, SMA, or concurrent CM and SMA. While some *P. falciparum* transcriptome studies have focused on CM,^16^ the potential role of parasite gene expression in severe malaria syndromes involving severe malarial anemia has only recently begun to be investigated, even though SMA is the leading cause of death from malaria.^3,37^ Comparisons to matched controls with or without a history of CM provided insights into how carefully chosen controls can influence the differentially expressed genes identified.

This study builds upon previous work suggesting that *fikk, surf,* and *phist* gene families may play a crucial role in the pathogenesis of severe malaria syndromes.^16^ Our analyses implicated members of these multigene families in the three severe malaria syndromes examined, either as transcripts present in cases, in immunologic lacunae at the time of presentation, or in the subsequent development of antibody responses in convalescence, suggesting exposure to the antigen in acute illness. Notably, significantly altered expression of these gene families was identified not only in this study but also for *phist*s and *fikks* in a study examining CM isolates from Benin.^16^ These multigene families have been implicated in processes that escalate disease severity such as cytoadherence, erythrocytic remodeling, PfEMP1 interaction/regulation, and parasite invasion.^16,33–35^ In both this study and the Benin study, upregulation or dysregulation of certain members of the *phist* and a *fikk* gene families was associated with severe disease.^16^ As such findings suggest, upregulation alone is likely not the only path to severe pathology. It may be the case that downregulation of a particular parasite gene is pathological as opposed to protective and could potentially be linked to upregulation of another member of the same gene family or another family (including those not examined in this study that encode variant surface antigens such as *var*s, *rif*s, and *stevor*s).

Interestingly, we found differential expression of *phist* genes in severe disease involving cerebral malaria as well as the development of antibody responses to PHISTs in convalescence, suggesting that these proteins are exposed to the immune system at the time of illness. Immune responses to PHISTs could contribute to the development of protective natural immunity. Little is known about PHIST antigen diversity. Further investigation of PHIST antibody responses could provide insight into PHIST antibody cross-reactivity and associations with protection against severe disease. If PHIST antigen diversity is more limited than that in PfEMP1s, RIFINs, and STEVORs, they may be appealing targets for therapeutics and vaccine development.

We have identified several multigene families with significant gene dysregulation associated with severe malarial syndromes. An important next step is the analysis of these gene families with more specific methods that sequence the actual genes expressed in these infections, not relying on the 3D7 reference genome as a stand-in. Methods such as *de novo* assembly of the actual transcripts in an infection can be used to definitively characterize expression of particular members of gene families or specific variants of identified genes.^19^ The level of expression of these genes would be the most accurate way to measure directionality of gene expression for hypervariable gene families, albeit one that may be daunting in the complexity of the analysis.

Malian children with concurrent CM and SMA are at an especially high risk of death from *P. falciparum* malaria,^6^ as they have multiple symptoms associated with an increased risk of mortality, including impaired consciousness and severe anemia. When examining concurrent CM and SMA cases, we identified several differentially expressed genes previously implicated in a variety of processes that could influence disease severity, including erythrocyte remodeling,^35^ VSA regulation/interaction,^16^ facilitation of parasite-host interaction,^32^ and effective DNA replication.^29^ In convalescence, children with concurrent CM and SMA did not have an increased antibody response to parasite proteins, in contrast to children with CM or SMA alone. This failure to mount appropriate antibody responses in convalescence could signal immune dysregulation unique to this severe malaria syndrome and contribute to increased mortality.

Interestingly, of the five comparisons of severe malaria syndromes to uncomplicated malaria controls, the comparison of concurrent CM and SMA to controls without a history of CM yielded the highest number of differentially expressed genes. The factors that drive the progression of uncomplicated malaria to severe and life-threatening manifestations of malaria remain unclear. This progression to severe disease may be triggered by parasite expression, host vulnerability, or a combination of both. The large subset of differentially expressed *P. falciparum* genes in children with concurrent CM and SMA relative to controls — the highest degree of gene dysregulation across any of the expression comparisons in this study — suggests that parasite antigens besides variant surface antigens may play a critical role in the progression to particular severe malaria syndromes. While we have identified multiple genes associated with concurrent disease, it remains unclear whether this parasite expression profile identifies parasite antigens driving virulence or a response to the host’s condition. In any case, our results suggest that a distinct parasite gene expression profile is associated with concurrent CM and SMA.

SMA results from the destruction of erythrocytes and reduced erythrocyte production in the bone marrow, but the molecular mechanisms underlying these processes remain unknown.^38^ Both host (inflammatory response and immune activity) and parasite (erythrocyte interaction with parasite ligands/antigens) processes have been suggested as potential factors contributing to SMA.^5^ In this study, we report low levels of differential parasite gene expression for SMA cases compared to both controls with and without a history of CM. Our findings suggest the need to search more broadly for explanations for the etiology of SMA, including measuring parasite variant surface antigen expression, specifically, and examining the host response and inflammatory processes.

Heightened host and immune-specific responses are not the only plausible explanation for the lack of differential expression observed within SMA cases in this study. It is possible that expression of VSAs from the *var, rif,* or *stevor* gene families plays a role in SMA. Reads mapping to these three VSA antigen families were excluded before differential expression analyses because other high sequence variability and polymorphic nature of VSAs make the mapping to a reference genome difficult and unreliable.^11^ As a result, VSA expression profiles in SMA cases compared to uncomplicated controls were not quantified. Our group has previously found that Malian children with SMA lack antibody responses to a subset of variant surface antigens, including PfEMP1s and a STEVOR, suggesting that VSAs could play a role in SMA pathogenesis.^26^ In addition, the weak differential expression signal associated with SMA could be the result of small sample sizes restricting our ability to determine differences between SMA cases and controls. Future severe malarial anemia investigations should address variant surface antigen expression and sample size. This would provide the best determination of whether severe malarial anemia is fundamentally a host-driven response to infection without a significant contribution from parasite-derived factors. Further, additional omics modalities may provide key insights into the parasite proteins associated with SMA, as was performed in a recent proteomics study of severe malaria syndromes occurring in Beninese children.^39^

In the present study, we used two different sets of controls – those with and without a history of CM. The time that it takes for children to acquire immunity to severe malaria remains unclear. One mathematical model suggested that immunity to non-cerebral severe malaria may be acquired after one or two *P. falciparum* infections,^40^ and there is some evidence to suggest that immunity to cerebral malaria may be similarly acquired.^41^ Further, an alternate model has suggested that the acquisition of immunity to severe malaria is a more gradual process.^42^ For this study, we assumed that children with a history of CM are less likely to progress from uncomplicated malaria to CM due to acquired immunity. Conversely, we assumed that children without a history of CM could theoretically progress to CM if left untreated. Thus, in the comparison of CM cases to uncomplicated malaria controls with a history of CM, we attempted to control for progression to CM, as this group is presumed to have acquired clinical immunity to CM. Microarray analyses indicated higher antibody responses to a particular SURFIN distinguished controls with a history of cerebral malaria from cerebral malaria cases, implicating this family in cerebral malaria pathogenesis and potential protection against this form of severe malaria.

Notably, when examining both differential expression analyses of CM cases to controls with and without a history of CM, the former comparison indicated a more diverse parasite expression profile (120 *versus* 16 differentially expressed genes). The lack of differential parasite expression in the comparison to controls without a history of CM suggests that gene expression patterns in parasites sequenced from this group may not be significantly different than those sequenced from CM cases. Such controls may progress to severe malarial disease such as cerebral malaria without appropriate, timely treatment. The larger set of differentially expressed genes observed in the comparison of CM cases to controls with a history of CM implies that, by controlling for a history of the disease, cases are more effectively separated from controls, thereby enabling better isolation of the role of parasite expression in the pathogenesis of CM. The results of this study have implications for future case-control studies and suggest that, in addition to carefully matching controls on sociodemographic variables (residence, sex, age, *etc*.), severe malaria history should be considered to effectively create compositionally distinct control groups.

Our findings should inform the development of future vaccines and therapeutic targets, as genes that are significantly differentially regulated in severe malaria may contribute to disease pathogenesis. An example of such a gene is the vaccine candidate schizont egress antigen-1, which was found to be significantly upregulated in cerebral malaria comparisons within this study. Previous work has demonstrated that Tanzanian children with antibodies to recombinant PfSEA-1 did not experience severe malaria, and Kenyans with PfSEA-1 antibodies had significantly lower parasite densities than individuals without antibodies.^30^ When considered alongside other studies, our findings highlight the importance of a transcriptome-wide approach to differential expression analysis and suggest that several gene families warrant further investigation.

It is possible that the transcription profiles that we have identified may reflect not only parasite genes involved in pathogenesis, but also responses to the host’s disease state itself. Future investigations should include transcriptional profiling of children with infections that span the full spectrum from mild, uncomplicated illness to specific severe disease phenotypes. Transcriptional profiling of children with a Blantyre Coma Score lower than 5 but not below 3 could provide insights into parasite transcription specifically involved in the development of cerebral malaria. Likewise, the parasite transcriptional profiles of children with anemia that does not meet the 5 g/dL threshold for severe malarial anemia may provide insights into parasite genes implicated in the development of this disease state. Animal models of severe disease may also provide insights into parasite genes specific to disease pathogenesis, but this has thus far been limited given the challenges of accurately reproducing *P. falciparum* severe disease.

This study had several limitations. Small sample sizes across comparisons (particularly SMA and CM+SMA) limit the statistical power of subsequent analyses and generalizability of findings. Further, due to the exclusion of established VSAs from the *var, rif,* and *stevor* gene families before differential expression analysis, their roles in the pathogenesis of severe malaria syndromes were not evaluated. The lack of inclusion of these established VSAs in the present study limits our scope of understanding of severe malaria pathogenesis. Future studies will apply *de novo* assembly techniques to quantify the expression of these VSAs in this severe malaria case-control study. Moreover, the present study applied an FDR threshold of 0.1 (as opposed to 0.05) to increase the sensitivity of analyses, as we have done in other work examining *P. falciparum* expression.^43^ Further, when attempting to control for acquired immunity, we only selected controls on the basis of their history of CM. It is possible that this control group was not as relevant to severe malaria comparisons outside of CM such as SMA and concurrent CM and SMA. Future studies using controls with a history of severe malaria should attempt to match the history of the disease to the manifestation being examined (*e.g*., comparison of SMA cases to controls with a history of SMA). As previously discussed, the time it takes for children to acquire immunity to severe malaria is uncertain. Thus, it is possible that uncomplicated malaria controls with a history of cerebral malaria may not have sufficient protective immunity to CM. Additional studies should assess antibody responses in this population to malaria antigens associated with cerebral malaria. Moreover, deconvolution analysis revealed parasite life-cycle differences in one cerebral malaria-control comparison. While there was no difference in expression levels of genes of interest between these life-cycle stages, single-cell studies would provide the most precise assessment of differential gene expression between lifecycle stages. Data from such studies are not yet available. Moreover, future investigations could use single-cell approaches and periodic sampling in a similarly designed case-control study to establish whether (i) parasite profiles are reacting to or driving severity of infection and (ii) biological differences between parasites contribute to each disease manifestation. The Custom Capture Array (CCA) RNA Enrichment approach could produce a bias towards 3D7-based gene variant expression as its probes for non-*var* sequences are based on this reference genome exclusively; however, the CCA demonstrated no evidence of such bias in a previous analysis.^19^ Microarray analyses focused on 3D7 variants of malaria proteins, including those encoded by the *phist*, *fikk*, and *surf* multigene families. The actual antigenic variants present in these infections may differ substantially from their closest 3D7 counterparts. As such, the immune responses elicited to members of these families on the protein microarray may be inexact representations of the actual immune responses to the antigenic variants in the infection. This 3D7 microarray provides a first step in approximating immune responses to these malaria proteins, but future microarrays should include assaying antibody responses to the antigenic variants present in severe malaria infections if possible.

Addressing the tremendous burden of malaria requires an in-depth understanding of the role of *P. falciparum* across severe malaria syndromes. In this study, we identified parasite gene expression and antibody profiles for three manifestations of severe malaria—CM, SMA, and concurrent CM and SMA. We established novel parasite gene expression associations specific to CM and concurrent disease and highlight the need for further investigation of the contributions of the *phist*, *fikk*, and *surf* multigene families in pathogenesis and immunity. By comparing cases to control groups with and without a history of CM, we also demonstrate how the selection of controls can impact parasite expression profiles to guide the development of future studies.

## Supplementary Material

1

Appendix A. Supporting information

Supplementary data associated with this article can be found in the online version at doi:10.1016/j.jinf.2025.106655.

## Figures and Tables

**Table 1:** Characteristics of CM case-control study subjects (n = 36).

		CM Cases (n = 14)	Controls w/o CM History (n = 14)	Controls w/ CM History (n = 8)

Parasitemia (parasites /microliter)		141,537 ± 43,777	29,857 ± 12,286	37,838 ± 19,980
		(1200–450,600)	(1425–165,000)*	(1050–158,400)
Blantyre Coma Score	1	3 (21.4%)	0 (0.0%)	0 (0.0%)
	2	11 (78.6%)	0 (0.0%)	0 (0.0%)
	3	0 (0.0%)	0 (0.0%)	0 (0.0%)
	4	0 (0.0%)	0 (0.0%)	0 (0.0%)
	5	0 (0.0%)	5: 14 (100%)*	5: 8 (100%)*
Hemoglobin Level (grams/deciliter)		7.9 ± 0.5 (5.3–11.6)	8.5 ± 0.5 (5.5–11.6)	9.6 ± 0.5 (7.8–11.4)
Male (%)		8 (57.1%)	8 (57.1%)	7 (87.5%)
Blood Type	A	3 (21.4%)	5 (35.7%)	2 (25.0%)
	B	2 (14.3%)	2 (14.3%)	1 (12.5%)
	AB	2 (14.3%)	0 (0.0%)	1 (12.5%)
	O	7 (50.0%)	7 (50.0%)	4 (50.0%)
Age (years)	0–1	1 (7.1%)	0 (0.0%)	0 (0.0%)
	1–2	3 (21.4%)	4 (28.6%)	0 (0.0%)
	2–3	1 (7.1%)	1(7.1%)	1 (12.5%)
	3–4	5 (35.7%)	3 (21.4%)	3 (37.5%)
	4–5	2 (14.3%)	6 (42.9%)	3 (37.5%)
	5–6	2 (14.3%)	0 (0.0%)	1 (12.5%)
Ethnicity	Dogon	12 (85.7%)	12 (85.7%)	8 (100%)
	Bambara	2 (14.3%)	2 (14.3%)	0 (0.0%)
Collection Site	Bandiagara	12 (85.7%)	12 (85.7%)	8 (100%)
	Bamako	2 (14.3%)	2 (14.3%)	0 (0.0%)

CM, cerebral malaria. Quantitative values are presented as an average and standard error with the corresponding minimum and maximum. Categorical variables are presented as raw counts of the number of subjects falling into each group followed by the corresponding percentage. Values with a “*” indicate statistically significant differences at an alpha level of 0.05 between cases compared to the matched control groups.

**Table 2:** Characteristics of SMA case-control study subjects (n = 26).

		SMA Cases (n = 10)	Controls w/o CM History (n = 9)	Controls w/ CM History (n = 6)

Parasitemia (parasites /microliter)		64,590 ± 18,798	30,300 ± 13,239	60,263 ± 35,416
		(3450–181,500)	(1050–119,400)	(150–192,000)
Blantyre Coma Score	1	0 (0.0%)	0 (0.0%)	0 (0.0%)
	2	0 (0.0%)	0 (0.0%)	0 (0.0%)
	3	3 (30.0%)	0 (0.0%)	0 (0.0%)
	4	2 (20.0%)	0 (0.0%)	0 (0.0%)
	5	5 (50.0%)	9 (100%)^+^	5: 6 (100%)^+^
Hemoglobin Level (grams/deciliter)		3.3 ± 0.3 (2.3–5.0)	8.8 ± 0.6 (6.2–11.5)*	8.7 ± 0.8 (5.9–11.6)*
Male (%)		2 (20.0%)	1 (11.1%)	2 (33.3%)
Blood Type	A	0 (0.0%)	2 (22.2%)	1 (16.7%)
	B	7 (70.0%)	2 (22.2%)	3 (50.0%)
	AB	1 (10.0%)	1 (11.1%)	0 (0.0%)
	O	2 (20.0%	4 (44.4%)	2 (33.3%)
Age (years)	0–1	0 (0.0%)	0 (0.0%)	0 (0.0%)
	1–2	2 (20.0%)	2 (22.2%)	1 (16.7%)
	2–3	1 (10.0%	0 (0.0%)	0 (0.0%)
	3–4	6 (60.0%)	6 (66.7%)	4 (66.7%)
	4–5	0 (0.0%)	0 (0.0%)	0 (0.0%)
	5–6	1 (10.0%)	1 (11.1%)	1 (16.7%)
Ethnicity	Bambara	1 (10.0%)	0 (0.0%)	1 (16.7%)
	Dogon	8 (80.0%)	8 (88.9%)	5 (83.3%)
	Peuhl	1 (10.0%)	1 (11.1%)	0 (0.0%)
Collection Site	Bandiagara	8 (80.0%)	8 (88.9%)	1 (16.7%)
	Bamako	2 (20.0%)	1 (11.1%)	5 (83.3%)

SMA, Severe Malarial Anemia; Con w/o History, control without a history of cerebral malaria; Con w/ History, control with a history of cerebral malaria. Quantitative values are presented as an average and standard error with the corresponding minimum and maximum. Categorical variables are presented as raw counts of the number of subjects falling into each group followed by the corresponding percentage. Values with a “*” indicate statistically significant differences between cases compared to the matched control groups. Values with a “+” indicate that the sample size was not large enough to perform a Wilcoxon signed-rank test at an alpha level of 0.05.

**Table 3:** Characteristics of concurrent CM and SMA case-control study subjects (n = 20).

		CM+SMA Cases (n = 8)	Con w/o History (n = 8)	Con w/ History (n = 4)

Parasitemia (parasites /microliter)		64,125 ± 33,870	9928 ± 4037	3963 ± 3005
		(525–242,400)	(375–36,000)	(325–12,950)^+^
Blantyre Coma Score	1	4 (50.0%)	0 (0.0%)	0 (0.0%)
	2	4 (50.0%)	0 (0.0%)	0 (0.0%)
	3	0 (0.0%)	0 (0.0%)	0 (0.0%)
	4	0 (0.0%)	0 (0.0%)	0 (0.0%)
	5	0 (0.0%)	5: 8 (100%)*	5: 4 (100.0%)^+^
Hemoglobin Level (grams/deciliter)		3.3 ± 0.3 (2.2–4.6)	8.1 ± 0.9 (5.0–11.8)*	8.8 ± 1.1 (7.0–12.1)^+^
Male (%)		6 (75.0%)	6 (75.0%)	2 (50.0%)
Blood Type	A	0 (0.0%)	2 (25.0%)	0 (0.0%)
	B	5 (62.5%)	3 (37.5%)	2 (50.0%)
	AB	1 (12.5%)	0 (0.0%)	0 (0.0%)
	O	2 (25.0%)	3 (37.5%)	2 (50.0%)
Age (years)	0–1	0 (0.0%)	0 (0.0%)	0 (0.0%)
	1–2	1 (12.5%)	1 (12.5%)	0 (0.0%)
	2–3	1 (12.5%)	1 (12.5%)	0 (0.0%)
	3–4	3 (37.5%)	2 (25.0%)	2 (50.0%)
	4–5	1 (12.5%)	3 (37.5%)	1 (25.0%)
	5–6	2 (25.0%)	1 (12.5%)	1 (25.0%)
Ethnicity	Bambara	1 (12.5%)	1 (12.5%)	0 (0.0%)
	Dogon	6 (75.0%)	6 (75.0%)	4 (100%)
	Sarakolé	1 (12.5%)	0 (0.0%)	0 (0.0%)
	Malinke	0 (0.0%)	1 (12.5%)	0 (0.0%)
Collection Site	Bandiagara	6 (75.0%)	6 (75.0%)	4 (100%)
	Bamako	2 (25.0%)	2 (25.0%)	0 (0.0%)

SMA, severe malarial anemia; CM+SMA, concurrent cerebral malaria, and severe malarial anemia; Con w/o History, control without a history of cerebral malaria; Con w/ History, control with a history of cerebral malaria. Quantitative values are presented as an average and standard error with the corresponding minimum and maximum. Categorical variables are presented as raw counts of the number of subjects falling into each group followed by the corresponding percentage. Values with a “*” indicate statistically significant differences between cases compared to the matched control groups. Values with a “+” indicate that the sample size was not large enough to perform a Wilcoxon signed-rank test at an alpha level of 0.05.

## Data Availability

All parasite sequence data will be released to a public database upon publication.
