# Supplementary material for: Plasmodium falciparum gene expression signatures and antibody profiling implicate the fikk, phist, and surf multigene families in severe malaria syndromes
Source: J Infect. Author manuscript; Available in PMC 2026 Jan 29. (PMC12854251; doi:10.1016/j.jinf.2025.106655)
Supplement: 1 [file NIHMS2128219-supplement-1.docx]

**TABLE OF CONTENTS**

Supplementary Methods…………...…………………………………………………………...……………………...3

Supplementary Figures………………………………………………………………………………………………...3

- **Figure S1.** Box plot comparison of the proportion of ring stage parasites across all five comparisons of severe malarial syndromes *versus* uncomplicated malaria controls.
- **Figure S2.** Box plot comparison of the proportion of trophozoites across all five comparisons of severe malarial syndromes *versus* uncomplicated malaria controls.
- **Figure S3.** Principal component analysis demonstrating separation of cases of cerebral malaria and uncomplicated malaria controls without a history of cerebral malaria along the first two principal components.
- **Figure S4.** Principal component analysis demonstrating separation of cases of cerebral malaria and uncomplicated malaria controls with a history of cerebral malaria along the first two principal components.
- **Figure S5**. Principal component analysis demonstrating separation of cases of severe malarial anemia and uncomplicated malaria controls without a history of cerebral malaria along the first two principal components.
- **Figure S6.** Principal component analysis demonstrating separation of cases of severe malarial anemia and uncomplicated malaria controls with a history of cerebral malaria along the first two principal components before (A) and after (B) removal of UM2.
- **Figure S7.** Principal component analysis demonstrating separation of cases of concurrent cerebral malaria and severe malarial anemia and uncomplicated malaria controls without a history of cerebral malaria.
- **Figure S8**. Serologic response comparisons on a custom protein microarray for (A) severe malarial anemia subjects versus matched uncomplicated malaria controls at the time of acute illness, (B) severe malarial anemia subjects at the time of acute illness versus in convalescence, and (C) uncomplicated malaria controls at the time of acute illness versus in convalescence.
- **Figure S9.** Serologic response comparisons on a custom protein microarray for (A) severe malarial anemia subjects versus matched uncomplicated malaria controls at the time of acute illness, (B) severe malarial anemia subjects at the time of acute illness versus in convalescence, and (C) uncomplicated malaria controls at the time of acute illness versus in convalescence.
- **Figure S10.** Serologic response comparisons on a custom protein microarray for (A) subjects with concurrent cerebral malaria and severe malarial anemia versus matched uncomplicated malaria controls at the time of acute illness, (B) subjects with concurrent cerebral malaria and severe malarial anemia at the time of acute illness versus in convalescence, and (C) uncomplicated malaria controls at the time of acute illness versus in convalescence.

Supplementary Tables…………………………………………………………………………………………….......10

- **Table S1**. CIBERSORTx *P. falciparum* life-cycle stage proportions in the comparison of CM cases to uncomplicated malaria controls without a history of CM.
- **Table S2.** CIBERSORTx *P. falciparum* life-cycle stage proportions in the comparison of CM cases to uncomplicated malaria controls with a history of CM.
- **Table S3.** CIBERSORTx *P. falciparum* life-cycle stage proportions in the comparison of SMA cases to uncomplicated malaria controls without a history of CM.
- **Table S4.** CIBERSORTx *P. falciparum* life-cycle stage proportions in the comparison of SMA cases to uncomplicated malaria controls with a history of CM.
- **Table S5.** CIBERSORTx *P. falciparum* life-cycle stage proportions in the comparison of concurrent CM and SMA cases to uncomplicated malaria controls without a history of CM.
- **Table S6.** Comprehensive sample characteristics for the comparison of CM cases to uncomplicated malaria controls without a history of CM.
- **Table S7.** Comprehensive sample characteristics for the comparison of CM cases to uncomplicated malaria controls with a history of CM.
- **Table S8.** Comprehensive sample characteristics for the comparison of SMA cases to uncomplicated malaria controls without a history of CM.
- **Table S9.** Comprehensive sample characteristics for the comparison of SMA cases to uncomplicated malaria controls with a history of CM.
- **Table S10.** Comprehensive sample characteristics for the comparison of concurrent CM and SMA cases to uncomplicated malaria controls without a history of CM.
- **Table S11.** Comprehensive sample characteristics for the comparison of concurrent CM and SMA cases to uncomplicated malaria controls with a history of CM.
- **Table S12.** Wilcoxon signed rank test P-value results for quantitative demographic sample variables.
- **Table S13.** 6-hour (ring-stage) and 24-hour (trophozoite) smoothed 3D7 expression values for differentially expressed genes in the comparison of CM cases to uncomplicated malaria controls without a history of CM.
- **Table S14.** 16 significantly differentially expressed genes in the comparison of CM cases to uncomplicated malaria controls without a history of CM.
- **Table S15.** 120 differentially expressed genes in the comparison of CM cases to uncomplicated malaria controls without a history of CM.
- **Table S16.** Singular significantly differentially expressed gene in the comparison of SMA cases to uncomplicated malaria controls without a history of CM.
- **Table S17.** Eleven significantly differentially expressed genes in the comparison of SMA cases to uncomplicated malaria controls with a history of CM.
- **Table S18.** 524 significantly differentially expressed genes in the comparison of concurrent CM and SMA to uncomplicated malaria controls without a history of CM.
- **Table S19.** Significant Gene Ontology (GO) biological processes and cellular component results for concurrently infected cases compared to controls without a history of CM.

Additional References………………………………………………………………………………………….........35

**SUPPLEMENTARY METHODS**

*Statistical Analysis*

Evaluation of ring stage and trophozoite expression on differential expression analysis results for cerebral malaria cases *versus* controls without a history of cerebral malaria:

The comparison of cerebral malaria cases *versus* controls without a history of cerebral malaria was the only comparison where the proportions of CIBERSORTx ring stage parasites and trophozoite significantly differed (P=0.041 and 0.019, respectively). To evaluate if this difference impacted our differential expression analysis findings, we compared *P. falciparum* 3D7 smoothed expression values for 13/16 differentially expressed genes obtained from a previous transcriptome study that evaluated *P. falciparum* expression over a 48-hour period to establish intraerythrocytic life cycle expression.^1^ The 6-hour time point was used as a proxy for ring stage expression and the 24-hour timepoint was used as a proxy for the trophozoite expression. Smoothed 3D7 expression values were compared using a Wilcoxon signed-rank test at an alpha level of 0.05.

*Principal Component Analysis*

Principal component analysis (PCA) was performed prior to differential expression analyses for all comparisons examined within this study. Analysis was conducted within R-studio using the ggplot2 package for visualization. The goal of this analysis was to identify outliers prior to executing differential expression analysis and provide an overview of the heterogeneity within each comparison. Except for the comparison of cases of severe malarial anemia to uncomplicated malaria controls with a history of cerebral malaria, PCA analysis did not indicate global differences across comparisons, nor did it identify any significant outliers. Within the comparison of cases of severe malarial anemia to uncomplicated malaria controls with a history of cerebral malaria, we identified a singular outlier (Figure S6A) within the group of uncomplicated malaria controls. This sample along with its paired severe malarial anemia case were removed prior to differential expression analysis.

**SUPPLEMENTARY FIGURES**

**Figure S1.** Box plot comparison of the proportion of ring stage parasites across all five comparisons of severe malarial syndromes *versus* uncomplicated malaria controls. Each comparison (x-axis) is separated into the proportion of ring stage parasites (y-axis) identified in cases of severe malaria (black)—cerebral malaria, severe malarial anemia, and concurrent syndrome—and uncomplicated malaria controls with (CWH) and without (CNH) a history of cerebral malaria (white).

* P-Value = 0.041

**Figure S2.** Box plot comparison of the proportion of trophozoites across all five comparisons of severe malarial syndromes *versus* uncomplicated malaria controls. Each comparison (x-axis) is separated into the proportion of trophozoites (y-axis) identified in cases of severe malaria (black)—cerebral malaria, severe malarial anemia, and concurrent syndrome—and uncomplicated malaria controls with (CWH) and without (CNH) a history of cerebral malaria (white).

* P-Value = 0.019

**
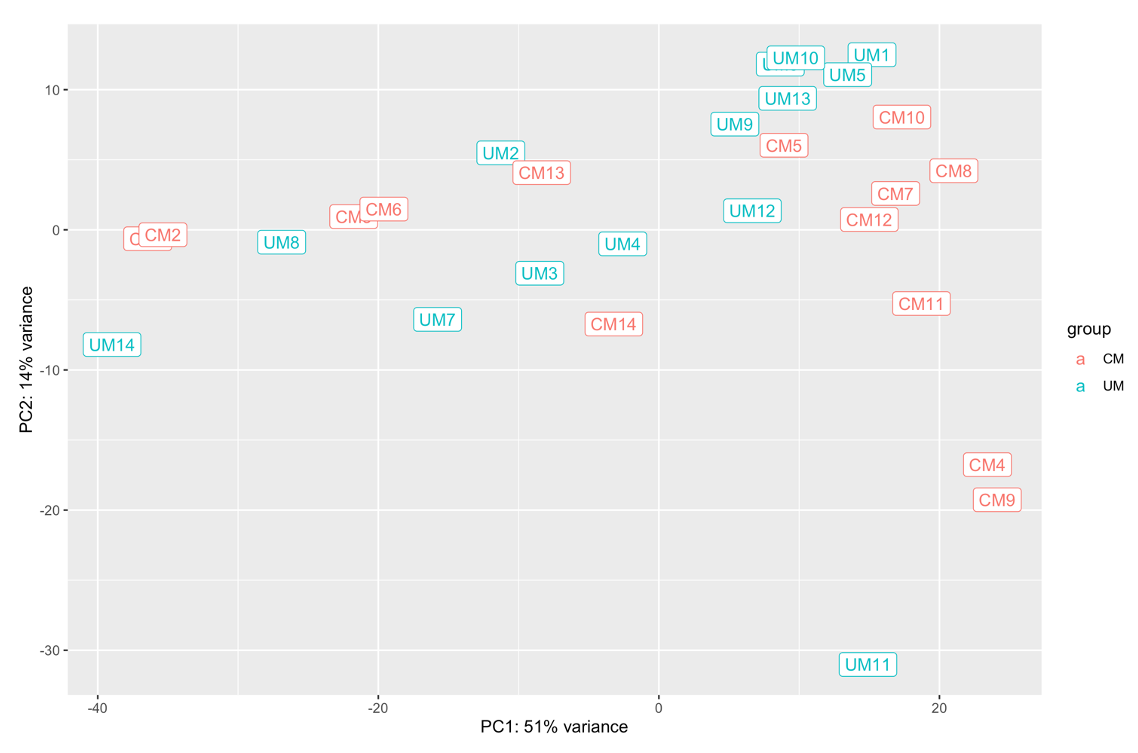
**

**Figure S3.** Principal component analysis demonstrating separation of cases of cerebral malaria and uncomplicated malaria controls without a history of cerebral malaria along the first two principal components.

**
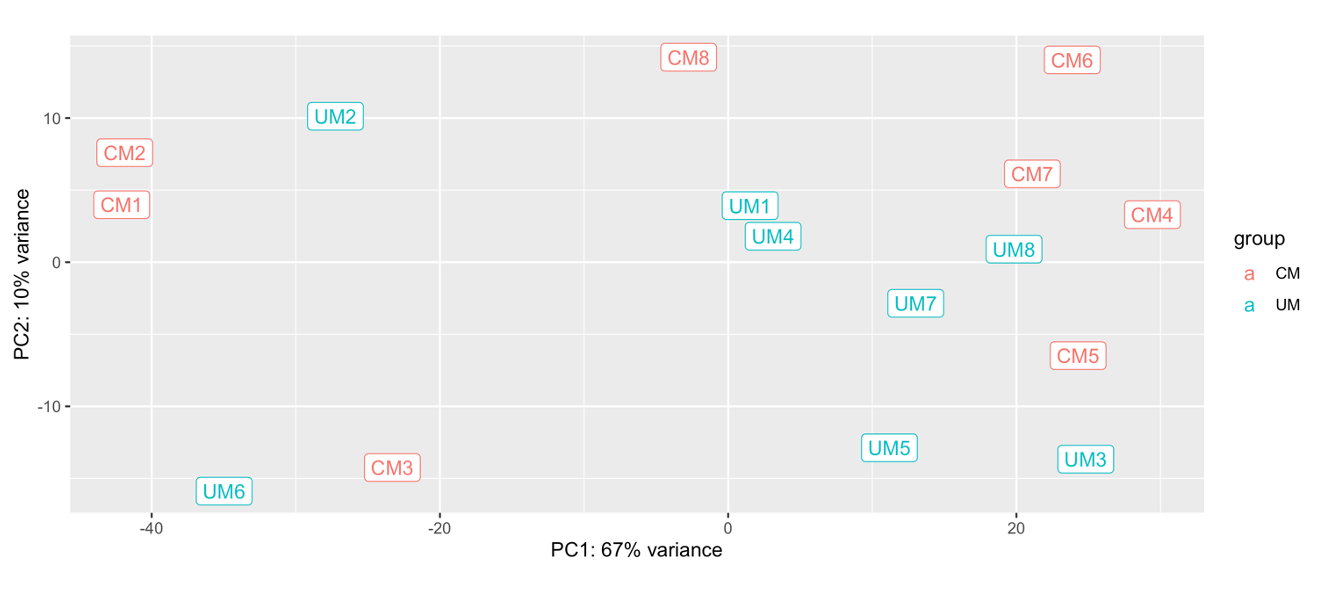
**

**Figure S4.** Principal component analysis demonstrating separation of cases of cerebral malaria and uncomplicated malaria controls with a history of cerebral malaria along the first two principal components.

**
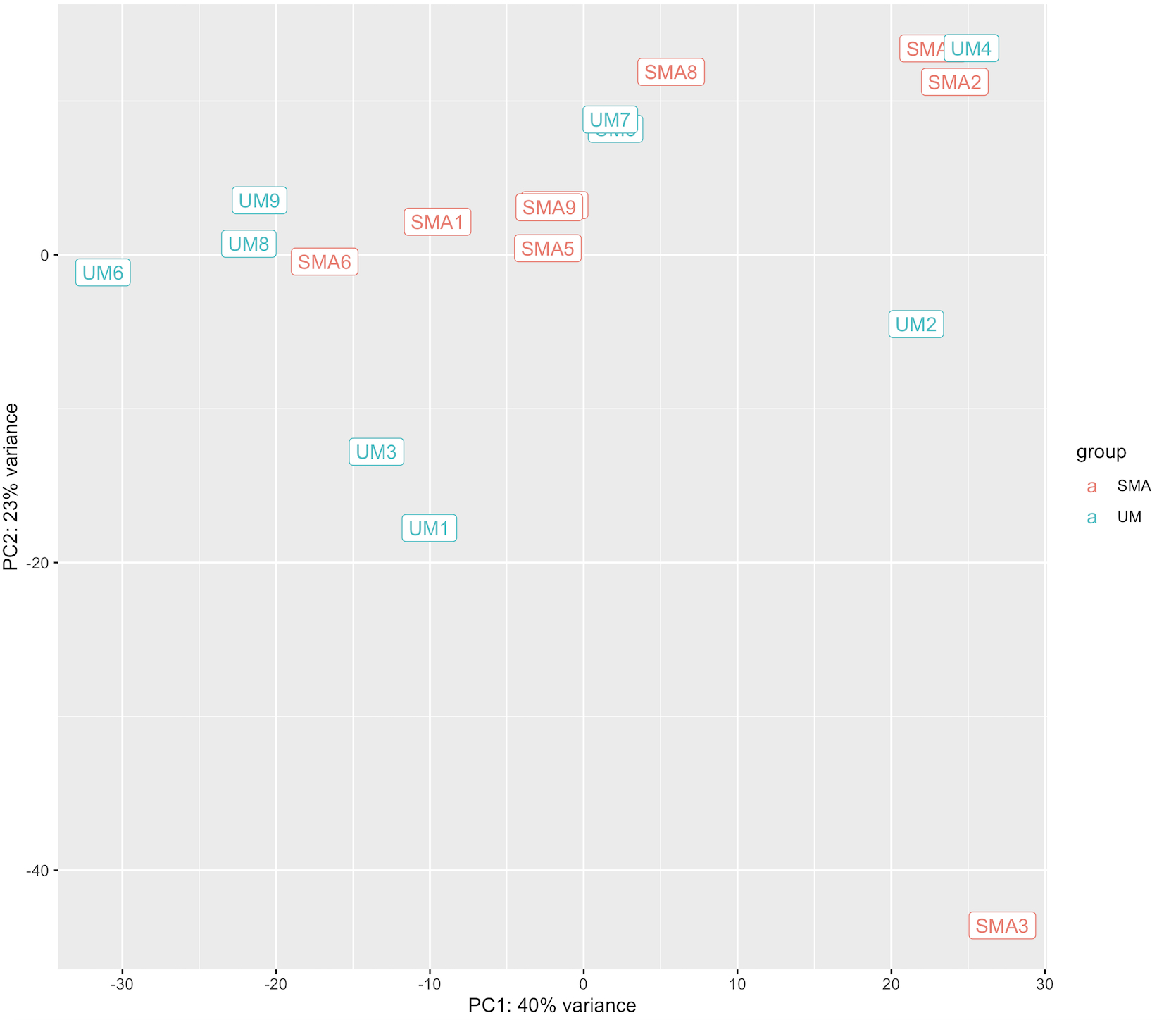
**

**Figure S5.** Principal component analysis demonstrating separation of cases of severe malarial anemia and uncomplicated malaria controls without a history of cerebral malaria along the first two principal components.


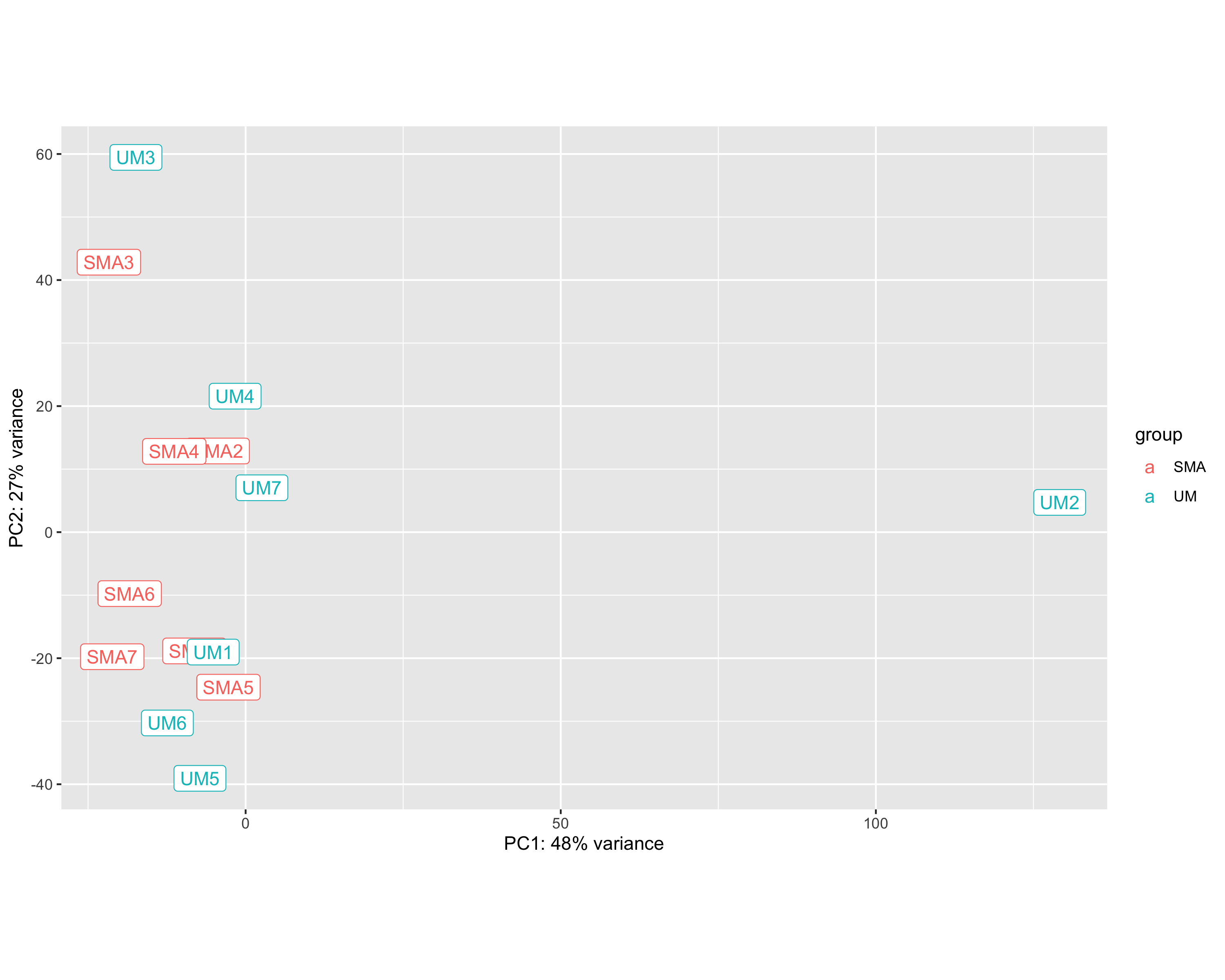

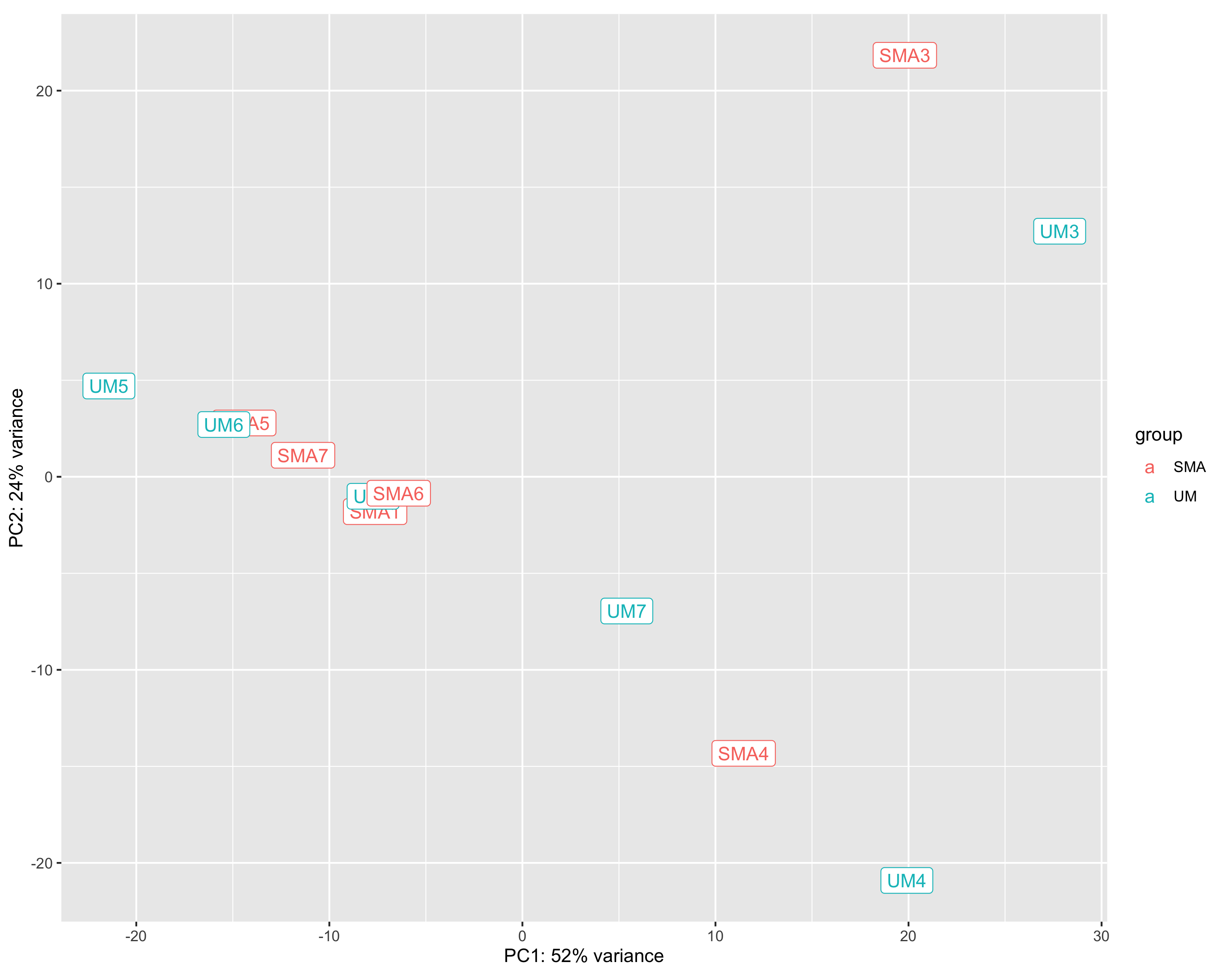


**A**

**B**

**Figure S6.** Principal component analysis demonstrating separation of cases of severe malarial anemia and uncomplicated malaria controls with a history of cerebral malaria along the first two principal components before (A) and after (B) removal of UM2.

**
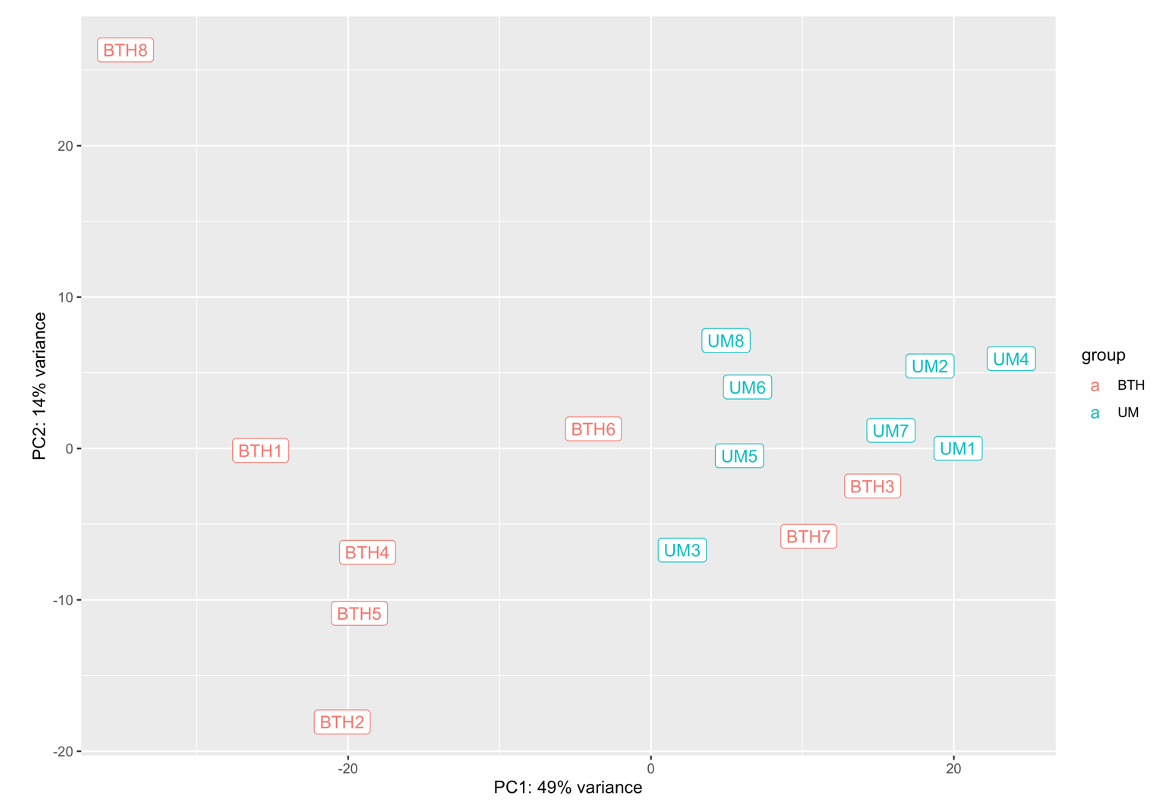
**

**Figure S7.** Principal component analysis demonstrating separation of cases of concurrent cerebral malaria and severe malarial anemia and uncomplicated malaria controls without a history of cerebral malaria.

**
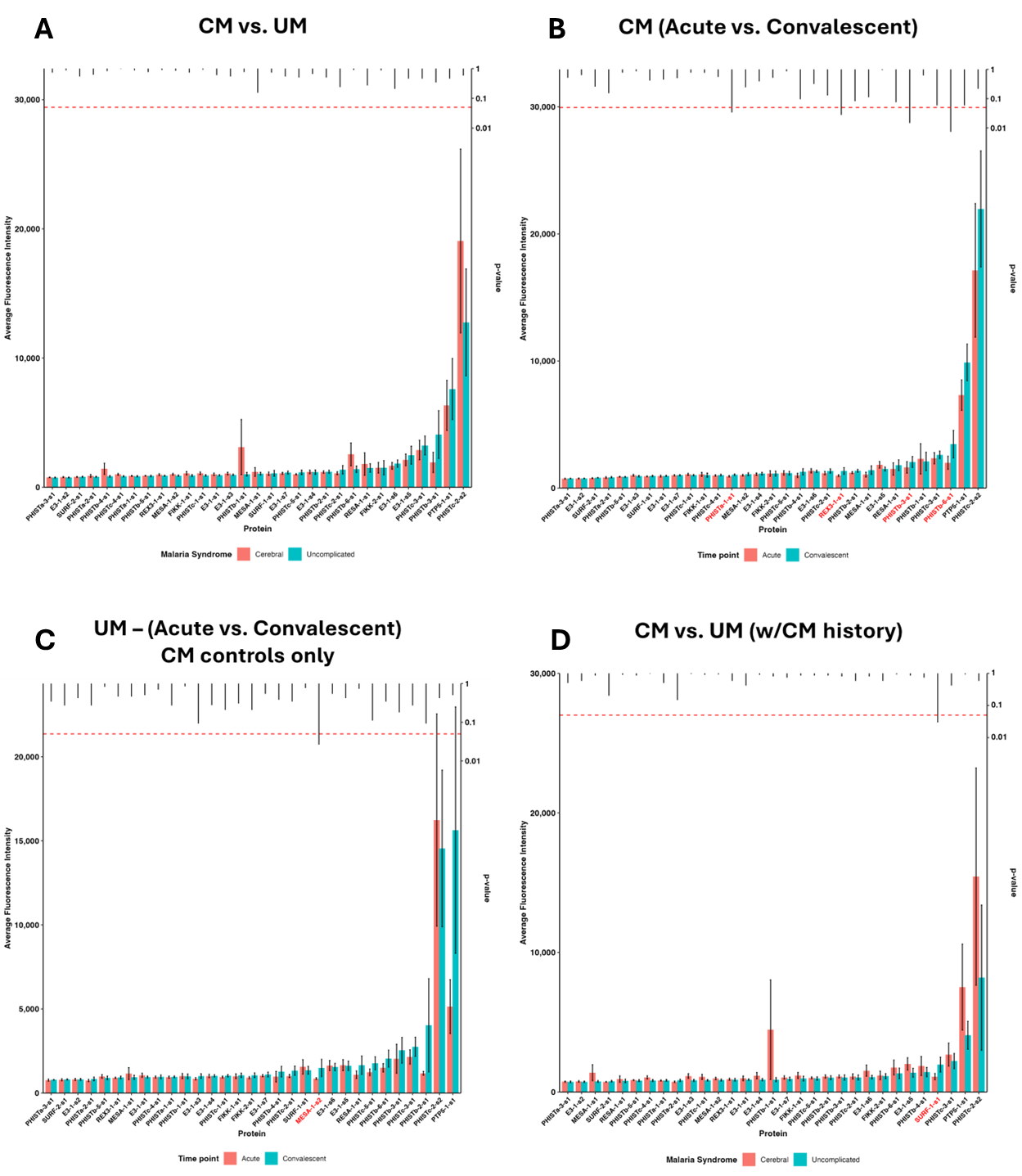
**

**Figure S8**. Serologic response comparisons on a custom protein microarray for (A) cerebral malaria subjects versus matched uncomplicated malaria controls at the time of acute illness (N=10 pairs), (B) cerebral malaria subjects at the time of acute illness versus in convalescence (N=18 pairs), (C) uncomplicated malaria controls at the time of acute illness versus in convalescence (N=10 pairs), and (D) at the time of acute illness, cerebral malaria subjects versus matched uncomplicated malaria controls who had a history of cerebral (N=6 pairs). Serologic responses are quantitated on the primary y-axis in terms of average fluorescence intensity. P-values for each comparison for each protein feature are provided on a logarithmic scale on the secondary y-axis. The dashed horizontal red line indicates a p-value of 0.05. Comparisons for which there was differential seroreactivity are indicated in red for the corresponding protein feature’s name on the x-axis.


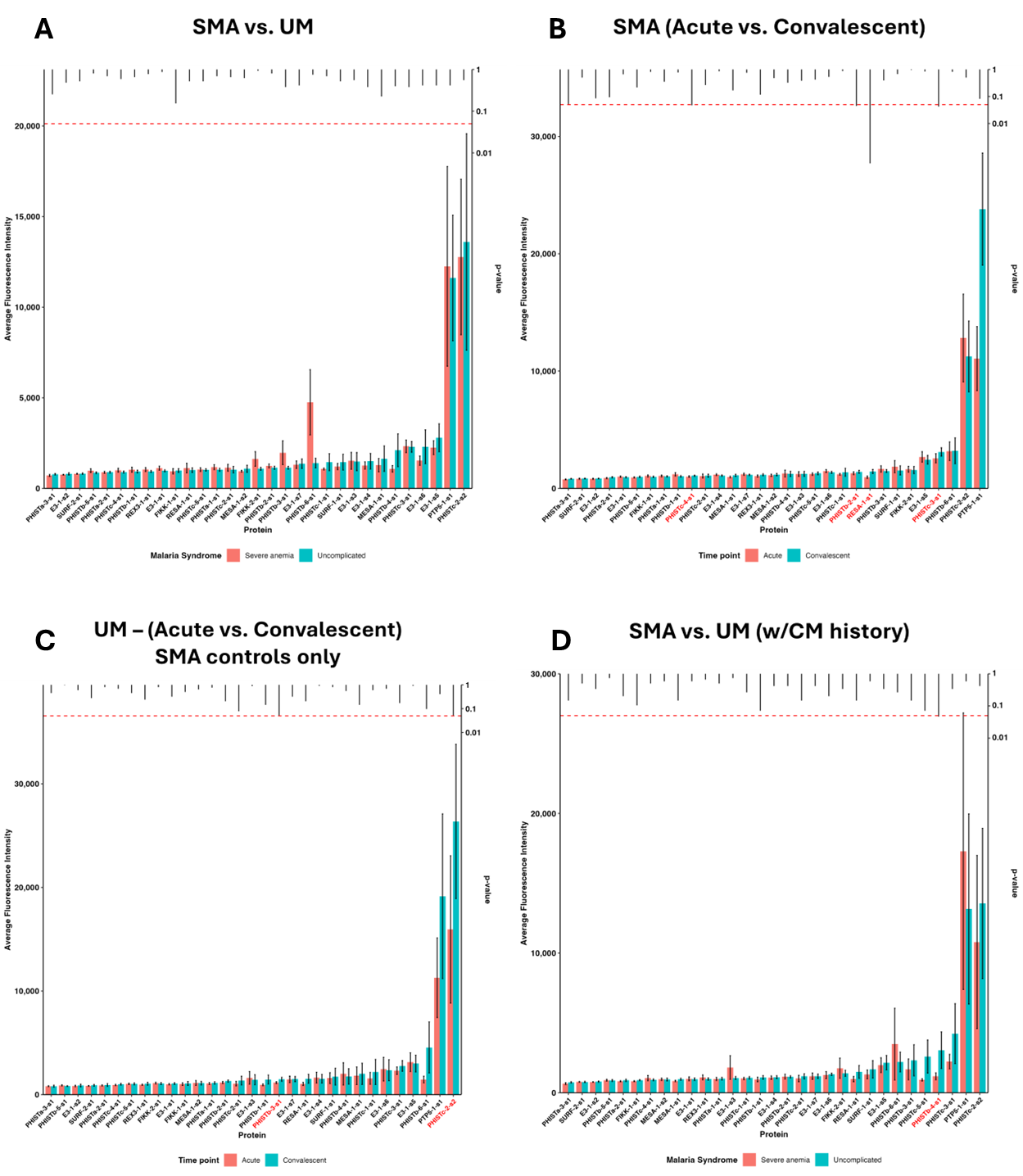


**Figure S9.** Serologic response comparisons on a custom protein microarray for (A) severe malarial anemia subjects versus matched uncomplicated malaria controls at the time of acute illness (N=11 pairs), (B) severe malarial anemia subjects at the time of acute illness versus in convalescence (N=26 pairs), (C) uncomplicated malaria controls at the time of acute illness versus in convalescence (N=9 pairs), and (D) at the time of acute illness, severe malarial anemia subjects versus matched uncomplicated malaria controls who had a history of cerebral (N=6 pairs). Serologic responses are quantitated on the primary y-axis in terms of average fluorescence intensity. P-values for each comparison for each protein feature are provided on a logarithmic scale on the secondary y-axis. The dashed horizontal red line indicates a p-value of 0.05. Comparisons for which there was differential seroreactivity are indicated in red for the corresponding protein feature’s name on the x-axis.


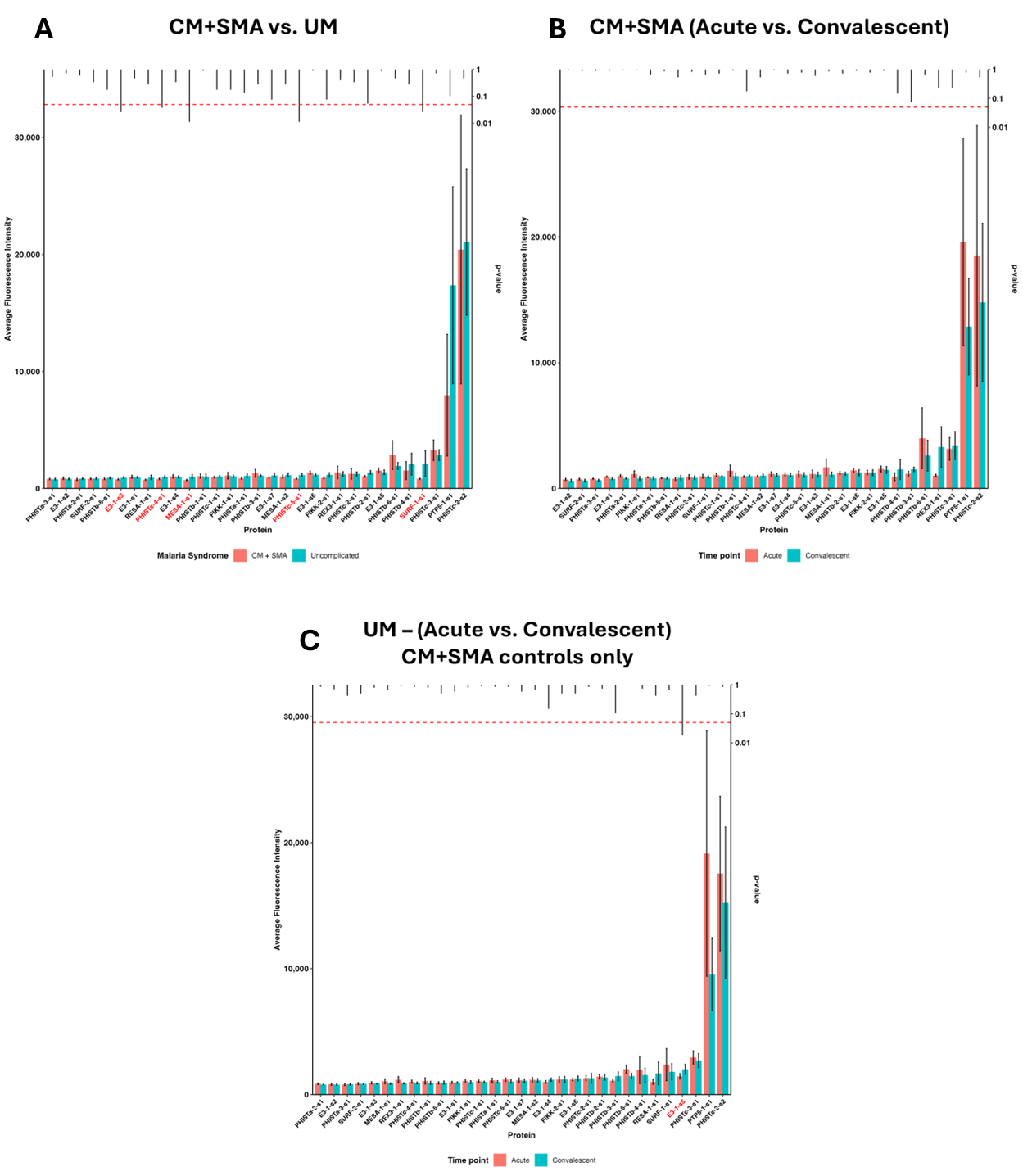


**Figure S10.** Serologic response comparisons on a custom protein microarray for (A) subjects with concurrent cerebral malaria and severe malarial anemia versus matched uncomplicated malaria controls at the time of acute illness (N=7 pairs), (B) subjects with concurrent cerebral malaria and severe malarial anemia at the time of acute illness versus in convalescence (N=7 pairs), and (C) uncomplicated malaria controls at the time of acute illness versus in convalescence (N=6 pairs). Serologic responses are quantitated on the primary y-axis in terms of average fluorescence intensity. P-values for each comparison for each protein feature are provided on a logarithmic scale on the secondary y-axis. Comparisons for which there was differential seroreactivity are indicated in red for the corresponding protein feature’s name on the x-axis.

**SUPPLEMENTARY TABLES**

| **Table S1. CIBERSORTx *P. falciparum* life-cycle stage proportions in the comparison of CM cases to uncomplicated malaria controls without a history of CM.** | | | | | | | | | | | | | |
| --- | --- | --- | --- | --- | --- | --- | --- | --- | --- | --- | --- | --- | --- |
| Sample | troph | ring | schiz | F | M |  | Mixture | troph | ring | schiz | F | M |  |
| CM1 | 0**.**133 | 0**.**867 | 0**.**000 | 0**.**000 | 0**.**000 |  | UM1 | 0**.**140 | 0**.**860 | 0**.**000 | 0**.**000 | 0**.**000 |  |
| CM2 | 0**.**240 | 0**.**760 | 0**.**000 | 0**.**000 | 0**.**000 |  | UM2 | 0**.**072 | 0**.**928 | 0**.**000 | 0**.**000 | 0**.**000 |  |
| CM3 | 0**.**004 | 0**.**996 | 0**.**000 | 0**.**000 | 0**.**000 |  | UM3 | 0**.**266 | 0**.**708 | 0**.**000 | 0**.**018 | 0**.**009 |  |
| CM4 | 0**.**000 | 0**.**913 | 0**.**087 | 0**.**000 | 0**.**001 |  | UM4 | 0**.**017 | 0**.**983 | 0**.**000 | 0**.**000 | 0**.**000 |  |
| CM5 | 0**.**116 | 0**.**884 | 0**.**000 | 0**.**000 | 0**.**000 |  | UM5 | 0**.**118 | 0**.**882 | 0**.**000 | 0**.**000 | 0**.**000 |  |
| CM6 | 0**.**130 | 0**.**870 | 0**.**000 | 0**.**000 | 0**.**000 |  | UM6 | 0**.**156 | 0**.**844 | 0**.**000 | 0**.**000 | 0**.**000 |  |
| CM7 | 0**.**078 | 0**.**916 | 0**.**006 | 0**.**000 | 0**.**000 |  | UM7 | 0**.**197 | 0**.**803 | 0**.**000 | 0**.**000 | 0**.**000 |  |
| CM8 | 0**.**014 | 0**.**983 | 0**.**004 | 0**.**000 | 0**.**000 |  | UM8 | 0**.**202 | 0**.**798 | 0**.**000 | 0**.**000 | 0**.**000 |  |
| CM9 | 0**.**067 | 0**.**933 | 0**.**000 | 0**.**000 | 0**.**000 |  | UM9 | 0**.**155 | 0**.**845 | 0**.**000 | 0**.**000 | 0**.**000 |  |
| CM10 | 0**.**047 | 0**.**953 | 0**.**000 | 0**.**000 | 0**.**000 |  | UM10 | 0**.**084 | 0**.**916 | 0**.**000 | 0**.**000 | 0**.**000 |  |
| CM11 | 0**.**010 | 0**.**965 | 0**.**025 | 0**.**000 | 0**.**000 |  | UM11 | 0**.**316 | 0**.**657 | 0**.**000 | 0**.**009 | 0**.**019 |  |
| CM12 | 0**.**001 | 0**.**999 | 0**.**000 | 0**.**000 | 0**.**000 |  | UM12 | 0**.**147 | 0**.**853 | 0**.**000 | 0**.**001 | 0**.**000 |  |
| CM13 | 0**.**150 | 0**.**850 | 0**.**000 | 0**.**000 | 0**.**000 |  | UM13 | 0**.**129 | 0**.**871 | 0**.**000 | 0**.**000 | 0**.**000 |  |
| CM14 | 0**.**181 | 0**.**819 | 0**.**000 | 0**.**000 | 0**.**000 |  | UM14 | 0**.**293 | 0**.**683 | 0**.**000 | 0**.**022 | 0**.**001 |  |

CM, cerebral malaria; troph, trophozoite; ring, ring stage; schiz, schizont; F, female gametocyte; M, male gametocyte

| **Table S2. CIBERSORTx *P. falciparum* life-cycle stage proportions in the comparison of CM cases to uncomplicated malaria controls with a history of CM.** | | | | | | | | | | | | |
| --- | --- | --- | --- | --- | --- | --- | --- | --- | --- | --- | --- | --- |
| Sample | troph | ring | schiz | F | M |  | Sample | troph | ring | schiz | F | M |
| CM1 | 0**.**134 | 0**.**866 | 0**.**000 | 0**.**000 | 0**.**000 |  | UM1 | 0**.**113 | 0**.**887 | 0**.**000 | 0**.**000 | 0**.**000 |
| CM2 | 0**.**241 | 0**.**759 | 0**.**000 | 0**.**000 | 0**.**000 |  | UM2 | 0**.**171 | 0**.**829 | 0**.**000 | 0**.**000 | 0**.**000 |
| CM3 | 0**.**135 | 0**.**865 | 0**.**000 | 0**.**000 | 0**.**000 |  | UM3 | 0**.**095 | 0**.**905 | 0**.**000 | 0**.**000 | 0**.**000 |
| CM4 | 0**.**019 | 0**.**977 | 0**.**004 | 0**.**000 | 0**.**000 |  | UM4 | 0**.**145 | 0**.**855 | 0**.**000 | 0**.**000 | 0**.**000 |
| CM5 | 0**.**044 | 0**.**956 | 0**.**000 | 0**.**000 | 0**.**000 |  | UM5 | 0**.**168 | 0**.**832 | 0**.**000 | 0**.**000 | 0**.**000 |
| CM6 | 0**.**016 | 0**.**959 | 0**.**025 | 0**.**000 | 0**.**000 |  | UM6 | 0**.**245 | 0**.**755 | 0**.**000 | 0**.**000 | 0**.**000 |
| CM7 | 0**.**006 | 0**.**994 | 0**.**000 | 0**.**000 | 0**.**000 |  | UM7 | 0**.**189 | 0**.**810 | 0**.**000 | 0**.**001 | 0**.**000 |
| CM8 | 0**.**174 | 0**.**826 | 0**.**000 | 0**.**000 | 0**.**000 |  | UM8 | 0**.**103 | 0**.**896 | 0**.**000 | 0**.**001 | 0**.**000 |

CM, cerebral malaria; troph, trophozoite; ring, ring stage; schiz, schizont; F, female gametocyte; M, male gametocyte

| **Table S3. CIBERSORTx *P. falciparum* life-cycle stage proportions in the comparison of SMA cases to uncomplicated malaria controls without a history of CM.** | | | | | | | | | | | | |
| --- | --- | --- | --- | --- | --- | --- | --- | --- | --- | --- | --- | --- |
| Sample | troph | ring | schiz | F | M |  | Sample | troph | ring | schiz | F | M |
| SMA1 | 0**.**078 | 0**.**922 | 0**.**000 | 0**.**000 | 0**.**000 |  | UM1 | 0**.**158 | 0**.**827 | 0**.**000 | 0**.**016 | 0**.**000 |
| SMA2 | 0**.**108 | 0**.**892 | 0**.**000 | 0**.**000 | 0**.**000 |  | UM2 | 0**.**147 | 0**.**844 | 0**.**000 | 0**.**009 | 0**.**000 |
| SMA3 | 0**.**342 | 0**.**539 | 0**.**000 | 0**.**102 | 0**.**016 |  | UM3 | 0**.**140 | 0**.**854 | 0**.**000 | 0**.**005 | 0**.**000 |
| SMA4 | 0**.**108 | 0**.**892 | 0**.**000 | 0**.**000 | 0**.**000 |  | UM4 | 0**.**142 | 0**.**858 | 0**.**000 | 0**.**000 | 0**.**000 |
| SMA5 | 0**.**148 | 0**.**852 | 0**.**000 | 0**.**000 | 0**.**000 |  | UM5 | 0**.**142 | 0**.**858 | 0**.**000 | 0**.**000 | 0**.**000 |
| SMA6 | 0**.**033 | 0**.**965 | 0**.**002 | 0**.**000 | 0**.**000 |  | UM6 | 0**.**013 | 0**.**987 | 0**.**000 | 0**.**000 | 0**.**000 |
| SMA7 | 0**.**066 | 0**.**934 | 0**.**000 | 0**.**000 | 0**.**000 |  | UM7 | 0**.**121 | 0**.**879 | 0**.**000 | 0**.**000 | 0**.**000 |
| SMA8 | 0**.**203 | 0**.**797 | 0**.**000 | 0**.**000 | 0**.**000 |  | UM8 | 0**.**065 | 0**.**933 | 0**.**002 | 0**.**000 | 0**.**000 |
| SMA9 | 0**.**092 | 0**.**908 | 0**.**000 | 0**.**000 | 0**.**000 |  | UM9 | 0**.**000 | 0**.**992 | 0**.**008 | 0**.**000 | 0**.**000 |

CM, cerebral malaria; SMA; severe malarial anemia; Troph, trophozoite; ring, ring stage; schiz, schizont; F, female gametocyte; M, male gametocyte

| **Table S4. CIBERSORTx *P. falciparum* life-cycle stage proportions in the comparison of SMA cases to uncomplicated malaria controls with a history of CM.** | | | | | | | | | | | | |
| --- | --- | --- | --- | --- | --- | --- | --- | --- | --- | --- | --- | --- |
| Sample | troph | ring | schiz | F | M |  | Sample | troph | ring | schiz | F | M |
| SMA1 | 0**.**093 | 0**.**907 | 0**.**000 | 0**.**000 | 0**.**000 |  | UM1 | 0**.**189 | 0**.**811 | 0**.**000 | 0**.**000 | 0**.**000 |
| SMA3 | 0**.**330 | 0**.**550 | 0**.**001 | 0**.**101 | 0**.**017 |  | UM3 | 0**.**226 | 0**.**649 | 0**.**001 | 0**.**099 | 0**.**025 |
| SMA4 | 0**.**106 | 0**.**894 | 0**.**000 | 0**.**000 | 0**.**000 |  | UM4 | 0**.**085 | 0**.**915 | 0**.**000 | 0**.**000 | 0**.**000 |
| SMA5 | 0**.**035 | 0**.**963 | 0**.**002 | 0**.**000 | 0**.**000 |  | UM5 | 0**.**000 | 1**.**000 | 0**.**000 | 0**.**000 | 0**.**000 |
| SMA6 | 0**.**071 | 0**.**929 | 0**.**000 | 0**.**000 | 0**.**000 |  | UM6 | 0**.**070 | 0**.**930 | 0**.**000 | 0**.**000 | 0**.**000 |
| SMA7 | 0**.**065 | 0**.**930 | 0**.**005 | 0**.**000 | 0**.**000 |  | UM7 | 0**.**134 | 0**.**864 | 0**.**000 | 0**.**002 | 0**.**000 |

CM, cerebral malaria; SMA; severe malarial anemia; troph, trophozoite; ring, ring stage; schiz, schizont; F, female gametocyte; M, male gametocyte

| **Table S5. CIBERSORTx *P. falciparum* life-cycle stage proportions in the comparison of concurrent CM and SMA cases to uncomplicated malaria controls without a history of CM.** | | | | | | | | | | | | |
| --- | --- | --- | --- | --- | --- | --- | --- | --- | --- | --- | --- | --- |
| Sample | troph | ring | schiz | F | M |  | Sample | troph | ring | schiz | F | M |
| BTH1 | 0**.**318 | 0**.**639 | 0**.**027 | 0**.**007 | 0**.**010 |  | UM1 | 0**.**003 | 0**.**997 | 0**.**000 | 0**.**000 | 0**.**000 |
| BTH2 | 0**.**215 | 0**.**771 | 0**.**000 | 0**.**013 | 0**.**001 |  | UM2 | 0**.**120 | 0**.**880 | 0**.**000 | 0**.**000 | 0**.**000 |
| BTH3 | 0**.**028 | 0**.**972 | 0**.**000 | 0**.**000 | 0**.**000 |  | UM3 | 0**.**160 | 0**.**840 | 0**.**000 | 0**.**000 | 0**.**000 |
| BTH4 | 0**.**144 | 0**.**855 | 0**.**000 | 0**.**000 | 0**.**001 |  | UM4 | 0**.**118 | 0**.**881 | 0**.**000 | 0**.**001 | 0**.**000 |
| BTH5 | 0**.**234 | 0**.**766 | 0**.**000 | 0**.**000 | 0**.**000 |  | UM5 | 0**.**229 | 0**.**771 | 0**.**000 | 0**.**000 | 0**.**000 |
| BTH6 | 0**.**159 | 0**.**803 | 0**.**037 | 0**.**000 | 0**.**001 |  | UM6 | 0**.**330 | 0**.**644 | 0**.**026 | 0**.**000 | 0**.**000 |
| BTH7 | 0**.**065 | 0**.**935 | 0**.**000 | 0**.**000 | 0**.**000 |  | UM7 | 0**.**101 | 0**.**899 | 0**.**000 | 0**.**000 | 0**.**000 |
| BTH8 | 0**.**331 | 0**.**667 | 0**.**002 | 0**.**000 | 0**.**000 |  | UM8 | 0**.**263 | 0**.**675 | 0**.**000 | 0**.**054 | 0**.**008 |

| **Table S6. Comprehensive sample characteristics for the comparison of CM cases to uncomplicated malaria controls without a history of CM.** | | | | | | | | | | | | | | | | | | |
| --- | --- | --- | --- | --- | --- | --- | --- | --- | --- | --- | --- | --- | --- | --- | --- | --- | --- | --- |
| Name | Type | Match_ID | Match_group | Enrol__date | annee | Sex | Age | Ethnicity | Residency | BCS | Hb__g_dl_ | GE | Total Reads | Site | Blood_type | Rh_factor | Type_cat | De-identified_ID |
| CM1 | 1 | 2 | CM | 29-Oct-14 | 2014 | Male | 4 years | Dogon | B3 | 2 | 6.6 | 14040 | 155779332 | Bandiagara | A | + | case | CM11 |
| UM1 | 2 | 2 | CM | 30-Nov-14 | 2014 | Male | 4 years | Dogon | B2 | 5 | 10.7 | 3900 | 14864116 | Bandiagara | A | + | control | UMC51 |
| CM2 | 1 | 3 | CM | 7-Oct-15 | 2015 | Male | 4 years | Dogon | Tegourou | 1 | 6.9 | 76400 | 155208138 | Bandiagara | O | + | case | CM12 |
| UM2 | 2 | 3 | CM | 5-Nov-15 | 2015 | Male | 4 years | Dogon | Tegourou | 5 | 8.6 | 9000 | 50760778 | Bandiagara | A | + | control | UMC1 |
| CM3 | 1 | 4 | CM | 7-Oct-15 | 2015 | Female | 18 months | Dogon | Dobolo | 2 | 7.4 | 73800 | 75412614 | Bandiagara | O | + | case | CM13 |
| UM3 | 2 | 4 | CM | 11-Nov-15 | 2015 | Female | 21 Months | Dogon | Dobolo | 5 | 5.5 | 3300 | 26846288 | Bandiagara | A | + | control | UMC2 |
| CM4 | 1 | 5 | CM | 9-Oct-15 | 2015 | Male | 16 months | Dogon | Andjoumbolo | 2 | 5.4 | 24000 | 66902626 | Bandiagara | O | + | case | CM14 |
| UM4 | 2 | 5 | CM | 6-Nov-15 | 2015 | Male | 16 Months | Dogon | Andjoumbolo | 5 | 7.5 | 1425 | 44453676 | Bandiagara | B | + | control | UMC3 |
| CM5 | 1 | 13 | CM | 1-Feb-16 | 2016 | Female | 1 year | Dogon | Ningari Sanabougou | 2 | 7.9 | 291000 | 196238208 | Bandiagara | A | + | case | CM1 |
| UM5 | 2 | 13 | CM | 13-Feb-16 | 2016 | Female | 1 years | Dogon | Ningari Sanabougou | 5 | 7.9 | 82500 | 71765800 | Bandiagara | A | + | control | UMC10 |
| CM6 | 1 | 22 | CM | 12-Aug-16 | 2016 | Female | 3 years | Dogon | Eguela | 2 | 5.3 | 18150 | 90079940 | Bandiagara | A | + | case | CM2 |
| UM6 | 2 | 22 | CM | 20-Sep-16 | 2016 | Female | 3 years | Dogon | Eguela | 5 | 11.6 | 45850 | 156187024 | Bandiagara | O | + | control | UMC13 |
| CM7 | 1 | 27 | CM | 25-Aug-16 | 2016 | Female | 3years | Dogon | Kolontagna | 2 | 9 | 25350 | 45669936 | Bandiagara | O | + | case | CM3 |
| UM7 | 2 | 27 | CM | 13-Oct-16 | 2016 | Female | 3 years | Dogon | Kolontagna | 5 | 8.8 | 54000 | 56097596 | Bandiagara | O | + | control | UMC16 |
| CM8 | 1 | 28 | CM | 27-Aug-16 | 2016 | Male | 2 years | Dogon | Ebèguèrè | 2 | 9.6 | 265800 | 132024738 | Bandiagara | O | + | case | CM4 |
| UM8 | 2 | 28 | CM | 4-Oct-16 | 2016 | Male | 2 years | Dogon | Ebèguèrè | 5 | 10.6 | 5100 | 25328982 | Bandiagara | O | + | control | UMC17 |
| CM9 | 1 | 29 | CM | 29-Aug-16 | 2016 | Female | 5 years | Bambara | Yirimadio | 2 | 11.6 | 11680 | 75852434 | HDM-Bamako | B | + | case | CM9 |
| UM9 | 2 | 29 | CM | 7-Oct-16 | 2016 | Female | 4 years | Bambara | Yirimadio | 5 | 5.8 | 2400 | 23754548 | HDM-Bamako | O | + | control | UMC18 |
| CM10 | 1 | 33 | CM | 5-Sep-16 | 2016 | Male | 3 years | Dogon | Kolontagna | 2 | 8.9 | 442800 | 240676256 | Bandiagara | B | + | case | CM5 |
| UM10 | 2 | 33 | CM | 25-Sep-16 | 2016 | Male | 3 years | Dogon | Kolontagna | 5 | 10.6 | 165000 | 246860744 | Bandiagara | O | + | control | UMC22 |
| CM11 | 1 | 35 | CM | 17-Sep-16 | 2016 | Male | 3 years | Dogon | Golo | 2 | 7 | 450600 | 123149726 | Bandiagara | AB | + | case | CM6 |
| UM11 | 2 | 35 | CM | 21-Jan-17 | 2017 | Male | 3 years | Dogon | Golo | 5 | 6.6 | 14700 | 82120128 | Bandiagara | B | + | control | UMC23 |
| CM12 | 1 | 40 | CM | 24-Oct-16 | 2016 | Male | 5 years | Dogon | Soroli | 1 | 8.1 | 244700 | 214685758 | Bandiagara | AB | + | case | CM7 |
| UM12 | 2 | 40 | CM | 18-Jan-17 | 2017 | Male | 4 years | Dogon | Soroli | 5 | 8 | 2850 | 45681256 | Bandiagara | A | + | control | UMC26 |
| CM13 | 1 | 41 | CM | 26-Sep-16 | 2016 | Female | 9 months | Bambara | Yirimadio-Zerni | 2 | 6.3 | 42000 | 154627892 | HDM-Bamako | O | + | case | CM10 |
| UM13 | 2 | 41 | CM | 9-Nov-16 | 2016 | Female | 15 months | Bambara | Yirimadio-Zerni | 5 | 8.4 | 22500 | 41053522 | HDM-Bamako | O | - | control | UMC45 |
| CM14 | 1 | 42 | CM | 8-Nov-16 | 2016 | Male | 3 years | Dogon | Dibo | 1 | 10.8 | 1200 | 17766120 | Bandiagara | O | - | case | CM8 |
| UM14 | 2 | 42 | CM | 14-Dec-16 | 2016 | Male | 4 years | Dogon | Dibo | 5 | 8.2 | 5475 | 26843032 | Bandiagara | O | + | control | UMC27 |

CM, cerebral malaria; type; type of participant 1=case; 2=control without history of cerebral malaria; 3=control with history of cerebral malaria; annee, year of enrollment; BCS, Blantyre coma score; GE, parasitemia in parasites per microliter.

| **Table S7. Comprehensive sample characteristics for the comparison of CM cases to uncomplicated malaria controls with a history of CM.** | | | | | | | | | | | | | | | | | | |
| --- | --- | --- | --- | --- | --- | --- | --- | --- | --- | --- | --- | --- | --- | --- | --- | --- | --- | --- |
| Name | Type | Match_ID | Match_group | Enrol__date | annee | Sex | Age | Ethnicity | Residency | BCS | Hb__g_dl_ | GE | Total Reads | Site | Blood_type | Rh_factor | Type_cat | De-identified_ID |
| CM1 | 1 | 2 | CM | 29-Oct-14 | 2014 | Male | 4 years | Dogon | B3 | 2 | 6.6 | 14040 | 155779332 | Bandiagara | A | + | case | CM11 |
| UM1 | 3 | 2 | CM | 18-Dec-14 | 2014 | Male | 4 years | Dogon | B5 | 5 | 9.7 | 158400 | 107419230 | Bandiagara | A | + | control | UMC49 |
| CM2 | 1 | 3 | CM | 7-Oct-15 | 2015 | Male | 4 years | Dogon | Tegourou | 1 | 6.9 | 76400 | 155208138 | Bandiagara | O | + | case | CM12 |
| UM2 | 3 | 3 | CM | 5-Nov-15 | 2015 | Male | 4 years | Dogon | Tegourou | 5 | 7.8 | 10800 | 61447592 | Bandiagara | A | + | control | UMC29 |
| CM3 | 1 | 22 | CM | 12-Aug-16 | 2016 | Female | 3 years | Dogon | Eguela | 2 | 5.3 | 18150 | 90079940 | Bandiagara | A | + | case | CM2 |
| UM3 | 3 | 22 | CM | 20-Sep-16 | 2016 | Female | 3 years | Dogon | Eguela | 5 | 11.4 | 22500 | 63698020 | Bandiagara | O | + | control | UMC35 |
| CM4 | 1 | 28 | CM | 27-Aug-16 | 2016 | Male | 2 years | Dogon | Ebèguèrè | 2 | 9.6 | 265800 | 132024738 | Bandiagara | O | + | case | CM4 |
| UM4 | 3 | 28 | CM | 4-Oct-16 | 2016 | Male | 2 years | Dogon | Ebèguèrè | 5 | 8.1 | 1500 | 33710456 | Bandiagara | B | + | control | UMC38 |
| CM5 | 1 | 33 | CM | 5-Sep-16 | 2016 | Male | 3 years | Dogon | Kolontagna | 2 | 8.9 | 442800 | 240676256 | Bandiagara | B | + | case | CM5 |
| UM5 | 3 | 33 | CM | 25-Sep-16 | 2016 | Male | 3 years | Dogon | Kolontagna | 5 | 11.1 | 7350 | 20209838 | Bandiagara | O | + | control | UMC41 |
| CM6 | 1 | 35 | CM | 17-Sep-16 | 2016 | Male | 3 years | Dogon | Golo | 2 | 7 | 450600 | 123149726 | Bandiagara | AB | + | case | CM6 |
| UM6 | 3 | 35 | CM | 11-Nov-16 | 2016 | Male | 3 years | Dogon | Golo | 5 | 9.9 | 1050 | 9021440 | Bandiagara | AB | + | control | UMC42 |
| CM7 | 1 | 40 | CM | 24-Oct-16 | 2016 | Male | 5 years | Dogon | Soroli | 1 | 8.1 | 244700 | 214685758 | Bandiagara | AB | + | case | CM7 |
| UM7 | 3 | 40 | CM | 18-Jan-17 | 2017 | Male | 4 years | Dogon | Soroli | 5 | 8 | 12300 | 27647304 | Bandiagara | O | + | control | UMC43 |
| CM8 | 1 | 42 | CM | 8-Nov-16 | 2016 | Male | 3 years | Dogon | Dibo | 1 | 10.8 | 1200 | 17766120 | Bandiagara | O | - | case | CM8 |
| UM8 | 3 | 42 | CM | 14-Dec-16 | 2016 | Male | 5 years | Dogon | Dibo | 5 | 10.4 | 88800 | 177331360 | Bandiagara | O | + | control | UMC44 |

CM, cerebral malaria; type; type of participant 1=case; 2=control without history of cerebral malaria; 3=control with history of cerebral malaria; annee, year of enrollment; BCS, Blantyre coma score; GE, parasitemia in parasites per microliter.

| **Table S8. Comprehensive sample characteristics for the comparison of SMA cases to uncomplicated malaria controls without a history of CM.** | | | | | | | | | | | | | | | | | | |
| --- | --- | --- | --- | --- | --- | --- | --- | --- | --- | --- | --- | --- | --- | --- | --- | --- | --- | --- |
| Sample Name | Type | Match_ID | Match_group | Enrol__date | annee | Sex | Age | Ethnicity | Residency | BCS | Hb__g_dl_ | GE | Total Reads | Site | Blood_type | Rh_factor | Type_cat | De-identified_ID |
| SMA1 | 1 | 9 | SMA | 31-Oct-15 | 2015 | Female | 3 years | Dogon | Siby-Siby | 3 | 3.8 | 68400 | 157684308 | Bandiagara | B | + | case | SMA11 |
| UM1 | 2 | 9 | SMA | 4-Nov-15 | 2015 | Female | 3 years | Dogon | Siby-Siby | 5 | 7 | 55500 | 18747278 | Bandiagara | B | + | control | UMC6 |
| SMA2 | 1 | 10 | SMA | 10-Nov-15 | 2015 | Female | 3 years | Dogon | Pouroly | 5 | 3.6 | 43800 | 192586382 | Bandiagara | O | + | case | SMA1 |
| UM2 | 2 | 10 | SMA | 16-Nov-15 | 2015 | Female | 3 years | Dogon | Pouroly | 5 | 8.6 | 9300 | 67090758 | Bandiagara | AB | + | control | UMC7 |
| SMA3 | 1 | 11 | SMA | 23-Nov-15 | 2015 | Female | 3 years | Dogon | Siby-Siby | 5 | 2.3 | 8550 | 39389948 | Bandiagara | B | + | case | SMA2 |
| UM3 | 2 | 11 | SMA | 11-Dec-15 | 2015 | Female | 3 years | Dogon | Siby-Siby | 5 | 8.9 | 55800 | 71559418 | Bandiagara | O | + | control | UMC8 |
| SMA4 | 1 | 12 | SMA | 27-Nov-15 | 2015 | Male | 3 years | Dogon | Doucoumbo | 5 | 2.8 | 181500 | 185503632 | Bandiagara | B | + | case | SMA3 |
| UM4 | 2 | 12 | SMA | 12-Feb-16 | 2016 | Male | 3 years | Dogon | Doucoumbo | 5 | 11.5 | 9975 | 21765062 | Bandiagara | A | + | control | UMC9 |
| SMA5 | 1 | 17 | SMA | 5-Aug-16 | 2016 | Female | 2 years | Dogon | Djombo Peulh | 3 | 2.9 | 55500 | 209353056 | Bandiagara | B | + | case | SMA4 |
| UM5 | 2 | 17 | SMA | 18-Oct-16 | 2016 | Female | 3 years | Dogon | Djombo Peulh | 5 | 7.2 | 119400 | 142523088 | Bandiagara | A | + | control | UMC12 |
| SMA6 | 1 | 25 | SMA | 22-Aug-16 | 2016 | Female | 5 years | Dogon | Anakanda | 4 | 5 | 95100 | 82748558 | Bandiagara | B | + | case | SMA5 |
| UM6 | 2 | 25 | SMA | 10-Nov-16 | 2016 | Female | 5 years | Dogon | Anakanda | 5 | 11.2 | 1050 | 3148120 | Bandiagara | O | + | control | UMC15 |
| SMA7 | 1 | 30 | SMA | 31-Aug-16 | 2016 | Female | 1 year | Dogon | Sasadi | 5 | 2.6 | 3450 | 19376896 | Bandiagara | O | + | case | SMA7 |
| UM7 | 2 | 30 | SMA | 5-Oct-16 | 2016 | Female | 1 year | Dogon | Sasadi | 5 | 6.2 | 1350 | 107311308 | Bandiagara | B | - | control | UMC19 |
| SMA8 | 1 | 44 | SMA | 23-Dec-16 | 2016 | Female | 1 year | Dogon | Tognon | 4 | 4.2 | 143750 | 66815734 | Bandiagara | B | - | case | SMA6 |
| UM8 | 2 | 44 | SMA | 17-Jan-17 | 2017 | Female | 1 year | Dogon | Tognon | 5 | 8.5 | 5850 | 135240596 | Bandiagara | O | + | control | UMC28 |
| SMA9 | 1 | 57 | SMA | 27-Nov-16 | 2016 | Female | 3 years | Peulh | Yirimadio | 3 | 2.6 | 17500 | 68466686 | HDM-Bamako | B | + | case | SMA10 |
| UM9 | 2 | 57 | SMA | 22-Dec-16 | 2016 | Female | 3 years | Peulh | Yirimadio | 5 | 10.5 | 14475 | 80885222 | HDM-Bamako | O | + | control | UMC47 |

SMA, severe malarial anemia; CM, cerebral malaria; type; type of participant 1=case; 2=control without history of cerebral malaria; 3=control with history of cerebral malaria; annee, year of enrollment; BCS, Blantyre coma score; GE, parasitemia in parasites per microliter.

| **Table S9. Comprehensive sample characteristics for the comparison of SMA cases to uncomplicated malaria controls with a history of CM.** | | | | | | | | | | | | | | | | | | |
| --- | --- | --- | --- | --- | --- | --- | --- | --- | --- | --- | --- | --- | --- | --- | --- | --- | --- | --- |
| Name | Type | Match_ID | Match_group | Enrol__date | annee | Sex | Age | Ethnicity | Residency | BCS | Hb__g_dl_ | GE | Total Reads | Site | Blood_type | Rh_factor | Type_cat | De-identified_ID |
| SMA1 | 1 | 9 | SMA | 31-Oct-15 | 2015 | Female | 3 years | Dogon | Siby-Siby | 3 | 3.8 | 68400 | 157684308 | Bandiagara | B | + | case | SMA11 |
| UM1 | 3 | 9 | SMA | 4-Nov-15 | 2015 | Female | 3 years | Dogon | Siby-Siby | 5 | 7.7 | 14700 | 221935588 | Bandiagara | O | + | control | UMC50 |
| SMA3 | 1 | 11 | SMA | 23-Nov-15 | 2015 | Female | 3 years | Dogon | Siby-Siby | 5 | 2.3 | 8550 | 39389948 | Bandiagara | B | + | case | SMA2 |
| UM3 | 3 | 11 | SMA | 14-Mar-16 | 2016 | Female | 3 years | Dogon | Siby-Siby | 5 | 9.3 | 900 | 8235308 | Bandiagara | B | + | control | UMC33 |
| SMA4 | 1 | 12 | SMA | 27-Nov-15 | 2015 | Male | 3 years | Dogon | Doucoumbo | 5 | 2.8 | 181500 | 185503632 | Bandiagara | B | + | case | SMA3 |
| UM4 | 3 | 12 | SMA | 15-Mar-16 | 2016 | Male | 3 years | Dogon | Doucoumbo | 5 | 9.3 | 149400 | 199533686 | Bandiagara | B | + | control | UMC34 |
| SMA5 | 1 | 25 | SMA | 22-Aug-16 | 2016 | Female | 5 years | Dogon | Anakanda | 4 | 5 | 95100 | 82748558 | Bandiagara | B | + | case | SMA5 |
| UM5 | 3 | 25 | SMA | 24-Sep-16 | 2016 | Female | 5 years | Dogon | Anakanda | 5 | 11.6 | 192000 | 232623818 | Bandiagara | B | + | control | UMC37 |
| SMA6 | 1 | 30 | SMA | 31-Aug-16 | 2016 | Female | 1 year | Dogon | Sasadi | 5 | 2.6 | 3450 | 19376896 | Bandiagara | O | + | case | SMA7 |
| UM6 | 3 | 30 | SMA | 13-Nov-16 | 2016 | Female | 1 year | Dogon | Sasadi | 5 | 8.3 | 4050 | 12996460 | Bandiagara | A | + | control | UMC39 |
| SMA7 | 1 | 43 | SMA | 26-Sep-16 | 2016 | Male | 3 years | Bambara | Yirimadio | 5 | 2.8 | 28350 | 29800346 | HDM-Bamako | AB | + | case | SMA8 |
| UM7 | 3 | 43 | SMA | 15-Nov-16 | 2016 | Male | 3 years | Bambara | Yirimadio | 5 | 5.9 | 525 | 4475634 | HDM-Bamako | O | + | control | UMC48 |

SMA, severe malarial anemia; CM, cerebral malaria; type; type of participant 1=case; 2=control without history of cerebral malaria; 3=control with history of cerebral malaria; annee, year of enrollment; BCS, Blantyre coma score; GE, parasitemia in parasites per microliter

| **Table S10. Comprehensive sample characteristics for the comparison of concurrent CM and SMA cases to uncomplicated malaria controls without a history of CM.** | | | | | | | | | | | | | | | | | | |
| --- | --- | --- | --- | --- | --- | --- | --- | --- | --- | --- | --- | --- | --- | --- | --- | --- | --- | --- |
| Name | Type | Match_ID | Match_group | Enrol__date | annee | Sex | Age | Ethnicity | Residency | BCS | Hb__g_dl_ | GE | Total Reads | Site | Blood_type | Rh_factor | Type_cat | De-identified_ID |
| BTH1 | 1 | 6 | CM + SMA | 14-Oct-15 | 2015 | Male | 3 years | Dogon | Guine-wolo | 1 | 2.5 | 525 | 33038818 | Bandiagara | B | + | case | CM+SMA7 |
| UM1 | 2 | 6 | CM + SMA | 12-Nov-15 | 2015 | Male | 3 years | Dogon | Guine-wolo | 5 | 11.2 | 1575 | 20042888 | Bandiagara | B | + | control | UMC4 |
| BTH2 | 1 | 8 | CM + SMA | 23-Oct-15 | 2015 | Male | 4years | Dogon | Kori-Kori | 1 | 2.6 | 950 | 21922384 | Bandiagara | AB | + | case | CM+SMA8 |
| UM2 | 2 | 8 | CM + SMA | 9-Nov-15 | 2015 | Male | 4 years | Dogon | Kori-Kori | 5 | 7.8 | 375 | 69962660 | Bandiagara | O | + | control | UMC5 |
| BTH3 | 1 | 14 | CM + SMA | 10-Feb-16 | 2016 | Male | 1 year | Dogon | Tegourou | 2 | 3.5 | 242400 | 175540710 | Bandiagara | B | + | case | CM+SMA1 |
| UM3 | 2 | 14 | CM + SMA | 12-Mar-16 | 2016 | Male | 1 years | Dogon | Tegourou | 5 | 7 | 12600 | 19504116 | Bandiagara | B | + | control | UMC11 |
| BTH4 | 1 | 23 | CM + SMA | 19-Aug-16 | 2016 | Female | 5 years | Dogon | Tognon | 1 | 2.7 | 3975 | 13568940 | Bandiagara | B | + | case | CM+SMA2 |
| UM4 | 2 | 23 | CM + SMA | 21-Sep-16 | 2016 | Female | 5 years | Dogon | Tognon | 5 | 11.8 | 8025 | 16328224 | Bandiagara | B | + | control | UMC14 |
| BTH5 | 1 | 31 | CM + SMA | 3-Sep-16 | 2016 | Female | 3 years | Dogon | Sogolo | 2 | 4.1 | 71700 | 18234476 | Bandiagara | B | + | case | CM+SMA3 |
| UM5 | 2 | 31 | CM + SMA | 6-Oct-16 | 2016 | Female | 3 years | Dogon | Sogolo | 5 | 6.4 | 2550 | 107624784 | Bandiagara | O | + | control | UMC20 |
| BTH6 | 1 | 32 | CM + SMA | 5-Sep-16 | 2016 | Male | 2 years | Sarakolé | Niamana | 2 | 4.2 | 9800 | 19543612 | HDM-Bamako | O | + | case | CM+SMA4 |
| UM6 | 2 | 32 | CM + SMA | 9-Nov-16 | 2016 | Male | 2 years | Malinke | Niamana | 5 | 5 | 7500 | 24234908 | HDM-Bamako | A | + | control | UMC21 |
| BTH7 | 1 | 37 | CM + SMA | 19-Sep-16 | 2016 | Male | 5 years | Bambara | Yirimadio | 2 | 4.6 | 182000 | 165025144 | HDM-Bamako | B | - | case | CM+SMA5 |
| UM7 | 2 | 37 | CM + SMA | 14-Oct-16 | 2016 | Male | 4 years | Bambara | Yirimadio | 5 | 9.3 | 10800 | 102236756 | HDM-Bamako | O | + | control | UMC24 |
| BTH8 | 1 | 38 | CM + SMA | 23-Oct-16 | 2016 | Male | 3 years | Dogon | Doumbaga | 1 | 2.2 | 1650 | 86831796 | Bandiagara | O | - | case | CM+SMA6 |
| UM8 | 2 | 38 | CM + SMA | 17-Dec-16 | 2016 | Male | 4 years | Dogon | Doumbaga | 5 | 6.4 | 36000 | 26481176 | Bandiagara | A | + | control | UMC25 |

BTH, concurrent cerebral malaria and severe malarial anemia; SMA, severe malarial anemia; CM, cerebral malaria; type; type of participant 1=case; 2=control without history of cerebral malaria; 3=control with history of cerebral malaria; annee, year of enrollment; BCS, Blantyre coma score; GE, parasitemia in parasites per microliter.

| **Table S11. Comprehensive sample characteristics for the comparison of concurrent CM and SMA cases to uncomplicated malaria controls with a history of CM.** | | | | | | | | | | | | | | | | | | |
| --- | --- | --- | --- | --- | --- | --- | --- | --- | --- | --- | --- | --- | --- | --- | --- | --- | --- | --- |
| Name | Type | Match_ID | Match_group | Enrol__date | annee | Sex | Age | Ethnicity | Residency | BCS | Hb__g_dl_ | GE | Total Reads | Site | Blood_type | Rh_factor | Type_cat | De-identified_ID |
| BTH1 | 1 | 6 | CM + SMA | 14-Oct-15 | 2015 | Male | 3 years | Dogon | Guine-wolo | 1 | 2.5 | 525 | 33038818 | Bandiagara | B | + | case | CM+SMA7 |
| UM1 | 3 | 6 | CM + SMA | 12-Nov-15 | 2015 | Male | 3 years | Dogon | Guine-wolo | 5 | 8.2 | 1150 | 81474002 | Bandiagara | B | - | control | UMC30 |
| BTH2 | 1 | 8 | CM + SMA | 23-Oct-15 | 2015 | Male | 4years | Dogon | Kori-Kori | 1 | 2.6 | 950 | 21922384 | Bandiagara | AB | + | case | CM+SMA8 |
| UM2 | 3 | 8 | CM + SMA | 9-Nov-15 | 2015 | Male | 4 years | Dogon | Kori-Kori | 5 | 7.8 | 1425 | 22346636 | Bandiagara | O | + | control | UMC31 |
| BTH3 | 1 | 23 | CM + SMA | 19-Aug-16 | 2016 | Female | 5 years | Dogon | Tognon | 1 | 2.7 | 3975 | 13568940 | Bandiagara | B | + | case | CM+SMA2 |
| UM3 | 3 | 23 | CM + SMA | 21-Sep-16 | 2016 | Female | 5 years | Dogon | Tognon | 5 | 12.1 | 325 | 8154844 | Bandiagara | O | + | control | UMC36 |
| BTH4 | 1 | 31 | CM + SMA | 3-Sep-16 | 2016 | Female | 3 years | Dogon | Sogolo | 2 | 4.1 | 71700 | 18234476 | Bandiagara | B | + | case | CM+SMA3 |
| UM4 | 3 | 31 | CM + SMA | 6-Oct-16 | 2016 | Female | 3 years | Dogon | Sogolo | 5 | 7 | 12950 | 5988748 | Bandiagara | B | + | control | UMC40 |

BTH, concurrent cerebral malaria and severe malarial anemia; SMA, severe malarial anemia; CM, cerebral malaria; type; type of participant 1=case; 2=control without history of cerebral malaria; 3=control with history of cerebral malaria; annee, year of enrollment; BCS, Blantyre coma score; GE, parasitemia in parasites per microliter.

| **Table S12. Wilcoxon signed rank test P-value results for quantitative demographic sample variables.** | | | | | | |
| --- | --- | --- | --- | --- | --- | --- |
|  | CM | | SMA | | Concurrent  CM and SMA | |
|  | Con w/ a History of CM | Con w/o a history of CM | Con w/ a History of CM | Con w/o a history of CM | Con w/ a History of CM | Con w/o a history of CM |
| Parasitemia | 0.016 | ^ | ^ | ^ | ^ | + |
| Blantyre Coma Score | 0.001 | ^ | + | + | ^ | + |
| Hemoglobin Level | 0.47 | ^ | ^ | ^ | ^ | + |

CM, cerebral malaria; SMA, severe malaria anemia. Cells with a “^” indicate comparisons that do not possess a large enough sample size for the distribution of the Wilcoxon W statistic to form a normal distribution and therefore a P-value cannot be confidently determined. However, rejection of the null hypothesis for cells with a “^” can still be achieved through comparison of the W statistic to published critical values. Cells with a “+” indicate that the sample size was not large enough to perform a Wilcoxon signed rank test at an alpha level of 0.05.

| **Table S13. 6-hour (ring-stage) and 24-hour (trophozoite) smoothed 3D7 expression values differentially expressed genes in the comparison of CM cases to uncomplicated malaria controls without a history of CM.** | | | |
| --- | --- | --- | --- |
| Gene | Protein Description | 6-Hour Smoothed 3D7 Value | 24-Hour Smoothed 3D7 Value |
| PF3D7_1322200 | protein STU2, putative | -0.42 | -0.1 |
| PF3D7_0515700 | glideosome-associated protein 40, putative | -0.08 | -2.08 |
| PF3D7_0831300 | Plasmodium exported protein, unknown function | NA | NA |
| PF3D7_1116500 | folate transporter 2 | -0.21 | -0.34 |
| PF3D7_1448400 | ubiquitin-protein ligase, putative | -0.58 | 0.1 |
| PF3D7_0501800 | chromatin assembly factor 1 subunit A | -1.07 | -0.18 |
| PF3D7_1103800 | CCR4-NOT transcription complex subunit NOT1-G, putative | -0.52 | 0.11 |
| PF3D7_0610400 | histone H3 | NA | NA |
| PF3D7_0104400 | 4-hydroxy-3-methylbut-2-enyl diphosphate reductase | -0.22 | 0.03 |
| PF3D7_0529800 | conserved Plasmodium protein, unknown function | NA | NA |
| PF3D7_0310900 | conserved Plasmodium protein, unknown function | -0.17 | -0.19 |
| PF3D7_0914100 | conserved Plasmodium protein, unknown function | 0.33 | -0.67 |
| PF3D7_1327300 | conserved Plasmodium protein, unknown function | 0.04 | -0.62 |
| PF3D7_1429900 | ADP-dependent DNA helicase RecQ | -0.09 | -0.19 |
| PF3D7_1136900 | subtilisin-like protease 2 | 0.14 | -0.97 |
| PF3D7_1228300 | NIMA related kinase 1 | -0.24 | -0.16 |
|  |  | P = 0.65 | |

CM, cerebral malaria.

| **Table S14. 16 significantly differentially expressed genes in the comparison of CM cases to uncomplicated malaria controls without a history of CM.** | | | | |
| --- | --- | --- | --- | --- |
| Gene ID | Protein Product Description | logFC | logCPM | FDR |
| PF3D7_1322200 | protein STU2, putative | 3.551 | 5.207 | 0.001 |
| PF3D7_0515700 | glideosome-associated protein 40, putative | 6.758 | 3.263 | 0.004 |
| PF3D7_0831300 | Plasmodium exported protein, unknown function | 4.173 | 5.069 | 0.005 |
| PF3D7_1116500 | folate transporter 2 | 5.052 | 3.264 | 0.005 |
| PF3D7_1448400 | ubiquitin-protein ligase, putative | 3.185 | 2.651 | 0.009 |
| PF3D7_0501800 | chromatin assembly factor 1 subunit A | 5.841 | 4.483 | 0.009 |
| PF3D7_1103800 | CCR4-NOT transcription complex subunit NOT1-G, putative | 3.350 | 4.633 | 0.024 |
| PF3D7_0610400 | histone H3 | 3.012 | 5.404 | 0.030 |
| PF3D7_0104400 | 4-hydroxy-3-methylbut-2-enyl diphosphate reductase | 4.918 | 3.200 | 0.035 |
| PF3D7_0529800 | conserved Plasmodium protein, unknown function | 3.763 | 3.048 | 0.038 |
| PF3D7_0310900 | conserved Plasmodium protein, unknown function | 3.178 | 1.847 | 0.074 |
| PF3D7_0914100 | conserved Plasmodium protein, unknown function | 4.840 | 2.830 | 0.074 |
| PF3D7_1327300 | conserved Plasmodium protein, unknown function | 2.046 | 6.447 | 0.074 |
| PF3D7_1429900 | ADP-dependent DNA helicase RecQ | 2.146 | 4.440 | 0.084 |
| PF3D7_1136900 | subtilisin-like protease 2 | 5.180 | 3.528 | 0.084 |
| PF3D7_1228300 | NIMA related kinase 1 | 4.419 | 4.104 | 0.087 |

CM, cerebral malaria; logFC, log-fold-change; logCPM, log counts per million; FDR, false discovery rate.

| **Table S15. 120 differentially expressed genes in the comparison of CM cases to uncomplicated malaria controls with a history of CM.** | | | | |
| --- | --- | --- | --- | --- |
| Gene ID | Protein Product Description | logFC | logCPM | FDR |
| PF3D7_1448500 | conserved Plasmodium protein, unknown function | 3**.**222 | 4**.**636 | 2**.**30E-06 |
| PF3D7_0104200 | StAR-related lipid transfer protein | 2**.**576 | 5**.**139 | 2**.**27E-04 |
| PF3D7_1452600 | conserved Plasmodium protein, unknown function | -1**.**487 | 8**.**268 | 2**.**77E-04 |
| PF3D7_0529800 | conserved Plasmodium protein, unknown function | 4**.**144 | 3**.**274 | 3**.**10E-04 |
| PF3D7_1359600 | conserved Plasmodium protein, unknown function | 3**.**868 | 5**.**683 | 9**.**85E-04 |
| PF3D7_0113200 | Plasmodium exported protein, unknown function | 2**.**853 | 6**.**050 | 2**.**01E-03 |
| PF3D7_0113600 | surface-associated interspersed protein 1**.**2 (SURFIN 1**.**2), pseudogene | -3**.**541 | 6**.**879 | 5**.**89E-03 |
| PF3D7_1367800 | secreted ookinete protein, putative | 6**.**123 | 3**.**467 | 6**.**72E-03 |
| PF3D7_1021900 | PHAX domain-containing protein, putative | 2**.**061 | 6**.**638 | 7**.**67E-03 |
| PF3D7_1412800 | glycylpeptide N-tetradecanoyltransferase | -1**.**886 | 8**.**564 | 7**.**67E-03 |
| PF3D7_1239200 | AP2 domain transcription factor, putative | 4**.**743 | 3**.**972 | 7**.**67E-03 |
| PF3D7_1034900 | methionine--tRNA ligase | -1**.**664 | 8**.**186 | 9**.**56E-03 |
| PF3D7_0726100 | Plasmodium exported protein, unknown function | -2**.**319 | 10**.**438 | 9**.**56E-03 |
| PF3D7_0711000 | AAA family ATPase, CDC48 subfamily | 1**.**342 | 6**.**703 | 1**.**37E-02 |
| PF3D7_1453600 | RAP protein, putative | 2**.**421 | 4**.**130 | 1**.**37E-02 |
| PF3D7_0813700 | ABC transporter F family member 1 | 3**.**201 | 4**.**417 | 1**.**53E-02 |
| PF3D7_0530000 | conserved Plasmodium protein, unknown function | -1**.**855 | 6**.**281 | 1**.**53E-02 |
| PF3D7_1412900 | ubiquitin-conjugating enzyme E2, putative | -1**.**462 | 8**.**657 | 1**.**53E-02 |
| PF3D7_1021800 | schizont egress antigen-1 | 6**.**038 | 3**.**811 | 1**.**78E-02 |
| PF3D7_1201100 | RESA-like protein with PHIST and DnaJ domains | 3**.**230 | 6**.**030 | 1**.**78E-02 |
| PF3D7_1208300 | acyl carrier protein, mitochondrial | 2**.**842 | 4**.**123 | 1**.**99E-02 |
| PF3D7_1021700 | VPS13 domain-containing protein, putative | 2**.**790 | 8**.**519 | 2**.**00E-02 |
| PF3D7_1142300 | conserved Plasmodium membrane protein, unknown function | 2**.**834 | 6**.**328 | 2**.**24E-02 |
| PF3D7_0112800 | Plasmodium exported protein (hyp11), unknown function | -3**.**114 | 6**.**225 | 2**.**24E-02 |
| PF3D7_1001200 | acyl-CoA binding protein, isoform 2, ACBP2 | 3**.**757 | 5**.**300 | 2**.**24E-02 |
| PF3D7_1229400 | macrophage migration inhibitory factor | -1**.**559 | 9**.**654 | 2**.**24E-02 |
| PF3D7_1471200 | inorganic anion exchanger, inorganic anion antiporter | 1**.**768 | 5**.**817 | 2**.**24E-02 |
| PF3D7_1244400 | RNA-binding protein, putative | 2**.**913 | 5**.**584 | 2**.**24E-02 |
| PF3D7_0623900 | ribonuclease H2 subunit A, putative | 2**.**987 | 3**.**490 | 2**.**27E-02 |
| PF3D7_1302500 | AAA domain-containing protein, putative | -1**.**631 | 7**.**797 | 2**.**44E-02 |
| PF3D7_1430300 | acid phosphatase, putative | -2**.**342 | 8**.**924 | 2**.**44E-02 |
| PF3D7_1410300 | WD repeat-containing protein, putative | -1**.**178 | 8**.**114 | 2**.**44E-02 |
| PF3D7_0406900 | conserved Plasmodium protein, unknown function | 3**.**019 | 3**.**141 | 2**.**80E-02 |
| PF3D7_0104400 | 4-hydroxy-3-methylbut-2-enyl diphosphate reductase | 3**.**228 | 3**.**645 | 2**.**80E-02 |
| PF3D7_1233600 | asparagine and aspartate rich protein 1 | -1**.**515 | 10**.**739 | 2**.**80E-02 |
| PF3D7_0413900 | ubiquitin carboxyl-terminal hydrolase 13, putative | -1**.**078 | 7**.**305 | 2**.**98E-02 |
| PF3D7_1216400 | conserved Plasmodium membrane protein, unknown function | -1**.**215 | 9**.**490 | 2**.**98E-02 |
| PF3D7_1238900 | protein kinase 2 | 3**.**398 | 6**.**085 | 2**.**98E-02 |
| PF3D7_1204100 | conserved Plasmodium protein, unknown function | 4**.**110 | 2**.**456 | 3**.**26E-02 |
| PF3D7_1303500 | sodium/hydrogen exchanger | -1**.**190 | 8**.**231 | 3**.**43E-02 |
| PF3D7_0513100 | conserved protein, unknown function | -2**.**345 | 4**.**471 | 3**.**53E-02 |
| PF3D7_1232100 | 60 kDa chaperonin | 2**.**843 | 4**.**888 | 3**.**61E-02 |
| PF3D7_1119900 | protein transport protein SEC16, putative | -1**.**125 | 9**.**266 | 3**.**66E-02 |
| PF3D7_0711500 | regulator of chromosome condensation, putative | 2**.**436 | 6**.**109 | 3**.**66E-02 |
| PF3D7_0424400 | surface-associated interspersed protein 4**.**2 (SURFIN 4**.**2) | -3**.**014 | 5**.**694 | 3**.**66E-02 |
| PF3D7_1240000 | 3-hydroxyisobutyryl-CoA hydrolase, putative | -1**.**974 | 5**.**467 | 3**.**82E-02 |
| PF3D7_1428500 | protein kinase, putative | -3**.**026 | 8**.**581 | 3**.**88E-02 |
| PF3D7_1325800 | conserved Plasmodium protein, unknown function | 2**.**452 | 5**.**256 | 3**.**88E-02 |
| PF3D7_0723700 | metallo-hydrolase/oxidoreductase, putative | 2**.**008 | 5**.**403 | 3**.**88E-02 |
| PF3D7_0500800 | mature parasite-infected erythrocyte surface antigen | 4**.**602 | 9**.**131 | 3**.**99E-02 |
| PF3D7_1413200 | conserved Plasmodium protein, unknown function | -3**.**737 | 2**.**467 | 3**.**99E-02 |
| PF3D7_1025900 | conserved protein, unknown function | 3**.**117 | 3**.**261 | 3**.**99E-02 |
| PF3D7_0810800 | hydroxymethyldihydropterin pyrophosphokinase-dihydropteroate synthase | 1**.**922 | 6**.**192 | 3**.**99E-02 |
| PF3D7_0931100 | nucleolar protein Nop52, putative | 0**.**968 | 10**.**000 | 3**.**99E-02 |
| PF3D7_1207700 | 41-3 protein | -3**.**292 | 2**.**397 | 4**.**02E-02 |
| PF3D7_1136300 | tudor staphylococcal nuclease | -1**.**261 | 9**.**742 | 4**.**07E-02 |
| PF3D7_0308500 | activator of Hsp90 ATPase, putative | 3**.**323 | 5**.**353 | 4**.**19E-02 |
| PF3D7_1442400 | protein KIC9 | 2**.**720 | 7**.**476 | 4**.**30E-02 |
| PF3D7_0524600 | 50S ribosomal protein L12, apicoplast, putative | 4**.**591 | 2**.**826 | 4**.**35E-02 |
| PF3D7_1312700 | conserved Plasmodium protein, unknown function | 2**.**946 | 3**.**612 | 4**.**35E-02 |
| PF3D7_0809900 | JmjC domain-containing protein 1, putative | -2**.**128 | 8**.**625 | 4**.**35E-02 |

| PF3D7_0932100 | protein MAM3, putative | -2**.**781 | 6**.**387 | 4**.**35E-02 |
| --- | --- | --- | --- | --- |
| PF3D7_1117500 | tyrosine--tRNA ligase | 4**.**299 | 2**.**910 | 4**.**35E-02 |
| PF3D7_0202400 | translation-enhancing factor | 3**.**646 | 6**.**943 | 4**.**35E-02 |
| PF3D7_1401100 | DnaJ protein, putative | -3**.**067 | 11**.**081 | 4**.**35E-02 |
| PF3D7_0726500 | ubiquitin carboxyl-terminal hydrolase, putative | -0**.**950 | 9**.**825 | 4**.**37E-02 |
| PF3D7_0919000 | nucleosome assembly protein | -1**.**378 | 9**.**932 | 4**.**72E-02 |
| PF3D7_1132400 | conserved Plasmodium membrane protein, unknown function | 2**.**723 | 3**.**468 | 4**.**95E-02 |
| PF3D7_1008500 | protein GPR89, putative | -1**.**078 | 7**.**980 | 4**.**95E-02 |
| PF3D7_0909900 | helicase SKI2W, putative | -1**.**161 | 7**.**096 | 5**.**08E-02 |
| PF3D7_0918700 | conserved Plasmodium protein, unknown function | -3**.**501 | 8**.**752 | 5**.**23E-02 |
| PF3D7_1472200 | histone deacetylase, putative | -1**.**256 | 9**.**946 | 6**.**01E-02 |
| PF3D7_1250600 | translation initiation factor eIF-2B subunit beta, putative | -1**.**492 | 7**.**762 | 6**.**05E-02 |
| PF3D7_0621700 | RAP protein, putative | 1**.**799 | 4**.**535 | 6**.**05E-02 |
| PF3D7_0936300 | ring-exported protein 3 | -1**.**116 | 10**.**336 | 6**.**38E-02 |
| PF3D7_1332600 | DNA-(apurinic or apyrimidinic site) lyase 1 | 2**.**440 | 3**.**292 | 6**.**46E-02 |
| PF3D7_1209200 | U6 snRNA-associated Sm-like protein LSm7, putative | -1**.**164 | 7**.**412 | 6**.**62E-02 |
| PF3D7_0609800 | palmitoyltransferase DHHC2, putative | -3**.**270 | 3**.**914 | 6**.**76E-02 |
| PF3D7_1024000 | conserved Plasmodium protein, unknown function | 3**.**621 | 4**.**731 | 6**.**76E-02 |
| PF3D7_0933900 | conserved Plasmodium protein, unknown function | -1**.**171 | 7**.**195 | 6**.**76E-02 |
| PF3D7_0820900 | conserved Plasmodium protein, unknown function | -1**.**468 | 5**.**648 | 6**.**76E-02 |
| PF3D7_1116100 | serine esterase, putative | 3**.**164 | 3**.**721 | 6**.**76E-02 |
| PF3D7_1102800 | early transcribed membrane protein 11**.**2 | -1**.**829 | 13**.**195 | 6**.**76E-02 |
| PF3D7_0826500 | ubiquitin conjugation factor E4 B, putative | -0**.**925 | 7**.**180 | 6**.**76E-02 |
| PF3D7_0416700 | CDGSH iron-sulfur domain-containing protein, putative | 1**.**369 | 6**.**351 | 6**.**88E-02 |
| PF3D7_0532600 | Plasmodium exported protein, unknown function | 4**.**603 | 6**.**042 | 6**.**88E-02 |
| PF3D7_1418800 | signal recognition particle RNA | 1**.**700 | 7**.**801 | 6**.**88E-02 |
| PF3D7_0730500 | conserved Plasmodium protein, unknown function | 2**.**931 | 2**.**755 | 6**.**88E-02 |
| PF3D7_1016800 | Plasmodium exported protein (PHISTc), unknown function | -2**.**579 | 8**.**035 | 6**.**88E-02 |
| PF3D7_0407100 | methyltransferase, putative | -1**.**743 | 5**.**120 | 6**.**88E-02 |
| PF3D7_1352900 | Plasmodium exported protein, unknown function | -3**.**685 | 7**.**164 | 6**.**88E-02 |
| PF3D7_0403800 | alpha/beta hydrolase, putative | -0**.**961 | 8**.**120 | 7**.**00E-02 |
| PF3D7_1454300 | SNF1-related serine/threonine protein kinase KIN | 3**.**988 | 2**.**573 | 7**.**00E-02 |
| PF3D7_1003700 | MKT1 domain-containing protein, putative | 3**.**044 | 2**.**791 | 7**.**88E-02 |
| PF3D7_1002100 | EMP1-trafficking protein | -2**.**463 | 11**.**220 | 7**.**90E-02 |
| PF3D7_1411800 | conserved Plasmodium protein, unknown function | -1**.**463 | 4**.**463 | 7**.**93E-02 |
| PF3D7_1223900 | 50S ribosomal protein L24, putative | 3**.**000 | 7**.**774 | 7**.**93E-02 |
| PF3D7_1372300 | Plasmodium exported protein (PHIST), unknown function | -2**.**699 | 8**.**767 | 7**.**93E-02 |
| PF3D7_1334300 | MSP7-like protein | 4**.**598 | 4**.**588 | 7**.**93E-02 |
| PF3D7_1430400 | autophagy protein 5, putative | -2**.**484 | 7**.**095 | 8**.**11E-02 |
| PF3D7_0305300 | transporter, putative | -1**.**348 | 9**.**258 | 8**.**11E-02 |
| PF3D7_0710200 | conserved Plasmodium protein, unknown function | 1**.**720 | 5**.**235 | 8**.**89E-02 |
| PF3D7_1246200 | actin I | 1**.**768 | 5**.**827 | 9**.**29E-02 |
| PF3D7_0527600 | conserved Plasmodium protein, unknown function | 2**.**801 | 5**.**662 | 9**.**59E-02 |
| PF3D7_1433500 | DNA topoisomerase 2 | -1**.**543 | 9**.**311 | 9**.**73E-02 |
| PF3D7_1407600 | conserved Plasmodium protein, unknown function | 5**.**411 | 4**.**057 | 9**.**91E-02 |
| PF3D7_0527100 | ubiquitin-conjugating enzyme E2 13 | -1**.**029 | 8**.**023 | 9**.**91E-02 |
| PF3D7_0503400 | actin-depolymerizing factor 1 | 4**.**307 | 4**.**747 | 9**.**91E-02 |
| PF3D7_1433400 | PHD finger protein PHD2, putative | 1**.**401 | 7**.**795 | 9**.**91E-02 |
| PF3D7_0707400 | AAA family ATPase, putative | 1**.**466 | 6**.**487 | 9**.**91E-02 |
| PF3D7_1145400 | dynamin-like protein | -1**.**057 | 6**.**408 | 9**.**91E-02 |
| PF3D7_0422200 | erythrocyte membrane-associated antigen | -1**.**064 | 8**.**049 | 9**.**91E-02 |
| PF3D7_1013500 | phosphoinositide-specific phospholipase C | -1**.**085 | 7**.**726 | 9**.**91E-02 |
| PF3D7_1401200 | Plasmodium exported protein, unknown function | 3**.**166 | 5**.**608 | 9**.**91E-02 |
| PF3D7_0806600 | kinesin-like protein, putative | -1**.**215 | 7**.**155 | 9**.**91E-02 |
| PF3D7_1121100 | conserved protein, unknown function | -1**.**417 | 8**.**032 | 9**.**91E-02 |
| PF3D7_1441100 | conserved Plasmodium protein, unknown function | -1**.**626 | 10**.**202 | 9**.**98E-02 |
| PF3D7_1351100 | conserved protein, unknown function | -1**.**105 | 6**.**520 | 9**.**98E-02 |
| PF3D7_0731800 | alpha/beta hydrolase, putative | -3**.**223 | 7**.**721 | 9**.**98E-02 |
| PF3D7_0102200 | ring-infected erythrocyte surface antigen | -3**.**632 | 13**.**349 | 9**.**98E-02 |

CM, cerebral malaria; logFC, log-fold-change; logCPM, log counts per million; FDR, false discovery rate.

| **Table S16. Singular significantly differentially expressed gene in the comparison of SMA cases to uncomplicated malaria controls without a history of CM.** | | | | |
| --- | --- | --- | --- | --- |
| Gene ID | Protein Product Description | logFC | logCPM | FDR |
| PF3D7_0826100 | HECT-like E3 ubiquitin ligase, putative | 1.533 | 10.276 | 0.017 |

CM, cerebral malaria; SMA, severe malarial anemia; logFC, log-fold-change; logCPM, log counts per million; FDR, false discovery rate.

| **Table S17. Eleven significantly differentially expressed genes in the comparison of SMA cases to uncomplicated malaria controls with a history of CM.** | | | | |
| --- | --- | --- | --- | --- |
| Gene ID | Protein Product Description | logFC | logCPM | FDR |
| PF3D7_1211000 | kinesin-X3, putative | 5.028 | 6.320 | 0.000 |
| PF3D7_1325800 | conserved Plasmodium protein, unknown function | 5.294 | 5.784 | 0.001 |
| PF3D7_0615900 | protein phosphatase, putative | 3.094 | 7.191 | 0.002 |
| PF3D7_1143200 | DnaJ protein, putative | 4.488 | 5.647 | 0.002 |
| PF3D7_0620700 | DnaJ protein, putative | -11.483 | 6.605 | 0.010 |
| PF3D7_0609800 | palmitoyltransferase DHHC2, putative | 5.922 | 3.547 | 0.018 |
| PF3D7_1229300 | PhIL1-interacting candidate PIC1 | 3.231 | 8.230 | 0.033 |
| PF3D7_1111800 | peptidyl-prolyl cis-trans isomerase, putative | 4.442 | 4.058 | 0.069 |
| PF3D7_0526400 | conserved Plasmodium protein, unknown function | -4.804 | 3.759 | 0.069 |
| PF3D7_0220300 | Plasmodium exported protein, unknown function | 2.806 | 6.670 | 0.069 |
| PF3D7_1329400 | AMP deaminase | 4.547 | 3.799 | 0.069 |

CM, cerebral malaria; SMA, severe malarial anemia; logFC, log-fold-change; logCPM, log counts per million; FDR, false discovery rate.

| **Table S18. 524 significantly differentially expressed genes in the comparison of concurrent CM and SMA to uncomplicated malaria controls without a history of CM.** | | | | |
| --- | --- | --- | --- | --- |
| Gene ID | Protein Product Description | logFC | logCPM | FDR |
| PF3D7_0802000 | glutamate dehydrogenase, putative | 4**.**732 | 8**.**807 | 2**.**48E-21 |
| PF3D7_0532400 | lysine-rich membrane-associated PHISTb protein | 4**.**872 | 10**.**456 | 7**.**32E-18 |
| PF3D7_1415400 | Btz domain-containing protein, putative | 3**.**952 | 7**.**438 | 7**.**97E-11 |
| PF3D7_1201200 | Plasmodium exported protein (PHISTa-like), unknown function | -3**.**003 | 8**.**819 | 7**.**97E-11 |
| PF3D7_1033200 | early transcribed membrane protein 10**.**2 | 5**.**030 | 8**.**176 | 7**.**97E-11 |
| PF3D7_0702400 | small exported membrane protein 1 | 3**.**986 | 10**.**059 | 7**.**97E-11 |
| PF3D7_1201000 | Plasmodium exported protein (PHISTb), unknown function | 5**.**982 | 7**.**086 | 2**.**68E-09 |
| PF3D7_1129900 | major facilitator superfamily-related transporter, putative | 3**.**990 | 7**.**407 | 3**.**34E-09 |
| PF3D7_1477500 | Plasmodium exported protein (PHISTb), unknown function | 4**.**152 | 8**.**958 | 2**.**43E-08 |
| PF3D7_0220300 | Plasmodium exported protein, unknown function | 8**.**185 | 5**.**566 | 2**.**49E-07 |
| PF3D7_0817300 | conserved Plasmodium protein, unknown function | 9**.**249 | 10**.**116 | 8**.**04E-07 |
| PF3D7_0935600 | gametocytogenesis-implicated protein | -2**.**410 | 9**.**385 | 1**.**86E-06 |
| PF3D7_1229400 | macrophage migration inhibitory factor | -2**.**293 | 8**.**910 | 1**.**96E-06 |
| PF3D7_0730900 | EMP1-trafficking protein | 3**.**538 | 12**.**203 | 8**.**14E-06 |
| PF3D7_0411000 | AP2 domain transcription factor AP2-Z, putative | 2**.**382 | 9**.**877 | 9**.**45E-06 |
| PF3D7_1401300 | epoxide hydrolase 2 | 5**.**169 | 5**.**787 | 9**.**45E-06 |
| PF3D7_1102900 | Plasmodium exported protein (hyp11), unknown function | -5**.**996 | 5**.**515 | 1**.**04E-05 |
| PF3D7_0711500 | regulator of chromosome condensation, putative | 6**.**020 | 6**.**889 | 1**.**04E-05 |
| PF3D7_1304600 | conserved Plasmodium protein, unknown function | 10**.**600 | 4**.**032 | 1**.**32E-05 |
| PF3D7_1010300 | succinate dehydrogenase subunit 4, putative | -2**.**499 | 9**.**116 | 1**.**61E-05 |
| PF3D7_0115000 | surface-associated interspersed protein 1**.**3 (SURFIN 1**.**3) | 7**.**783 | 5**.**042 | 1**.**68E-05 |
| PF3D7_0201900 | erythrocyte membrane protein 3 | 5**.**281 | 12**.**453 | 1**.**68E-05 |
| PF3D7_0532300 | Plasmodium exported protein (PHISTb), unknown function | 4**.**143 | 10**.**854 | 2**.**24E-05 |
| PF3D7_0731400 | serine/threonine protein kinase, FIKK family, pseudogene | 8**.**421 | 4**.**533 | 2**.**43E-05 |
| PF3D7_1106300 | 5'-3' exoribonuclease 1, putative | 10**.**152 | 3**.**483 | 3**.**22E-05 |
| PF3D7_1102800 | early transcribed membrane protein 11**.**2 | -2**.**454 | 12**.**902 | 3**.**40E-05 |
| PF3D7_0301700 | Plasmodium exported protein, unknown function | 4**.**541 | 8**.**282 | 3**.**40E-05 |
| PF3D7_1131200 | conserved Plasmodium protein, unknown function | 2**.**822 | 7**.**010 | 3**.**73E-05 |
| PF3D7_1149000 | antigen 332, DBL-like protein | 5**.**911 | 10**.**297 | 3**.**83E-05 |
| PF3D7_1469000 | translation initiation factor IF-1 | 8**.**548 | 2**.**524 | 4**.**11E-05 |
| PF3D7_1429400 | rRNA (adenosine-2'-O-)-methyltransferase, putative | 2**.**580 | 7**.**972 | 4**.**30E-05 |
| PF3D7_1478000 | Plasmodium exported protein (PHISTa), unknown function | -2**.**488 | 9**.**034 | 4**.**63E-05 |
| PF3D7_0320400 | oocyst capsule protein Cap380 | 11**.**796 | 3**.**848 | 4**.**63E-05 |
| PF3D7_1039000 | serine/threonine protein kinase, FIKK family | 2**.**812 | 9**.**380 | 5**.**26E-05 |
| PF3D7_1402800 | conserved Plasmodium protein, unknown function | 3**.**661 | 8**.**466 | 5**.**26E-05 |
| PF3D7_1002000 | Plasmodium exported protein (hyp2), unknown function | -3**.**228 | 7**.**267 | 5**.**26E-05 |
| PF3D7_0212800 | multidrug efflux pump, putative | 12**.**448 | 3**.**960 | 6**.**10E-05 |
| PF3D7_0424700 | serine/threonine protein kinase, FIKK family | 4**.**131 | 10**.**874 | 6**.**10E-05 |
| PF3D7_1001800 | Plasmodium exported protein (PHISTc), unknown function | -2**.**662 | 6**.**754 | 7**.**79E-05 |
| PF3D7_0726200 | serine/threonine protein kinase, FIKK family | 4**.**967 | 9**.**003 | 7**.**93E-05 |
| PF3D7_0616800 | malate:quinone oxidoreductase | 5**.**201 | 4**.**872 | 8**.**16E-05 |
| PF3D7_1317200 | AP2 domain transcription factor AP2-FG, putative | 5**.**197 | 7**.**711 | 8**.**16E-05 |
| PF3D7_1238900 | protein kinase 2 | 7**.**244 | 5**.**633 | 8**.**16E-05 |
| PF3D7_0501000 | Plasmodium exported protein, unknown function | 3**.**553 | 8**.**284 | 8**.**86E-05 |
| PF3D7_1358700 | YOP1-like protein, putative | -1**.**978 | 8**.**142 | 1**.**02E-04 |
| PF3D7_0702000 | Plasmodium exported protein (hyp12), unknown function | -2**.**209 | 9**.**452 | 1**.**09E-04 |
| PF3D7_1334300 | MSP7-like protein | 5**.**976 | 4**.**650 | 1**.**18E-04 |
| PF3D7_0202200 | EMP1-trafficking protein | -2**.**155 | 7**.**868 | 1**.**18E-04 |
| PF3D7_0723700 | metallo-hydrolase/oxidoreductase, putative | 4**.**240 | 5**.**880 | 1**.**34E-04 |
| PF3D7_1442400 | protein KIC9 | 3**.**623 | 7**.**401 | 1**.**34E-04 |
| PF3D7_1312900 | eukaryotic translation initation factor 4 gamma | 2**.**074 | 9**.**224 | 1**.**47E-04 |
| PF3D7_0113200 | Plasmodium exported protein, unknown function | 3**.**401 | 5**.**699 | 1**.**60E-04 |
| PF3D7_0501100 | co-chaperone J domain protein JDP | -1**.**741 | 8**.**695 | 1**.**79E-04 |
| PF3D7_1201100 | RESA-like protein with PHIST and DnaJ domains | 6**.**611 | 6**.**658 | 1**.**79E-04 |
| PF3D7_1009600 | protein phosphatase PPM10, putative | 8**.**447 | 5**.**371 | 2**.**10E-04 |
| PF3D7_0202000 | knob-associated histidine-rich protein | 3**.**934 | 13**.**597 | 2**.**14E-04 |
| PF3D7_0202400 | translation-enhancing factor | 5**.**738 | 9**.**711 | 2**.**14E-04 |
| PF3D7_0936800 | Plasmodium exported protein (PHISTc), unknown function | 2**.**564 | 12**.**136 | 2**.**32E-04 |
| PF3D7_0506500 | conserved Plasmodium protein, unknown function | 2**.**516 | 7**.**515 | 2**.**57E-04 |
| PF3D7_0402000 | Plasmodium exported protein (PHISTa), unknown function | 5**.**706 | 8**.**617 | 2**.**73E-04 |
| PF3D7_0214300 | conserved Plasmodium protein, unknown function | 6**.**274 | 6**.**870 | 2**.**73E-04 |
| PF3D7_0718400 | ribosomal protein S8, mitochondrial, putative | 6**.**455 | 5**.**851 | 2**.**81E-04 |
| PF3D7_1337400 | zinc finger protein, putative | -1**.**723 | 7**.**815 | 2**.**92E-04 |
| PF3D7_0800900 | Plasmodium exported protein (hyp7), unknown function, pseudogene | -3**.**941 | 5**.**975 | 2**.**99E-04 |
| PF3D7_0405700 | lysine decarboxylase, putative | 7**.**451 | 4**.**140 | 3**.**14E-04 |
| PF3D7_0305300 | transporter, putative | -1**.**753 | 8**.**708 | 3**.**14E-04 |
| PF3D7_0410000 | erythrocyte vesicle protein 1 | 3**.**071 | 6**.**494 | 3**.**18E-04 |
| PF3D7_0401800 | Plasmodium exported protein (PHISTb), unknown function | 3**.**542 | 7**.**452 | 3**.**28E-04 |
| PF3D7_1103800 | CCR4-NOT transcription complex subunit NOT1-G, putative | 2**.**666 | 5**.**832 | 4**.**59E-04 |

| PF3D7_0621200 | pyridoxine biosynthesis protein PDX1 | 1**.**960 | 8**.**936 | 5**.**51E-04 |
| --- | --- | --- | --- | --- |
| PF3D7_1139300 | AP2 domain transcription factor AP2-G5 | 4**.**394 | 8**.**368 | 6**.**35E-04 |
| PF3D7_0530000 | conserved Plasmodium protein, unknown function | -4**.**195 | 5**.**478 | 6**.**35E-04 |
| PF3D7_0902500 | serine/threonine protein kinase, FIKK family | -2**.**876 | 7**.**609 | 6**.**58E-04 |
| PF3D7_1236200 | conserved Plasmodium protein, unknown function | 5**.**012 | 5**.**012 | 7**.**39E-04 |
| PF3D7_0420300 | AP2 domain transcription factor, putative | 1**.**698 | 9**.**079 | 8**.**27E-04 |
| PF3D7_0621000 | conserved Plasmodium protein, unknown function | 5**.**248 | 4**.**453 | 8**.**62E-04 |
| PF3D7_0831100 | surface-associated interspersed protein 8**.**1 (SURFIN 8**.**1) | 5**.**906 | 5**.**400 | 9**.**33E-04 |
| PF3D7_1464500 | conserved Plasmodium membrane protein, unknown function | 2**.**389 | 7**.**212 | 9**.**33E-04 |
| PF3D7_1013900 | translation initiation factor eIF-2B subunit delta, putative | -1**.**778 | 8**.**391 | 9**.**33E-04 |
| PF3D7_1401100 | DnaJ protein, putative | -2**.**869 | 10**.**424 | 9**.**33E-04 |
| PF3D7_1328500 | alpha/beta-hydrolase, putative | 2**.**923 | 6**.**660 | 9**.**34E-04 |
| PF3D7_1230200 | vacuolar transporter chaperone, putative, pseudogene | 5**.**347 | 4**.**056 | 1**.**00E-03 |
| PF3D7_1415600 | conserved Plasmodium protein, unknown function | 4**.**073 | 5**.**387 | 1**.**12E-03 |
| PF3D7_1401200 | Plasmodium exported protein, unknown function | 4**.**485 | 5**.**656 | 1**.**16E-03 |
| PF3D7_0500800 | mature parasite-infected erythrocyte surface antigen | 6**.**274 | 10**.**340 | 1**.**16E-03 |
| PF3D7_1106600 | DEAD/DEAH box helicase, putative | 5**.**464 | 4**.**356 | 1**.**16E-03 |
| PF3D7_0925800 | regulator of nonsense transcripts 2, putative | 3**.**533 | 5**.**723 | 1**.**16E-03 |
| PF3D7_0711400 | histone deacetylase complex subunit SAP18, putative | 6**.**218 | 3**.**949 | 1**.**20E-03 |
| PF3D7_1452600 | conserved Plasmodium protein, unknown function | -1**.**660 | 8**.**231 | 1**.**20E-03 |
| PF3D7_1004400 | RNA-binding protein, putative | 3**.**564 | 5**.**427 | 1**.**25E-03 |
| PF3D7_0220500 | Plasmodium exported protein (hyp2), unknown function | 4**.**099 | 6**.**824 | 1**.**25E-03 |
| PF3D7_0627500 | protein DJ-1 | -1**.**668 | 8**.**722 | 1**.**26E-03 |
| PF3D7_1440800 | major facilitator superfamily domain-containing protein, putative | 4**.**877 | 3**.**919 | 1**.**27E-03 |
| PF3D7_1436300 | translocon component PTEX150 | 1**.**768 | 11**.**312 | 1**.**27E-03 |
| PF3D7_0507900 | conserved Plasmodium protein, unknown function | -2**.**693 | 6**.**338 | 1**.**33E-03 |
| PF3D7_1351000 | phosphatidylinositol transfer protein, putative | 1**.**655 | 8**.**661 | 1**.**33E-03 |
| PF3D7_1016800 | Plasmodium exported protein (PHISTc), unknown function | -2**.**402 | 7**.**631 | 1**.**35E-03 |
| PF3D7_0614300 | major facilitator superfamily-related transporter, putative | 1**.**685 | 8**.**166 | 1**.**49E-03 |
| PF3D7_1344300 | zinc finger protein, putative | 5**.**226 | 5**.**648 | 1**.**49E-03 |
| PF3D7_1477900 | acyl-CoA synthetase, pseudogene | 8**.**018 | 3**.**202 | 1**.**49E-03 |
| PF3D7_0424800 | Plasmodium exported protein (PHISTb), unknown function | -4**.**037 | 6**.**596 | 1**.**57E-03 |
| PF3D7_1219000 | formin 2 | 3**.**877 | 9**.**099 | 1**.**67E-03 |
| PF3D7_1035800 | probable protein, unknown function | 6**.**649 | 8**.**126 | 1**.**67E-03 |
| PF3D7_0831400 | Plasmodium exported protein, unknown function | 5**.**678 | 6**.**162 | 1**.**74E-03 |
| PF3D7_0906000 | exoribonuclease II | 3**.**697 | 4**.**862 | 1**.**75E-03 |
| PF3D7_0220600 | Plasmodium exported protein (hyp9), unknown function | 5**.**209 | 8**.**161 | 1**.**92E-03 |
| PF3D7_1031300 | SAE2 domain-containing protein, putative | 3**.**929 | 6**.**340 | 1**.**92E-03 |
| PF3D7_0703200 | conserved Plasmodium protein, unknown function | 4**.**119 | 5**.**781 | 2**.**07E-03 |
| PF3D7_0519500 | CCR4 domain-containing protein 1, putative | 2**.**555 | 7**.**494 | 2**.**08E-03 |
| PF3D7_1236100 | clustered-asparagine-rich protein | 3**.**517 | 8**.**244 | 2**.**08E-03 |
| PF3D7_1234200 | helicase, putative, pseudogene | 4**.**114 | 4**.**732 | 2**.**19E-03 |
| PF3D7_0404600 | conserved Plasmodium membrane protein, unknown function | 4**.**204 | 9**.**252 | 2**.**19E-03 |
| PF3D7_1463500 | fam-a protein | -3**.**701 | 5**.**710 | 2**.**20E-03 |
| PF3D7_0306300 | glutaredoxin 1 | -2**.**434 | 7**.**457 | 2**.**22E-03 |
| PF3D7_0617100 | AP-2 complex subunit alpha, putative | -1**.**548 | 7**.**959 | 2**.**38E-03 |
| PF3D7_1337500 | conserved Plasmodium protein, unknown function | -1**.**906 | 8**.**634 | 2**.**40E-03 |
| PF3D7_1471900 | conserved Plasmodium protein, unknown function | -1**.**949 | 6**.**351 | 2**.**44E-03 |
| PF3D7_1447900 | multidrug resistance protein 2 | 4**.**799 | 3**.**988 | 2**.**51E-03 |
| PF3D7_0731300 | Plasmodium exported protein (PHISTb), unknown function | 4**.**549 | 8**.**775 | 2**.**54E-03 |
| PF3D7_1438400 | metacaspase-2 | 2**.**669 | 6**.**798 | 2**.**54E-03 |
| PF3D7_0916200 | mitochondrial ribonuclease P catalytic subunit, putative | 3**.**459 | 5**.**503 | 2**.**57E-03 |
| PF3D7_1414400 | serine/threonine protein phosphatase PP1 | 4**.**607 | 4**.**620 | 2**.**59E-03 |
| PF3D7_1022000 | RNA-binding protein UIS12, putative | 2**.**188 | 6**.**810 | 2**.**70E-03 |
| PF3D7_1440500 | allantoicase, putative | 5**.**184 | 5**.**168 | 2**.**83E-03 |
| PF3D7_0403800 | alpha/beta hydrolase, putative | -1**.**583 | 7**.**670 | 2**.**87E-03 |
| PF3D7_0113300 | Plasmodium exported protein (hyp1), unknown function | -2**.**084 | 7**.**005 | 3**.**03E-03 |
| PF3D7_0114500 | Plasmodium exported protein (hyp10), unknown function | 4**.**580 | 6**.**014 | 3**.**22E-03 |
| PF3D7_1301500 | Plasmodium exported protein (PHISTa), unknown function | 5**.**758 | 2**.**256 | 3**.**30E-03 |
| PF3D7_0918700 | conserved Plasmodium protein, unknown function | -2**.**232 | 8**.**246 | 3**.**32E-03 |
| PF3D7_0804800 | peptidyl-prolyl cis-trans isomerase | -1**.**564 | 7**.**723 | 3**.**32E-03 |
| PF3D7_1325800 | conserved Plasmodium protein, unknown function | 3**.**211 | 5**.**358 | 3**.**32E-03 |
| PF3D7_1342900 | AP2 domain transcription factor AP2-HS | 1**.**906 | 7**.**760 | 3**.**38E-03 |
| PF3D7_0418400 | LSM domain-containing protein, putative | 2**.**524 | 5**.**769 | 3**.**48E-03 |
| PF3D7_0729100 | apicomplexan kinetochore protein 8, putative | 3**.**226 | 6**.**343 | 3**.**49E-03 |
| PF3D7_0311500 | conserved protein, unknown function | -2**.**741 | 6**.**210 | 3**.**61E-03 |
| PF3D7_1015600 | heat shock protein 60 | 4**.**722 | 5**.**187 | 3**.**81E-03 |
| PF3D7_1018600 | tRNA wybutosine-synthesizing protein, putative | -1**.**434 | 8**.**492 | 3**.**96E-03 |
| PF3D7_0516000 | RAP protein, putative | 6**.**666 | 4**.**427 | 3**.**99E-03 |
| PF3D7_0532500 | Plasmodium exported protein, unknown function | 7**.**127 | 3**.**588 | 4**.**13E-03 |

| PF3D7_1351100 | conserved protein, unknown function | -2**.**624 | 5**.**931 | 4**.**15E-03 |
| --- | --- | --- | --- | --- |
| PF3D7_0309500 | asparagine synthetase [glutamine-hydrolyzing], putative | -2**.**683 | 8**.**286 | 4**.**16E-03 |
| PF3D7_1031600 | protein GEXP15 | 1**.**648 | 7**.**344 | 4**.**35E-03 |
| PF3D7_1433400 | PHD finger protein PHD2, putative | 2**.**305 | 8**.**328 | 4**.**35E-03 |
| PF3D7_0201500 | Plasmodium exported protein (hyp9), unknown function | 4**.**989 | 8**.**021 | 4**.**35E-03 |
| PF3D7_1148700 | Plasmodium exported protein (PHISTc), unknown function | 2**.**972 | 9**.**339 | 4**.**35E-03 |
| PF3D7_1102700 | early transcribed membrane protein 11**.**1 | -1**.**458 | 11**.**221 | 4**.**35E-03 |
| PF3D7_0830900 | Plasmodium exported protein, unknown function | 6**.**011 | 6**.**319 | 4**.**48E-03 |
| PF3D7_0201600 | PHISTb domain-containing RESA-like protein 1 | 3**.**171 | 8**.**389 | 4**.**58E-03 |
| PF3D7_1001500 | early transcribed membrane protein 10**.**1 | -1**.**860 | 11**.**450 | 4**.**87E-03 |
| PF3D7_0315200 | circumsporozoite- and TRAP-related protein | 8**.**208 | 4**.**876 | 4**.**92E-03 |
| PF3D7_1315400 | zinc finger (CCCH type) protein, putative | -1**.**456 | 8**.**747 | 4**.**92E-03 |
| PF3D7_0830600 | Plasmodium exported protein (PHISTc), unknown function | -1**.**735 | 8**.**660 | 5**.**03E-03 |
| PF3D7_1112700 | conserved Plasmodium protein, unknown function | 7**.**152 | 3**.**365 | 5**.**15E-03 |
| PF3D7_1203500 | threonylcarbamoyl-AMP synthase, putative | -1**.**574 | 6**.**990 | 5**.**25E-03 |
| PF3D7_1208900 | protein phosphatase PPM11, putative | 2**.**185 | 6**.**196 | 5**.**25E-03 |
| PF3D7_0510500 | topoisomerase I | 6**.**858 | 6**.**192 | 5**.**30E-03 |
| PF3D7_1412300 | nuclear transport factor 2, putative | -1**.**719 | 7**.**864 | 5**.**64E-03 |
| PF3D7_1410500 | conserved Plasmodium protein, unknown function | -10**.**101 | 1**.**422 | 5**.**96E-03 |
| PF3D7_1321500 | 3',5'-cyclic nucleotide phosphodiesterase beta | 5**.**811 | 3**.**738 | 6**.**12E-03 |
| PF3D7_1301400 | Plasmodium exported protein (hyp12), unknown function | 4**.**042 | 10**.**137 | 6**.**24E-03 |
| PF3D7_0823800 | DnaJ protein, putative | 3**.**455 | 4**.**423 | 6**.**74E-03 |
| PF3D7_1001700 | Plasmodium exported protein (PHISTc), unknown function | -1**.**611 | 8**.**295 | 7**.**08E-03 |
| PF3D7_1218500 | dynamin-like protein, putative | -1**.**762 | 7**.**998 | 7**.**08E-03 |
| PF3D7_0301400 | Plasmodium exported protein, unknown function | 8**.**032 | 4**.**164 | 7**.**38E-03 |
| PF3D7_0526600 | conserved Plasmodium protein, unknown function | 4**.**369 | 6**.**761 | 7**.**74E-03 |
| PF3D7_1372000 | Plasmodium exported protein (PHISTa), unknown function | 11**.**573 | 6**.**343 | 7**.**78E-03 |
| PF3D7_1340600 | RNA lariat debranching enzyme, putative | 2**.**896 | 5**.**779 | 7**.**78E-03 |
| PF3D7_0922600 | glutamine synthetase, putative | -1**.**454 | 8**.**318 | 7**.**93E-03 |
| PF3D7_1329100 | myosin F, putative | 2**.**990 | 7**.**364 | 7**.**93E-03 |
| PF3D7_1136900 | subtilisin-like protease 2 | 6**.**025 | 4**.**545 | 8**.**06E-03 |
| PF3D7_0826300 | SPRY domain, putative | 5**.**026 | 3**.**500 | 8**.**60E-03 |
| PF3D7_0525000 | zinc finger protein, putative | 4**.**741 | 7**.**589 | 8**.**72E-03 |
| PF3D7_0513300 | purine nucleoside phosphorylase | -1**.**601 | 8**.**824 | 8**.**78E-03 |
| PF3D7_1121100 | conserved protein, unknown function | -1**.**499 | 7**.**707 | 8**.**78E-03 |
| PF3D7_1353300 | conserved Plasmodium protein, unknown function | 2**.**380 | 6**.**026 | 8**.**79E-03 |
| PF3D7_0702200 | lysophospholipase LPL20 | 1**.**651 | 7**.**810 | 8**.**79E-03 |
| PF3D7_1409300 | DNA damage-inducible protein 1 | 6**.**785 | 3**.**720 | 8**.**95E-03 |
| PF3D7_1022400 | serine/arginine-rich splicing factor 4 | 1**.**826 | 8**.**709 | 9**.**02E-03 |
| PF3D7_0308500 | activator of Hsp90 ATPase, putative | 4**.**920 | 5**.**025 | 9**.**29E-03 |
| PF3D7_1131800 | oxysterol-binding protein, putative | 2**.**756 | 5**.**564 | 9**.**55E-03 |
| PF3D7_1134000 | heat shock protein 70 | 1**.**951 | 6**.**933 | 9**.**55E-03 |
| PF3D7_0608800 | ornithine aminotransferase | 3**.**928 | 7**.**474 | 9**.**55E-03 |
| PF3D7_1357900 | pyrroline-5-carboxylate reductase, putative | 4**.**908 | 4**.**693 | 9**.**75E-03 |
| PF3D7_1025100 | glutamine--fructose-6-phosphate aminotransferase [isomerizing], putative | -1**.**639 | 7**.**605 | 9**.**81E-03 |
| PF3D7_1203200 | signal recognition particle subunit SRP14 | -2**.**034 | 7**.**461 | 1**.**09E-02 |
| PF3D7_0831000 | Plasmodium exported protein (PHISTb), unknown function | 7**.**950 | 3**.**764 | 1**.**09E-02 |
| PF3D7_1107800 | AP2 domain transcription factor, putative | 5**.**117 | 8**.**674 | 1**.**10E-02 |
| PF3D7_0102600 | serine/threonine protein kinase, FIKK family | 4**.**643 | 7**.**066 | 1**.**15E-02 |
| PF3D7_1352500 | thioredoxin-related protein, putative | 1**.**722 | 6**.**439 | 1**.**15E-02 |
| PF3D7_1475900 | KELT protein | 6**.**505 | 3**.**916 | 1**.**15E-02 |
| PF3D7_1417600 | ookinete surface and oocyst capsule protein OSCP, putative | 1**.**337 | 9**.**876 | 1**.**16E-02 |
| PF3D7_1209300 | telomere repeat-binding zinc finger protein | 6**.**630 | 5**.**880 | 1**.**18E-02 |
| PF3D7_0830800 | surface-associated interspersed protein 8**.**2 (SURFIN 8**.**2) | 3**.**664 | 6**.**043 | 1**.**21E-02 |
| PF3D7_0532600 | Plasmodium exported protein, unknown function | 5**.**560 | 5**.**647 | 1**.**21E-02 |
| PF3D7_0114800 | Plasmodium exported protein (hyp7), unknown function | 8**.**151 | 3**.**295 | 1**.**22E-02 |
| PF3D7_0417800 | cdc2-related protein kinase 1 | 4**.**072 | 4**.**595 | 1**.**22E-02 |
| PF3D7_1215100 | SUN domain-containing protein, putative | 2**.**961 | 5**.**207 | 1**.**22E-02 |
| PF3D7_0923400 | conserved Plasmodium protein, unknown function | 2**.**537 | 5**.**923 | 1**.**22E-02 |
| PF3D7_0902700 | Plasmodium exported protein (PHISTb), unknown function, pseudogene | 9**.**126 | 5**.**798 | 1**.**23E-02 |
| PF3D7_1112100 | conserved Plasmodium protein, unknown function | 3**.**369 | 5**.**481 | 1**.**25E-02 |
| PF3D7_1323500 | plasmepsin V | 2**.**685 | 7**.**097 | 1**.**26E-02 |
| PF3D7_0629900 | CRAL/TRIO domain-containing protein, putative | -1**.**560 | 7**.**550 | 1**.**29E-02 |
| PF3D7_0613700 | syntaxin-binding protein, putative | 3**.**188 | 5**.**206 | 1**.**30E-02 |
| PF3D7_0921000 | ubiquitin-conjugating enzyme E2, putative | -2**.**277 | 6**.**756 | 1**.**35E-02 |
| PF3D7_0213700 | conserved protein, unknown function | 2**.**009 | 5**.**833 | 1**.**35E-02 |
| PF3D7_0506700 | GTPase-activating protein, putative | 3**.**498 | 6**.**386 | 1**.**38E-02 |
| PF3D7_1327300 | conserved Plasmodium protein, unknown function | 4**.**311 | 7**.**623 | 1**.**41E-02 |
| PF3D7_0726100 | Plasmodium exported protein, unknown function | -1**.**566 | 9**.**322 | 1**.**41E-02 |
| PF3D7_1308300 | 40S ribosomal protein S27 | -1**.**376 | 9**.**009 | 1**.**42E-02 |

| PF3D7_0108300 | conserved Plasmodium protein, unknown function | 1**.**383 | 10**.**179 | 1**.**42E-02 |
| --- | --- | --- | --- | --- |
| PF3D7_1465900 | 40S ribosomal protein S3 | -1**.**252 | 10**.**255 | 1**.**45E-02 |
| PF3D7_0810500 | protein phosphatase PPM7, putative | -2**.**119 | 5**.**718 | 1**.**46E-02 |
| PF3D7_1252900 | Plasmodium exported protein, unknown function | -3**.**844 | 5**.**023 | 1**.**46E-02 |
| PF3D7_0314100 | vesicle transport v-SNARE protein, putative | -2**.**328 | 5**.**488 | 1**.**47E-02 |
| PF3D7_1130500 | conserved oligomeric Golgi complex subunit 2, putative | 5**.**636 | 3**.**484 | 1**.**48E-02 |
| PF3D7_0911400 | conserved Plasmodium protein, unknown function | 4**.**552 | 4**.**178 | 1**.**48E-02 |
| PF3D7_1226300 | haloacid dehalogenase-like hydrolase, putative | -1**.**384 | 7**.**592 | 1**.**52E-02 |
| PF3D7_1250600 | translation initiation factor eIF-2B subunit beta, putative | -1**.**925 | 7**.**121 | 1**.**52E-02 |
| PF3D7_1014700 | prohibitin 2, putative | 8**.**543 | 3**.**917 | 1**.**56E-02 |
| PF3D7_0615900 | protein phosphatase, putative | 5**.**805 | 5**.**236 | 1**.**58E-02 |
| PF3D7_1109400 | essential nuclear protein 1, putative | -1**.**278 | 8**.**962 | 1**.**59E-02 |
| PF3D7_0519400 | 40S ribosomal protein S24 | -1**.**578 | 10**.**464 | 1**.**64E-02 |
| PF3D7_1232100 | 60 kDa chaperonin | 3**.**319 | 5**.**277 | 1**.**64E-02 |
| PF3D7_0601900 | conserved Plasmodium protein, unknown function | 3**.**342 | 10**.**419 | 1**.**66E-02 |
| PF3D7_0301200 | serine/threonine protein kinase, FIKK family | -3**.**289 | 6**.**443 | 1**.**67E-02 |
| PF3D7_1116300 | peptidyl-prolyl cis-trans isomerase | -1**.**521 | 7**.**096 | 1**.**77E-02 |
| PF3D7_1352000 | GTP-binding protein, putative | 3**.**594 | 5**.**427 | 1**.**77E-02 |
| PF3D7_1477000 | Plasmodium exported protein (hyp17), unknown function, pseudogene | 3**.**045 | 6**.**728 | 1**.**78E-02 |
| PF3D7_0917900 | heat shock protein 70 | 1**.**225 | 10**.**192 | 1**.**79E-02 |
| PF3D7_0316700 | protein YOP1, putative | 2**.**670 | 4**.**940 | 1**.**81E-02 |
| PF3D7_1436000 | glucose-6-phosphate isomerase | 2**.**094 | 7**.**657 | 1**.**83E-02 |
| PF3D7_1246800 | signal recognition particle receptor subunit beta, putative | 4**.**833 | 3**.**538 | 1**.**87E-02 |
| PF3D7_1032500 | DER1-like protein, putative | 4**.**613 | 4**.**452 | 1**.**87E-02 |
| PF3D7_1239200 | AP2 domain transcription factor, putative | 3**.**969 | 6**.**455 | 1**.**91E-02 |
| PF3D7_0110000 | conserved Plasmodium protein, unknown function | -4**.**763 | 3**.**915 | 1**.**94E-02 |
| PF3D7_1136500 | casein kinase 1 | -1**.**362 | 8**.**591 | 1**.**95E-02 |
| PF3D7_1250900 | conserved protein, unknown function | 4**.**662 | 3**.**042 | 1**.**96E-02 |
| PF3D7_0217800 | 40S ribosomal protein S26 | -1**.**477 | 9**.**582 | 1**.**98E-02 |
| PF3D7_0933900 | conserved Plasmodium protein, unknown function | -1**.**462 | 6**.**771 | 2**.**00E-02 |
| PF3D7_1439900 | triosephosphate isomerase | -1**.**282 | 9**.**078 | 2**.**03E-02 |
| PF3D7_0501800 | chromatin assembly factor 1 subunit A | 4**.**233 | 5**.**343 | 2**.**03E-02 |
| PF3D7_0113000 | glutamic acid-rich protein GARP | 1**.**655 | 10**.**748 | 2**.**07E-02 |
| PF3D7_1347700 | ethanolamine-phosphate cytidylyltransferase | -1**.**267 | 8**.**950 | 2**.**11E-02 |
| PF3D7_1026000 | conserved Plasmodium protein, unknown function | 3**.**382 | 5**.**717 | 2**.**21E-02 |
| PF3D7_1142300 | conserved Plasmodium membrane protein, unknown function | 3**.**894 | 5**.**967 | 2**.**30E-02 |
| PF3D7_1402500 | ribosomal protein S27a, putative | -1**.**414 | 8**.**927 | 2**.**35E-02 |
| PF3D7_0603400 | trophozoite exported protein 1 | 2**.**083 | 6**.**551 | 2**.**35E-02 |
| PF3D7_1114200 | GTPase-activating protein, putative | -1**.**727 | 7**.**294 | 2**.**35E-02 |
| PF3D7_0817100 | tRNA modification GTPase, putative | 8**.**459 | 3**.**527 | 2**.**37E-02 |
| PF3D7_0504700 | centrosomal protein CEP120, putative | 6**.**219 | 5**.**708 | 2**.**40E-02 |
| PF3D7_1353100 | Plasmodium exported protein, unknown function | 1**.**397 | 8**.**498 | 2**.**45E-02 |
| PF3D7_0301500 | Plasmodium exported protein, unknown function | -2**.**412 | 4**.**227 | 2**.**45E-02 |
| PF3D7_0303100 | CLP1 P-loop domain-containing protein, putative | 3**.**441 | 3**.**391 | 2**.**46E-02 |
| PF3D7_1401500 | esterase, putative | 4**.**434 | 6**.**912 | 2**.**47E-02 |
| PF3D7_1239700 | ATP-dependent zinc metalloprotease FTSH 1 | 3**.**266 | 5**.**795 | 2**.**47E-02 |
| PF3D7_1200700 | acyl-CoA synthetase | -1**.**710 | 11**.**437 | 2**.**47E-02 |
| PF3D7_0520600 | bis(5'-nucleosyl)-tetraphosphatase [asymmetrical] | -2**.**338 | 6**.**112 | 2**.**47E-02 |
| PF3D7_1438900 | thioredoxin peroxidase 1 | -1**.**364 | 8**.**251 | 2**.**47E-02 |
| PF3D7_1367800 | secreted ookinete protein, putative | 5**.**826 | 4**.**618 | 2**.**47E-02 |
| PF3D7_1440200 | stromal-processing peptidase, putative | 3**.**544 | 4**.**877 | 2**.**47E-02 |
| PF3D7_1406600 | ATP-dependent Clp protease regulatory subunit ClpC | 5**.**558 | 3**.**363 | 2**.**47E-02 |
| PF3D7_1440700 | AP-3 complex subunit mu, putative | 3**.**407 | 4**.**757 | 2**.**47E-02 |
| PF3D7_0936000 | ring-exported protein 2 | -1**.**194 | 9**.**097 | 2**.**47E-02 |
| PF3D7_0521300 | zinc finger protein, putative | -1**.**382 | 6**.**994 | 2**.**47E-02 |
| PF3D7_1252300 | conserved Plasmodium protein, unknown function | 3**.**217 | 9**.**903 | 2**.**52E-02 |
| PF3D7_1368700 | mitochondrial carrier protein, putative | 6**.**472 | 2**.**640 | 2**.**52E-02 |
| PF3D7_1016500 | Plasmodium exported protein (PHISTc), unknown function | 4**.**443 | 3**.**933 | 2**.**55E-02 |
| PF3D7_1311900 | V-type proton ATPase catalytic subunit A | 1**.**304 | 7**.**489 | 2**.**56E-02 |
| PF3D7_1021900 | PHAX domain-containing protein, putative | 2**.**117 | 6**.**921 | 2**.**57E-02 |
| PF3D7_1149400 | Plasmodium exported protein, unknown function | 6**.**242 | 4**.**681 | 2**.**58E-02 |
| PF3D7_0702300 | sporozoite threonine and asparagine-rich protein | -1**.**571 | 8**.**124 | 2**.**61E-02 |
| PF3D7_1028500 | partial CSTF domain-containing protein, putative | -1**.**424 | 6**.**807 | 2**.**63E-02 |
| PF3D7_0903200 | ras-related protein RAB7 | 4**.**526 | 5**.**095 | 2**.**63E-02 |
| PF3D7_1433500 | DNA topoisomerase 2 | -1**.**610 | 8**.**688 | 2**.**66E-02 |
| PF3D7_0205500 | DNA-directed RNA polymerase II 16 kDa subunit, putative | -1**.**639 | 6**.**758 | 2**.**70E-02 |
| PF3D7_1138700 | protein KIC5 | -1**.**183 | 8**.**464 | 2**.**73E-02 |
| PF3D7_1359800 | ADP-ribosylation factor, putative | -5**.**056 | 4**.**149 | 2**.**74E-02 |
| PF3D7_1412800 | glycylpeptide N-tetradecanoyltransferase | -1**.**585 | 8**.**066 | 2**.**74E-02 |
| PF3D7_1418800 | signal recognition particle RNA | 4**.**095 | 13**.**082 | 2**.**76E-02 |

| PF3D7_1478600 | EMP1-trafficking protein | 4**.**273 | 10**.**337 | 2**.**76E-02 |
| --- | --- | --- | --- | --- |
| PF3D7_1454300 | SNF1-related serine/threonine protein kinase KIN | 6**.**084 | 3**.**812 | 2**.**79E-02 |
| PF3D7_1361100 | protein transport protein Sec24A | -1**.**568 | 8**.**478 | 2**.**83E-02 |
| PF3D7_0907100 | conserved Plasmodium protein, unknown function | 4**.**709 | 2**.**851 | 2**.**84E-02 |
| PF3D7_0320500 | nicotinamidase, putative | 3**.**523 | 6**.**225 | 2**.**86E-02 |
| PF3D7_1323400 | 60S ribosomal protein L23 | -1**.**274 | 9**.**986 | 2**.**89E-02 |
| PF3D7_0611700 | 60S ribosomal protein L39 | -1**.**617 | 8**.**239 | 2**.**90E-02 |
| PF3D7_1353700 | reactive oxygen species modulator 1, putative | 6**.**846 | 3**.**227 | 2**.**94E-02 |
| PF3D7_1422200 | conserved Plasmodium protein, unknown function | -5**.**471 | 1**.**897 | 2**.**94E-02 |
| PF3D7_0906910 | tubulin-specific chaperone, putative | 1**.**969 | 5**.**543 | 2**.**94E-02 |
| PF3D7_0111800 | eukaryotic translation initiation factor 4E, putative | 2**.**003 | 5**.**626 | 2**.**94E-02 |
| PF3D7_1203000 | origin recognition complex subunit 1 | 5**.**531 | 5**.**170 | 2**.**94E-02 |
| PF3D7_0604600 | DNA helicase, putative | 3**.**945 | 4**.**146 | 2**.**94E-02 |
| PF3D7_0726900 | mitochondrial import inner membrane translocase subunit TIM50, putative | 3**.**191 | 4**.**281 | 2**.**94E-02 |
| PF3D7_1025000 | Eps15-like protein | -1**.**434 | 9**.**020 | 2**.**95E-02 |
| PF3D7_0935700 | Plasmodium exported protein, unknown function | -1**.**571 | 7**.**304 | 2**.**95E-02 |
| PF3D7_0110500 | bromodomain protein 3, putative | 1**.**248 | 7**.**908 | 2**.**95E-02 |
| PF3D7_0809900 | JmjC domain-containing protein 1, putative | -1**.**756 | 8**.**179 | 2**.**95E-02 |
| PF3D7_1371600 | erythrocyte binding like protein 1, pseudogene | 5**.**854 | 4**.**374 | 2**.**99E-02 |
| PF3D7_1149200 | ring-infected erythrocyte surface antigen | -2**.**148 | 10**.**604 | 3**.**22E-02 |
| PF3D7_0522100 | conserved Plasmodium protein, unknown function | 4**.**056 | 2**.**896 | 3**.**37E-02 |
| PF3D7_0423700 | early transcribed membrane protein 4 | 3**.**992 | 5**.**203 | 3**.**42E-02 |
| PF3D7_1412100 | mini-chromosome maintenance complex-binding protein | 5**.**734 | 3**.**455 | 3**.**43E-02 |
| PF3D7_1372300 | Plasmodium exported protein (PHIST), unknown function | -1**.**726 | 7**.**283 | 3**.**45E-02 |
| PF3D7_0616000 | pyridoxal kinase | 5**.**738 | 5**.**569 | 3**.**46E-02 |
| PF3D7_0800700 | surface-associated interspersed protein 8**.**3 (SURFIN 8**.**3) | 4**.**536 | 5**.**095 | 3**.**46E-02 |
| PF3D7_1103400 | iron-sulfur cluster assembly protein SufD | 4**.**869 | 4**.**352 | 3**.**52E-02 |
| PF3D7_1002100 | EMP1-trafficking protein | -2**.**397 | 10**.**229 | 3**.**52E-02 |
| PF3D7_1104700 | DNA-directed RNA polymerase III subunit RPC8, putative | 2**.**696 | 5**.**418 | 3**.**52E-02 |
| PF3D7_1434700 | mitochondrial import inner membrane translocase subunit TIM17, putative | 2**.**035 | 6**.**173 | 3**.**58E-02 |
| PF3D7_0918100 | cytochrome b5-like heme/steroid binding protein, putative | -2**.**352 | 7**.**414 | 3**.**67E-02 |
| PF3D7_0205600 | conserved Plasmodium protein, unknown function | 5**.**263 | 2**.**624 | 3**.**67E-02 |
| PF3D7_0102200 | ring-infected erythrocyte surface antigen | -1**.**978 | 12**.**377 | 3**.**87E-02 |
| PF3D7_1237700 | conserved protein, unknown function | -1**.**661 | 6**.**295 | 3**.**88E-02 |
| PF3D7_1370300 | membrane associated histidine-rich protein 1 | 1**.**837 | 11**.**187 | 3**.**91E-02 |
| PF3D7_0309800 | YTH domain-containing protein 2 | -1**.**664 | 6**.**095 | 4**.**03E-02 |
| PF3D7_0219700 | Plasmodium exported protein (PHISTc), unknown function | -1**.**917 | 6**.**022 | 4**.**08E-02 |
| PF3D7_0713800 | negative elongation factor A, putative | 3**.**913 | 1**.**890 | 4**.**08E-02 |
| PF3D7_1013200 | conserved Plasmodium protein, unknown function | 2**.**929 | 6**.**461 | 4**.**08E-02 |
| PF3D7_0202500 | early transcribed membrane protein 2 | -1**.**208 | 10**.**531 | 4**.**12E-02 |
| PF3D7_0508000 | 6-cysteine protein | -4**.**356 | 5**.**298 | 4**.**12E-02 |
| PF3D7_0204300 | conserved Plasmodium protein, unknown function | -1**.**789 | 6**.**391 | 4**.**12E-02 |
| PF3D7_0217200 | conserved Plasmodium protein, unknown function | 5**.**799 | 2**.**804 | 4**.**12E-02 |
| PF3D7_0618700 | trafficking protein particle complex subunit 6A, putative | -2**.**623 | 5**.**167 | 4**.**12E-02 |
| PF3D7_1445400 | protein serine/threonine kinase-1 | 1**.**250 | 9**.**021 | 4**.**16E-02 |
| PF3D7_1320500 | SNARE protein, putative | 4**.**164 | 4**.**061 | 4**.**24E-02 |
| PF3D7_0102500 | erythrocyte binding antigen-181 | 5**.**510 | 4**.**377 | 4**.**27E-02 |
| PF3D7_0402300 | reticulocyte binding protein homologue 1 | 6**.**817 | 4**.**406 | 4**.**27E-02 |
| PF3D7_1343800 | VPS13 domain-containing protein, putative | 2**.**428 | 5**.**787 | 4**.**33E-02 |
| PF3D7_0730100 | tRNA pseudouridine synthase D, putative | 4**.**815 | 2**.**929 | 4**.**33E-02 |
| PF3D7_0801100 | 28S ribosomal RNA | 2**.**831 | 6**.**299 | 4**.**40E-02 |
| PF3D7_1430400 | autophagy protein 5, putative | -1**.**399 | 6**.**717 | 4**.**40E-02 |
| PF3D7_0813700 | ABC transporter F family member 1 | 4**.**255 | 4**.**044 | 4**.**41E-02 |
| PF3D7_1416800 | lysine--tRNA ligase, putative | 5**.**433 | 3**.**163 | 4**.**41E-02 |
| PF3D7_0626700 | ATPase | -2**.**422 | 6**.**747 | 4**.**41E-02 |
| PF3D7_0630300 | DNA polymerase epsilon catalytic subunit A, putative | 5**.**912 | 4**.**006 | 4**.**44E-02 |
| PF3D7_0316600 | formate-nitrite transporter | 1**.**258 | 8**.**772 | 4**.**54E-02 |
| PF3D7_0806100 | conserved Plasmodium protein, unknown function | 3**.**110 | 5**.**050 | 4**.**58E-02 |
| PF3D7_0830700 | Plasmodium exported protein (hyp9), unknown function | 1**.**694 | 5**.**716 | 4**.**63E-02 |
| PF3D7_0204600 | 5'-3' exonuclease, putative | 3**.**701 | 3**.**116 | 4**.**67E-02 |
| PF3D7_1218200 | symplekin domain-containing protein, putative | -1**.**239 | 8**.**269 | 4**.**70E-02 |
| PF3D7_1038000 | antigen UB05 | -1**.**054 | 9**.**045 | 4**.**70E-02 |
| PF3D7_0813900 | 40S ribosomal protein S16, putative | -1**.**461 | 9**.**484 | 4**.**70E-02 |
| PF3D7_0321600 | ATP-dependent RNA helicase DDX42, putative | 2**.**787 | 4**.**373 | 4**.**70E-02 |
| PF3D7_0322700 | conserved Plasmodium protein, unknown function | -1**.**169 | 7**.**930 | 4**.**71E-02 |
| PF3D7_0504000 | cation transporting P-ATPase | 2**.**233 | 6**.**332 | 4**.**76E-02 |
| PF3D7_0623900 | ribonuclease H2 subunit A, putative | 5**.**504 | 4**.**469 | 4**.**76E-02 |
| PF3D7_1024000 | conserved Plasmodium protein, unknown function | 3**.**465 | 4**.**767 | 4**.**78E-02 |
| PF3D7_0925900 | lipocalin | -1**.**118 | 9**.**507 | 4**.**82E-02 |
| PF3D7_0702500 | Plasmodium exported protein, unknown function | 4**.**098 | 7**.**595 | 4**.**82E-02 |

| PF3D7_1218400 | triose or hexose phosphate/phosphate translocator, putative | -1**.**249 | 7**.**606 | 4**.**85E-02 |
| --- | --- | --- | --- | --- |
| PF3D7_1206800 | conserved Plasmodium protein, unknown function | 3**.**814 | 3**.**576 | 4**.**85E-02 |
| PF3D7_0404500 | 6-cysteine protein P52 | 6**.**317 | 2**.**737 | 4**.**85E-02 |
| PF3D7_1003700 | MKT1 domain-containing protein, putative | 5**.**912 | 3**.**817 | 4**.**86E-02 |
| PF3D7_1016300 | GBP130 protein | 2**.**215 | 10**.**960 | 4**.**94E-02 |
| PF3D7_0519700 | FoP domain-containing protein, putative | -1**.**015 | 9**.**158 | 5**.**04E-02 |
| PF3D7_1428500 | protein kinase, putative | -1**.**605 | 7**.**920 | 5**.**04E-02 |
| PF3D7_1022800 | 4-hydroxy-3-methylbut-2-en-1-yl diphosphate synthase (ferredoxin) | 6**.**806 | 4**.**236 | 5**.**04E-02 |
| PF3D7_1146800 | conserved Plasmodium protein, unknown function | 5**.**296 | 3**.**673 | 5**.**04E-02 |
| PF3D7_1452700 | U1 snRNP-associated protein, putative | 2**.**766 | 4**.**527 | 5**.**04E-02 |
| PF3D7_1018700 | conserved Plasmodium protein, unknown function | -0**.**978 | 8**.**756 | 5**.**14E-02 |
| PF3D7_1002400 | transformer-2 protein homolog beta, putative | -1**.**004 | 8**.**712 | 5**.**16E-02 |
| PF3D7_1423500 | conserved Plasmodium protein, unknown function | 2**.**221 | 6**.**825 | 5**.**16E-02 |
| PF3D7_1220900 | heterochromatin protein 1 | -1**.**187 | 8**.**092 | 5**.**23E-02 |
| PF3D7_1401800 | choline kinase | 2**.**738 | 4**.**888 | 5**.**23E-02 |
| PF3D7_0907600 | translation initiation factor SUI1, putative | -1**.**437 | 6**.**923 | 5**.**29E-02 |
| PF3D7_1456300 | conserved Plasmodium protein, unknown function | 2**.**808 | 3**.**308 | 5**.**31E-02 |
| PF3D7_0411400 | DEAD box ATP-dependent RNA helicase, putative | 1**.**643 | 5**.**444 | 5**.**36E-02 |
| PF3D7_1401400 | early transcribed membrane protein 14**.**1 | -1**.**221 | 9**.**446 | 5**.**36E-02 |
| PF3D7_0707500 | conserved Plasmodium protein, unknown function | -1**.**869 | 6**.**876 | 5**.**36E-02 |
| PF3D7_0109600 | cold-shock protein, putative | 3**.**437 | 1**.**571 | 5**.**39E-02 |
| PF3D7_0419900 | phosphatidylinositol 4-kinase, putative | 1**.**121 | 9**.**673 | 5**.**39E-02 |
| PF3D7_1314500 | transmembrane emp24 domain-containing protein, putative | -1**.**960 | 5**.**996 | 5**.**46E-02 |
| PF3D7_1409900 | cytidine diphosphate-diacylglycerol synthase | 1**.**130 | 7**.**469 | 5**.**46E-02 |
| PF3D7_1248600 | conserved Plasmodium protein, unknown function | 5**.**160 | 3**.**808 | 5**.**49E-02 |
| PF3D7_0808000 | conserved Plasmodium protein, unknown function | 4**.**061 | 2**.**503 | 5**.**51E-02 |
| PF3D7_1250300 | vacuolar protein sorting-associated protein 26, putative | 3**.**677 | 4**.**681 | 5**.**59E-02 |
| PF3D7_1460700 | 60S ribosomal protein L27 | -1**.**335 | 10**.**219 | 5**.**61E-02 |
| PF3D7_0416900 | conserved Plasmodium protein, unknown function | -1**.**019 | 9**.**097 | 5**.**64E-02 |
| PF3D7_1372100 | Plasmodium exported protein (PHISTb), unknown function | 7**.**478 | 4**.**277 | 5**.**64E-02 |
| PF3D7_1116100 | serine esterase, putative | 3**.**716 | 4**.**919 | 5**.**64E-02 |
| PF3D7_0202100 | liver stage associated protein 2 | -2**.**699 | 7**.**894 | 5**.**64E-02 |
| PF3D7_1360700 | E3 SUMO-protein ligase PIAS, putative | -1**.**164 | 7**.**089 | 5**.**64E-02 |
| PF3D7_1337000 | mitochondrial intermediate peptidase, putative | 5**.**218 | 2**.**503 | 5**.**64E-02 |
| PF3D7_1336200 | conserved Plasmodium protein, unknown function | 4**.**242 | 4**.**999 | 5**.**66E-02 |
| PF3D7_0407000 | conserved Plasmodium protein, unknown function | -1**.**615 | 5**.**584 | 5**.**70E-02 |
| PF3D7_0424600 | Plasmodium exported protein (PHISTb) | -1**.**297 | 8**.**456 | 5**.**76E-02 |
| PF3D7_1430300 | acid phosphatase, putative | -1**.**581 | 8**.**534 | 5**.**81E-02 |
| PF3D7_0313100 | ubiquitin-protein ligase, putative | 2**.**278 | 5**.**395 | 5**.**85E-02 |
| PF3D7_0916700 | RNA-binding protein musashi, putative | -1**.**535 | 8**.**258 | 5**.**85E-02 |
| PF3D7_1038400 | gametocyte-specific protein | 5**.**563 | 6**.**913 | 5**.**86E-02 |
| PF3D7_0616300 | conserved Plasmodium protein, unknown function | -3**.**835 | 2**.**810 | 5**.**96E-02 |
| PF3D7_0820100 | RNA-binding protein, putative | 2**.**824 | 2**.**245 | 6**.**00E-02 |
| PF3D7_0919000 | nucleosome assembly protein | -1**.**286 | 9**.**140 | 6**.**03E-02 |
| PF3D7_1356600 | regulator of chromosome condensation, putative | 2**.**453 | 5**.**391 | 6**.**05E-02 |
| PF3D7_1364800 | DNA-directed RNA polymerases I, II, and III subunit RPABC1, putative | -1**.**509 | 6**.**593 | 6**.**09E-02 |
| PF3D7_0815800 | vacuolar protein sorting-associated protein 9, putative | 3**.**338 | 6**.**535 | 6**.**14E-02 |
| PF3D7_1001600 | exported lipase 2 | 3**.**725 | 5**.**338 | 6**.**24E-02 |
| PF3D7_0821500 | ribosomal RNA small subunit methyltransferase NEP1, putative | -2**.**299 | 5**.**866 | 6**.**25E-02 |
| PF3D7_0523700 | conserved Plasmodium membrane protein, unknown function | 5**.**575 | 2**.**655 | 6**.**31E-02 |
| PF3D7_1473100 | GTPase-activating protein, putative | 2**.**689 | 4**.**012 | 6**.**43E-02 |
| PF3D7_1024700 | conserved protein, unknown function | 4**.**542 | 3**.**158 | 6**.**43E-02 |
| PF3D7_1357000 | elongation factor 1-alpha | -1**.**266 | 10**.**738 | 6**.**44E-02 |
| PF3D7_1474300 | DNA repair metallo-beta-lactamase protein, putative | 3**.**849 | 3**.**579 | 6**.**44E-02 |
| PF3D7_0914200 | phospholipid or glycerol acyltransferase, putative | -1**.**251 | 8**.**010 | 6**.**48E-02 |
| PF3D7_1132400 | conserved Plasmodium membrane protein, unknown function | 2**.**892 | 4**.**270 | 6**.**48E-02 |
| PF3D7_1118200 | heat shock protein 90, putative | 2**.**409 | 4**.**863 | 6**.**52E-02 |
| PF3D7_0722800 | conserved protein, unknown function | 1**.**953 | 5**.**412 | 6**.**53E-02 |
| PF3D7_1369000 | glycosylphosphatidylinositol anchor attachment 1 protein, putative | -2**.**201 | 5**.**284 | 6**.**62E-02 |
| PF3D7_1021700 | VPS13 domain-containing protein, putative | 3**.**604 | 7**.**806 | 6**.**73E-02 |
| PF3D7_0908700 | tRNA pseudouridine synthase, putative | 4**.**529 | 1**.**589 | 6**.**77E-02 |
| PF3D7_0302100 | serine/threonine protein kinase | 1**.**073 | 8**.**314 | 6**.**88E-02 |
| PF3D7_1475400 | cysteine repeat modular protein 4 | 4**.**715 | 4**.**192 | 6**.**88E-02 |
| PF3D7_1113000 | conserved Plasmodium protein, unknown function | 3**.**627 | 4**.**192 | 6**.**94E-02 |
| PF3D7_1443900 | heat shock protein 90, putative | 3**.**000 | 5**.**273 | 7**.**00E-02 |
| PF3D7_0818900 | heat shock protein 70 | 1**.**523 | 12**.**647 | 7**.**00E-02 |
| PF3D7_1209400 | cytosolic iron-sulfur protein assembly protein 1, putative | 2**.**571 | 4**.**489 | 7**.**06E-02 |
| PF3D7_0321900 | cyclic amine resistance locus protein | -1**.**054 | 8**.**206 | 7**.**06E-02 |
| PF3D7_1415300 | RNA-binding protein Nova-1, putative | 5**.**025 | 4**.**779 | 7**.**15E-02 |
| PF3D7_1245100 | kinesin-13, putative | 3**.**517 | 4**.**071 | 7**.**15E-02 |

| PF3D7_1024600 | RAP protein, putative | 5**.**394 | 2**.**440 | 7**.**18E-02 |
| --- | --- | --- | --- | --- |
| PF3D7_0723000 | tRNAHis guanylyltransferase, putative | -2**.**862 | 3**.**507 | 7**.**18E-02 |
| PF3D7_1104800 | metabolite/drug transporter, putative | 4**.**168 | 3**.**581 | 7**.**21E-02 |
| PF3D7_0810000 | acyl-CoA binding protein, putative | -2**.**848 | 6**.**033 | 7**.**31E-02 |
| PF3D7_1352800 | vacuolar fusion protein MON1, putative | 1**.**792 | 5**.**696 | 7**.**36E-02 |
| PF3D7_0307700 | apicomplexan kinetochore protein 10, putative | 2**.**214 | 4**.**975 | 7**.**39E-02 |
| PF3D7_1145100 | coatomer subunit gamma, putative | 1**.**492 | 5**.**966 | 7**.**39E-02 |
| PF3D7_0629200 | DnaJ protein, putative | 1**.**846 | 6**.**123 | 7**.**39E-02 |
| PF3D7_0803600 | conserved Plasmodium protein, unknown function | -1**.**018 | 7**.**805 | 7**.**46E-02 |
| PF3D7_1439800 | vesicle-associated membrane protein, putative | 1**.**828 | 7**.**269 | 7**.**52E-02 |
| PF3D7_0107800 | double-strand break repair protein MRE11 | 6**.**177 | 3**.**532 | 7**.**52E-02 |
| PF3D7_1471100 | exported protein 2 | 1**.**064 | 9**.**325 | 7**.**52E-02 |
| PF3D7_1442600 | TRAP-like protein | 1**.**604 | 6**.**680 | 7**.**61E-02 |
| PF3D7_1104400 | thioredoxin-like mero protein | 0**.**997 | 8**.**462 | 7**.**65E-02 |
| PF3D7_0903300 | conserved protein, unknown function | 3**.**730 | 5**.**409 | 7**.**65E-02 |
| PF3D7_1322200 | protein STU2, putative | 2**.**716 | 6**.**072 | 7**.**66E-02 |
| PF3D7_1021800 | schizont egress antigen-1 | 4**.**185 | 6**.**137 | 7**.**66E-02 |
| PF3D7_0112200 | multidrug resistance-associated protein 1 | 3**.**099 | 6**.**138 | 7**.**66E-02 |
| PF3D7_0512200 | glutathione synthetase | 2**.**826 | 3**.**932 | 7**.**66E-02 |
| PF3D7_0815600 | eukaryotic translation initiation factor 3 subunit G, putative | -1**.**122 | 8**.**296 | 7**.**66E-02 |
| PF3D7_1026300 | conserved Plasmodium protein, unknown function | 4**.**376 | 3**.**901 | 7**.**66E-02 |
| PF3D7_1103100 | 60S acidic ribosomal protein P1, putative | -1**.**120 | 9**.**354 | 7**.**66E-02 |
| PF3D7_1333000 | 20 kDa chaperonin | 1**.**605 | 5**.**602 | 7**.**66E-02 |
| PF3D7_1412900 | ubiquitin-conjugating enzyme E2, putative | -1**.**572 | 8**.**413 | 7**.**66E-02 |
| PF3D7_1402200 | cytochrome c oxidase subunit ApiCOX19, putative | -4**.**790 | 2**.**742 | 7**.**72E-02 |
| PF3D7_0624600 | ISWI chromatin-remodeling complex ATPase | 0**.**922 | 10**.**660 | 7**.**75E-02 |
| PF3D7_1001900 | Plasmodium exported protein (hyp16), unknown function | -1**.**570 | 6**.**514 | 7**.**75E-02 |
| PF3D7_0913200 | elongation factor 1-beta | -1**.**044 | 9**.**832 | 7**.**78E-02 |
| PF3D7_1308700 | conserved Plasmodium protein, unknown function | 3**.**242 | 5**.**489 | 7**.**94E-02 |
| PF3D7_0813200 | CS domain protein, putative | 4**.**404 | 2**.**965 | 8**.**23E-02 |
| PF3D7_1013500 | phosphoinositide-specific phospholipase C | -1**.**146 | 7**.**495 | 8**.**39E-02 |
| PF3D7_0317600 | 40S ribosomal protein S11, putative | -1**.**187 | 9**.**798 | 8**.**46E-02 |
| PF3D7_1035900 | merozoites-associated armadillo repeats protein | 6**.**179 | 4**.**502 | 8**.**46E-02 |
| PF3D7_1012700 | NLI interacting factor-like phosphatase, putative | -1**.**220 | 6**.**884 | 8**.**46E-02 |
| PF3D7_1371300 | 28S ribosomal RNA | 3**.**425 | 6**.**724 | 8**.**54E-02 |
| PF3D7_1036500 | conserved Plasmodium protein, unknown function | 5**.**425 | 4**.**108 | 8**.**54E-02 |
| PF3D7_1012000 | RING zinc finger protein, putative | -1**.**034 | 7**.**329 | 8**.**54E-02 |
| PF3D7_0418600 | regulator of chromosome condensation, putative | 4**.**715 | 5**.**073 | 8**.**54E-02 |
| PF3D7_1333200 | ubiquitin-activating enzyme | 8**.**003 | 5**.**069 | 8**.**54E-02 |
| PF3D7_0827900 | protein disulfide-isomerase | 1**.**326 | 9**.**095 | 8**.**54E-02 |
| PF3D7_0905400 | high molecular weight rhoptry protein 3 | 5**.**625 | 5**.**332 | 8**.**54E-02 |
| PF3D7_1222300 | endoplasmin, putative | 2**.**145 | 6**.**951 | 8**.**58E-02 |
| PF3D7_1469400 | nucleoside transporter 3, putative | 3**.**487 | 5**.**121 | 8**.**58E-02 |
| PF3D7_0925200 | ribosomal RNA-processing protein 8, putative | -1**.**221 | 7**.**414 | 8**.**62E-02 |
| PF3D7_0412000 | LITAF-like zinc finger protein, putative | 2**.**625 | 5**.**112 | 8**.**62E-02 |
| PF3D7_0728900 | RNA-binding protein, putative | -1**.**464 | 6**.**322 | 8**.**62E-02 |
| PF3D7_1250500 | AP-3 complex subunit sigma, putative | 4**.**210 | 3**.**267 | 8**.**65E-02 |
| PF3D7_0907000 | conserved Plasmodium protein, unknown function | -1**.**797 | 5**.**775 | 8**.**65E-02 |
| PF3D7_0818200 | 14-3-3 protein | -1**.**044 | 9**.**261 | 8**.**65E-02 |
| PF3D7_0106800 | ras-related protein Rab-5C | -3**.**336 | 3**.**318 | 8**.**67E-02 |
| PF3D7_1476500 | probable protein, unknown function | 5**.**550 | 3**.**624 | 8**.**68E-02 |
| PF3D7_1120100 | phosphoglycerate mutase, putative | -1**.**103 | 9**.**855 | 8**.**68E-02 |
| PF3D7_0306900 | 40S ribosomal protein S23, putative | -0**.**981 | 9**.**767 | 8**.**68E-02 |
| PF3D7_1359600 | conserved Plasmodium protein, unknown function | 3**.**942 | 6**.**248 | 8**.**68E-02 |
| PF3D7_0314700 | RING finger protein RNF1 | 4**.**420 | 4**.**923 | 8**.**70E-02 |
| PF3D7_1323600 | conserved protein, unknown function | 5**.**832 | 3**.**390 | 8**.**79E-02 |
| PF3D7_1366800 | phosphatidylserine synthase | 1**.**825 | 7**.**315 | 8**.**88E-02 |
| PF3D7_1113400 | ubiquitin domain-containing protein DSK2, putative | -1**.**022 | 7**.**923 | 8**.**95E-02 |
| PF3D7_0517000 | 60S ribosomal protein L12, putative | -1**.**054 | 9**.**236 | 8**.**95E-02 |
| PF3D7_1344200 | endoplasmic reticulum chaperone GRP170 | 1**.**086 | 8**.**466 | 8**.**99E-02 |
| PF3D7_0826100 | HECT-like E3 ubiquitin ligase, putative | 0**.**977 | 10**.**333 | 9**.**00E-02 |
| PF3D7_1345200 | rhomboid protease ROM6, putative | 2**.**992 | 3**.**443 | 9**.**05E-02 |
| PF3D7_1305900 | conserved Plasmodium protein, unknown function | 5**.**247 | 3**.**934 | 9**.**05E-02 |
| PF3D7_0804000 | cactin homolog, putative | -1**.**003 | 9**.**186 | 9**.**09E-02 |
| PF3D7_0219200 | 40S ribosomal protein S30 | -1**.**244 | 9**.**059 | 9**.**12E-02 |
| PF3D7_0808200 | plasmepsin X | 3**.**925 | 3**.**699 | 9**.**21E-02 |
| PF3D7_0104400 | 4-hydroxy-3-methylbut-2-enyl diphosphate reductase | 4**.**896 | 3**.**720 | 9**.**22E-02 |
| PF3D7_1022500 | citrate synthase, mitochondrial, putative | 4**.**371 | 4**.**039 | 9**.**22E-02 |
| PF3D7_0924100 | conserved Plasmodium protein, unknown function | 2**.**630 | 3**.**566 | 9**.**23E-02 |
| PF3D7_0730400 | IMP1-like protein, putative | -5**.**781 | 2**.**821 | 9**.**23E-02 |
| PF3D7_0816600 | chaperone protein ClpB1 | 2**.**757 | 4**.**964 | 9**.**26E-02 |
| PF3D7_0625600 | poly(A) polymerase PAP, putative | 1**.**194 | 7**.**375 | 9**.**30E-02 |
| PF3D7_1106500 | conserved Plasmodium protein, unknown function | 1**.**395 | 6**.**037 | 9**.**30E-02 |
| PF3D7_1330400 | ER lumen protein retaining receptor 1, putative | -1**.**251 | 6**.**633 | 9**.**34E-02 |
| PF3D7_1341900 | V-type proton ATPase subunit D, putative | -1**.**095 | 7**.**536 | 9**.**37E-02 |
| PF3D7_0811900 | RNA-binding protein, putative | 3**.**306 | 3**.**720 | 9**.**37E-02 |
| PF3D7_1239900 | vacuolar protein sorting-associated protein 16, putative | -1**.**263 | 6**.**398 | 9**.**38E-02 |
| PF3D7_0710600 | 60S ribosomal protein L34 | -1**.**097 | 9**.**482 | 9**.**41E-02 |
| PF3D7_1248700 | conserved protein, unknown function | 0**.**968 | 10**.**950 | 9**.**41E-02 |
| PF3D7_1025500 | conserved Plasmodium protein, unknown function | 2**.**907 | 4**.**344 | 9**.**42E-02 |
| PF3D7_0910200 | conserved Plasmodium protein, unknown function | 1**.**356 | 8**.**394 | 9**.**43E-02 |
| PF3D7_1004000 | 60S ribosomal protein L13, putative | -1**.**278 | 9**.**473 | 9**.**43E-02 |
| PF3D7_0731800 | alpha/beta hydrolase, putative | -2**.**621 | 6**.**802 | 9**.**43E-02 |
| PF3D7_1324400 | PRELI domain-containing protein, putative | -1**.**020 | 7**.**842 | 9**.**43E-02 |
| PF3D7_1362400 | calpain | -1**.**179 | 8**.**073 | 9**.**44E-02 |
| PF3D7_1122500 | protein KIC10 | 3**.**732 | 3**.**201 | 9**.**44E-02 |
| PF3D7_1226800 | ataxin-3, putative | -2**.**265 | 4**.**864 | 9**.**46E-02 |
| PF3D7_1477600 | surface-associated interspersed protein 14**.**1 (SURFIN 14**.**1) | 9**.**328 | 5**.**060 | 9**.**47E-02 |
| PF3D7_0201700 | DnaJ protein, putative | -1**.**312 | 8**.**278 | 9**.**47E-02 |
| PF3D7_1460500 | conserved Plasmodium protein, unknown function | 3**.**132 | 4**.**030 | 9**.**56E-02 |
| PF3D7_0315900 | conserved Plasmodium protein, unknown function | 6**.**780 | 2**.**879 | 9**.**56E-02 |
| PF3D7_0316800 | 40S ribosomal protein S15A, putative | -1**.**025 | 9**.**722 | 9**.**56E-02 |
| PF3D7_0911700 | GTP-binding protein, putative | 5**.**639 | 5**.**464 | 9**.**56E-02 |
| PF3D7_1019400 | 60S ribosomal protein L30e, putative | -1**.**197 | 8**.**690 | 9**.**59E-02 |
| PF3D7_1205000 | conserved protein, unknown function | -1**.**713 | 5**.**844 | 9**.**59E-02 |
| PF3D7_1469900 | protein MGET | -3**.**576 | 4**.**778 | 9**.**61E-02 |
| PF3D7_1365500 | aminomethyltransferase, mitochondrial, putative | 2**.**977 | 4**.**150 | 9**.**72E-02 |
| PF3D7_0812900 | conserved Plasmodium protein, unknown function | 4**.**519 | 3**.**693 | 9**.**72E-02 |
| PF3D7_0614100 | filamin domain-containing protein, putative | -3**.**201 | 3**.**883 | 9**.**72E-02 |
| PF3D7_0203300 | ERCC1 nucleotide excision repair protein, putative | -2**.**200 | 4**.**459 | 9**.**78E-02 |
| PF3D7_1122200 | JmjC domain-containing protein 3 | -1**.**247 | 6**.**441 | 9**.**78E-02 |
| PF3D7_1217100 | DNA topoisomerase 6 subunit A | 2**.**552 | 2**.**816 | 9**.**78E-02 |
| PF3D7_1369600 | conserved Plasmodium protein, unknown function | 3**.**154 | 3**.**299 | 9**.**84E-02 |
| PF3D7_0305700 | ubiquitin-conjugating enzyme MMS2, putative | -1**.**119 | 7**.**011 | 9**.**91E-02 |
| PF3D7_0509600 | asparagine--tRNA ligase | 2**.**866 | 4**.**295 | 9**.**95E-02 |

BTH, concurrent cerebral malaria and severe malarial anemia cases; CM, cerebral malaria; SMA, severe malarial anemia; logFC, log-fold-change; logCPM, log counts per million; FDR, false discovery rate.

| **Table S19. Significant Gene Ontology (GO) biological processes and cellular component results for concurrently infected cases compared to controls without a history of CM.** | | |
| --- | --- | --- |
| Gene Ontology—Biological Processes | **P-Value** | **FDR** |
| nucleobase-containing compound metabolic process (GO:0006139) | 3.35E-05 | 2.49E-02 |
| RNA metabolic process (GO:0016070) | 1.27E-05 | 2.82E-02 |
| gene expression (GO:0010467) | 6.61E-05 | 2.94E-02 |
| nucleic acid metabolic process (GO:0090304) | 8.09E-05 | 3.00E-02 |
| cellular aromatic compound metabolic process (GO:0006725) | 5.55E-05 | 3.09E-02 |
| RNA processing (GO:0006396) | 2.82E-05 | 3.13E-02 |
| organic cyclic compound metabolic process (GO:1901360) | 1.31E-04 | 3.63E-02 |
| cellular nitrogen compound metabolic process (GO:0034641) | 1.25E-04 | 3.97E-02 |
| heterocycle metabolic process (GO:0046483) | 1.64E-04 | 4.05E-02 |
| cellular component biogenesis (GO:0044085) | 3.36E-04 | 7.47E-02 |
| Gene Ontology—Cellular Components Ontology Term- | **P-Value** | **FDR** |
| host cell (GO:0043657) | 9.55E-18 | 3.08E-15 |
| host cellular component (GO:0018995) | 9.55E-18 | 6.16E-15 |
| host cell part (GO:0033643) | 4.85E-12 | 1.04E-09 |
| host cell cytoplasm (GO:0030430) | 4.78E-11 | 5.14E-09 |
| host intracellular region (GO:0043656) | 4.71E-11 | 6.08E-09 |
| host intracellular part (GO:0033646) | 4.18E-11 | 6.75E-09 |
| host cell cytoplasm part (GO:0033655) | 1.94E-10 | 1.79E-08 |
| Maurer’s cleft (GO:0020036) | 1.28E-08 | 1.04E-06 |
| nuclear protein-containing complex (GO:0140513) | 2.11E-04 | 1.24E-02 |
| host cell surface (GO:0044228) | 2.08E-04 | 1.34E-02 |
| protein-containing complex (GO:0032991) | 1.94E-04 | 1.39E-02 |
| extracellular organelle (GO:0043230) | 8.70E-04 | 4.32E-02 |
| extracellular membrane-bounded organelle (GO:0065010) | 8.31E-04 | 4.47E-02 |
| extracellular region (GO:0005576) | 1.27E-03 | 5.85E-02 |
| cellular anatomical entity (GO:0110165) | 1.66E-03 | 7.15E-02 |
| host cell periphery (GO:0044538) | 1.85E-03 | 7.45E-02 |
| catalytic complex (GO:1902494) | 2.72E-03 | 9.75E-02 |
| extracellular vesicle (GO:1903561) | 2.60E-03 | 9.87E-02 |

CM, cerebral malaria; FDR, false discovery rate.

**ADDITIONAL REFERENCES**

1. Bozdech Z, Llinás M, Pulliam BL, Wong ED, Zhu J, DeRisi JL. The Transcriptome of the Intraerythrocytic Developmental Cycle of Plasmodium falciparum. PLoS Biol. 2003 Oct;1(1):e5.
